# Systematic review and meta-analysis of the global prevalence and infection risk factors of *Trichomonas vaginalis*

**DOI:** 10.1051/parasite/2025051

**Published:** 2025-08-27

**Authors:** Wenjie Tian, Yuhua Li, Yani Zhang, Yiming Zhang, Yiran Qin, Yalin Han, Dongxian Li, Shuai Wang, Zhenke Yang, Xiaowei Tian, Xuefang Mei, Zhenchao Zhang

**Affiliations:** 1 Department of Pathogenic Biology, School of Basic Medical Sciences, Xinxiang Medical University Xinxiang Henan 453003 PR China; 2 Xinxiang Key Laboratory of Pathogenic Biology, School of Basic Medical Sciences, Xinxiang Medical University Xinxiang Henan 453003 PR China

**Keywords:** Systematic review, *T. vaginalis*, Prevalence, Risk factors

## Abstract

Trichomoniasis is a globally prevalent sexually transmitted disease; however, comprehensive data on its global prevalence and incidence are relatively limited. In this study, we systematically retrieved relevant articles from PubMed, Google Scholar, Scopus, Ovid–Medline, and Web of Science to analyze the prevalence of *Trichomonas vaginalis* and its association with various infection risk factors. Among 5,437 publications released between 1992 and 2023, 425 articles focusing on the epidemiology of *T. vaginalis* were identified. The results revealed a global prevalence rate of *T. vaginalis* of 8% (95% CI: 7%–10%), with country-specific rates ranging from 1% to 35%. The prevalence of *T. vaginalis* was significantly higher in the behavioral subgroups, including smoking, drug use, and not using condoms, compared to the non-infection group, with a pooled odds ratio (OR) of 1.67 (95% CI: 1.39–2.0). Furthermore, the prevalence of *T. vaginalis* was significantly higher in the group with other sexually transmitted infections (STIs), including HIV, HSV, and *Chlamydia* infection compared to the non-infection group, with a pooled OR of 2.01 (95% CI: 1.48–2.72). Finally, socioeconomic factors such as being unmarried, having a low income, and unstable employment were associated with an increased risk of *T. vaginalis* infection, with a pooled OR of 1.36 (95% CI: 1.10–1.66). This study has significant public health relevance for the prevention and control of trichomoniasis.

## Abstract (fr)

La trichomonase est une maladie sexuellement transmissible répandue dans le monde. Cependant, les données complètes sur sa prévalence et son incidence mondiales sont relativement limitées. Dans cette étude, nous avons systématiquement extrait des articles pertinents de PubMed, Google Scholar, Scopus, Ovid-Medline et Web of Science afin d’analyser la prévalence de *Trichomonas vaginalis* et son association avec divers facteurs de risque d’infection. Parmi 5 437 publications publiées entre 1992 et 2023, 425 articles portant sur l’épidémiologie de *T. vaginalis* ont été identifiés. Les résultats ont révélé un taux de prévalence mondial de *T. vaginalis* de 8% (IC à 95 % : 7% - 10%), avec des taux spécifiques à chaque pays allant de 1 % à 35 %. La prévalence de *T. vaginalis* était significativement plus élevée dans les sous-groupes comportementaux incluant le tabagisme, la consommation de drogues et la non-utilisation de préservatifs par rapport au groupe non infecté, avec un rapport de cotes (RC) groupé de 1,67 (IC à 95 % : 1,39 - 2,0). De plus, la prévalence de *T. vaginalis* était significativement plus élevée dans le groupe avec d’autres infections sexuellement transmissibles (IST) incluant le VIH, le VHS et l’infection à *Chlamydia* par rapport au groupe non infecté, avec un RC groupé de 2,01 (IC à 95 % : 1,48 - 2,72). Enfin, des facteurs socio-économiques tels que le célibat, un faible revenu et un emploi instable étaient associés à un risque accru d’infection à *T. vaginalis*, avec un RC groupé de 1,36 (IC à 95 % : 1,10 - 1,66). Cette étude est pertinente en matière de santé publique pour la prévention et le contrôle de la trichomonase.

## Introduction

*Trichomonas vaginalis* is an extracellular flagellated protozoan that parasitizes the human reproductive and urinary tracts. Infection with *T. vaginalis* can lead to trichomoniasis, a sexually transmitted disease (STD) that is highly prevalent worldwide [60]. A survey conducted by the World Health Organization (WHO) in 2016 estimated the prevalence of trichomoniasis among women globally to be 5.3% (95% CI: 4.0–7.2) [88]. Among high-risk female populations, the prevalence of *T. vaginalis* was close to 12.9%–14.4%, higher than the prevalence of *Neisseria gonorrhoeae* and *Chlamydia trachomatis* [80, 96]. This parasitic infection commonly causes vaginitis, cervicitis, urethritis, and prostatitis. Some studies have shown that *T. vaginalis* could increase the risk of HPV and HIV infections [57, 109]. Moreover, *T. vaginalis* excretory secretory proteins reduce semen quality and *T. vaginalis* infection can lead to infertility [108, 109]. Due to the high prevalence of asymptomatic *T. vaginalis* infection, there is a lack of consensus regarding the true prevalence and persistence of the parasite in the reproductive tract [78]. Although trichomoniasis is a global public health concern with pervasive impacts, its prevalence in many countries remains unclear and is often overlooked in clinical practice [80, 90]. Consequently, it is imperative to summarize and analyze the literature on the epidemiology of *T. vaginalis* in order to accurately assess the infection situation in each country. Furthermore, the risk factors for *T. vaginalis* infection remain poorly defined, with the occurrence of infection potentially involving a wide range of risk factors, including lifestyle habits, socioeconomic status, and involvement in commercial sex work [64, 108].

The objective of this study was to conduct a comprehensive review of the published literature on the prevalence of *T. vaginalis*, collate epidemiological data on this pathogen, and summarize the distribution of the parasite in different countries. Given the potential influence of a multitude of factors on the occurrence of *T. vaginalis* infection, we conducted a meta-analysis of diverse populations to identify the additional risk factors, beyond those associated with commercial sex, which may contribute to the epidemic of trichomoniasis. This analysis was conducted with the aim of providing further insight into the global epidemic of *T. vaginalis*, and the study will contribute to improving prevention and control of trichomoniasis in the future.

## Materials and methods

### Eligibility criteria, information sources, and search strategy

In accordance with the PRISMA guidelines for systematic reviews and meta-analyses, two authors independently conducted searches of PubMed, Google Scholar, Scopus, Ovid–Medline, and Web of Science to identify relevant articles. The following medical subject headings were used alone or in combination: “*T. vaginalis*” or “*vaginalis, Trichomonas*” and “Prevalences” or “Period Prevalence” or “Period Prevalences” or “Prevalence, Period” or “Point Prevalence” or “Point Prevalences” or “Prevalence, Point”. The retrieved articles were manually reviewed by two independent researchers, who checked the titles, abstracts, and full texts, and removed irrelevant articles and duplicates. The potential factors influencing the prevalence of *T. vaginalis* were identified from 425 articles and classified into socioeconomic variables, behavioral variables, and sexually transmitted infection variables.

This systematic review was registered in the International Prospective Register of Systematic Reviews (PROSPERO, https://www.crd.york.ac.uk/prospero) with the registration ID CRD42023471855.

### Inclusion criteria for epidemiological investigations

(1) Original peer-reviewed article with specific geographic information. (2) Cross-sectional study on the prevalence of *T. vaginalis.* (3) Article published in English between July 1992 and June 2023. (4) Article with access to abstract and full text. (5) Articles contains accurate statistical data on infection or non-infection with *T. vaginalis*.

### Exclusion criteria for epidemiological investigations

(1) Article repeated or unrelated to *T. vaginalis.* (2) A total sample size and an exact number of positive cases not included in the article. (3) Article not published in English or did not focus on *T. vaginalis* infections. (4) Article did not study *T. vaginalis* infections in humans.

### Inclusion criteria for risk factor analysis

(1) Article contains statistical data on some factors influencing *T. vaginalis* infection. (2) Clear data sources in the article. (3) Latest and most comprehensive article was selected for literature with repeated relevant data.

### Exclusion criteria for risk factor analysis

(1) Risk factors of *T. vaginalis* infection not mentioned in the article, or relevant data are incomplete. (2) Only some potential risk factors in people infected with *T. vaginalis* are reported, while there are no data related to people without *T. vaginalis* infection in the article. (3) Sample size was less than 20. (4) Article was a non-population study.

### Quality assessment and data analysis for epidemiological investigations

As previously reported [109], the quality of the epidemiological investigations was evaluated by Newcastle–Ottawa scale (NOS), with a total score of 9 points, and the articles with a score ≥6 points were included in statistics. Stata17 software was used to analyze the data from the selected epidemiological investigations, and then a forest plot was drawn. A heterogeneity analysis was performed by *I*^2^ test. If *p* < 0.05 or *I*^2^ ≥ 50%, there was heterogeneity among the research, and the random-effect model should be used to further analyze the origin of heterogeneity. In order to assess the impact of different risk factors on the prevalence of *T. vaginalis*, we analyzed the OR values and evaluated the significance of differences based on the *p-*value.

## Results

### Literature inclusion subsection

**Figure 1:**
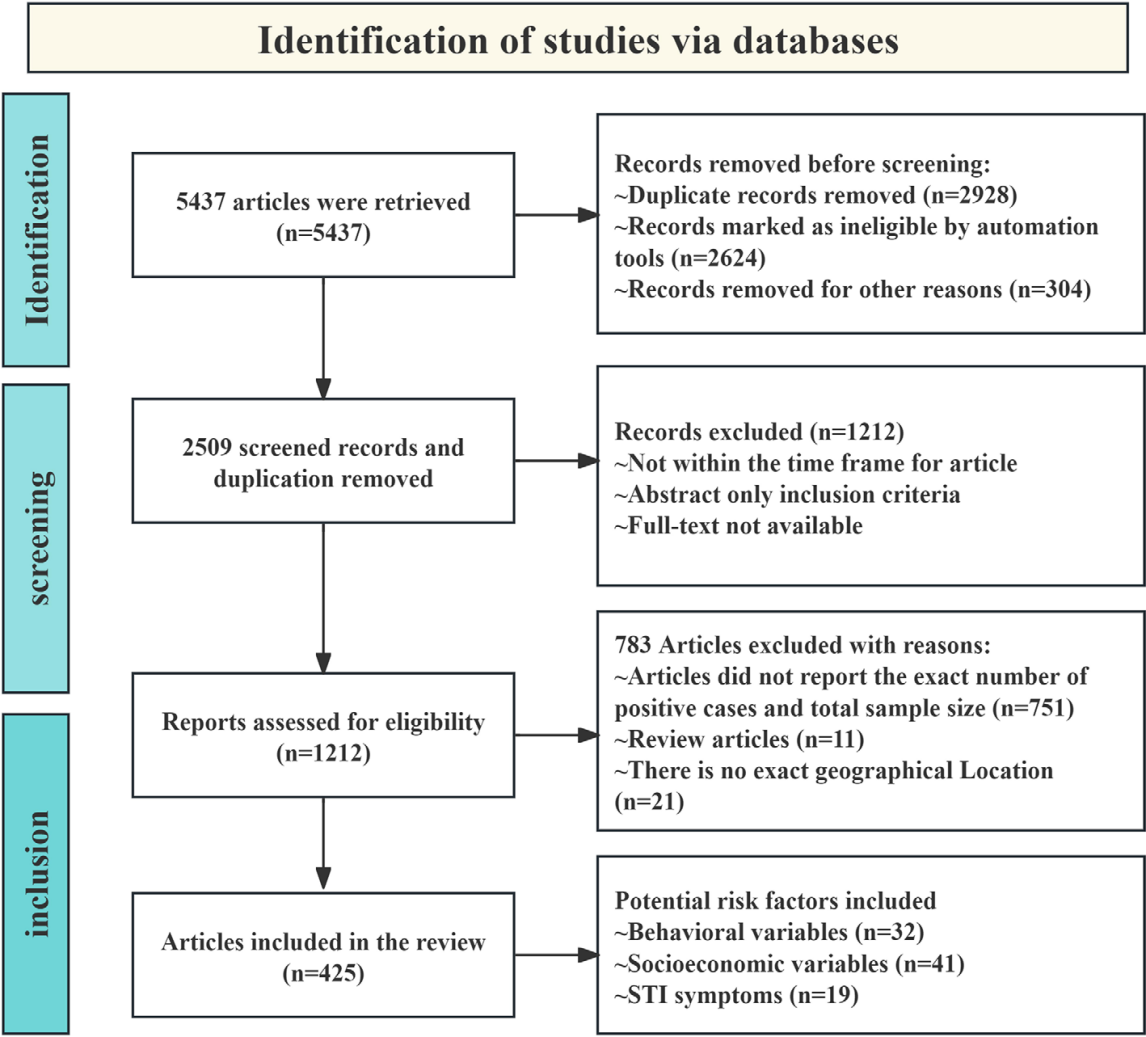
Flow diagram of article retrieval, identification, classification, and inclusion.

The search process is depicted in the PRISMA flowchart, which shows that a total of 5,437 records were retrieved from the selected databases (Fig. 1). Of the retrieved articles, 425 were selected based on the inclusion and exclusion criteria (Fig. 1). The articles on epidemiological investigations were sourced from 86 countries across six continents. Of the 425 articles, 126 were identified from Asian countries, 130 from African countries, 53 from North American countries, 49 from European countries, 40 from South American countries, and 27 from Oceania countries.

In this study, 92 articles were included in the analysis of risk factors with *T. vaginalis* infection, according to the inclusion and exclusion criteria. Of these, 41 articles were included in the socioeconomic variables group, 32 articles in the behavioral habits group, and 19 articles in the STIs group.

### Global prevalence of *T. vaginalis*

By reviewing these epidemiological articles, we found that the global prevalence of *T. vaginalis* was 8% (95% CI: 7%–10%), and the prevalence of *T. vaginalis* varied greatly in different parts of the world. Regionally, the prevalence was 3% (95% CI: 3%–4%) in Europe, 11% (95% CI: 8%–15%) in North America, 8% (95% CI: 7%–9%) in Asia, 5% (95% CI: 1%–10%) in South America, 12% (95% CI: 10%–14%) in Africa, and 16% (95% CI: 8%–24%) in Oceania (Table 1 and Fig. S1).

**Table 1:** Subgroup analysis of the included studies on the global prevalence of *T. vaginalis*.

WHO region	No. Country	Sample	Infected	Pooled prevalence (95% CI)	Heterogeneity		
					*I* ^*2*^	τ^2^	*p*-value
Europe	18	614,932	3,489	3% (3%–4%)	99.37%	0.00	<0.01
North America	8	191,655	15,010	11% (8%–15%)	99.29%	0.00	<0.01
Asia	22	1,061,540	12,312	8% (7%–9%)	99.86%	0.00	<0.01
South America	7	55,335	8,625	5% (1%–10%)	96.86%	0.01	<0.01
Africa	27	134,198	17,875	12% (10%–14%)	99.37%	0.00	<0.01
Oceania	3	19,193	1,995	16% (8%–24%)	99.79%	0.01	<0.01
Overall	85	2,076,853	59,306	8% (7%–10%)	99.85%	0.00	<0.01

### Prevalence of *T. vaginalis* in countries around the world

**Figure 2:**
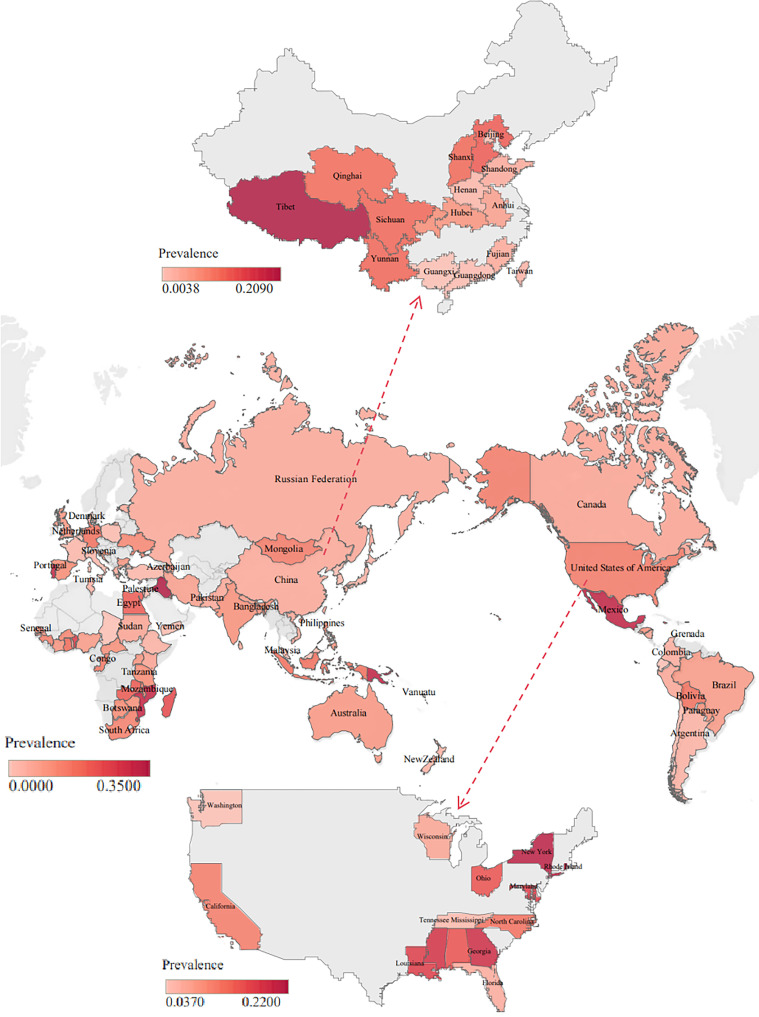
Map representing the global prevalence of *T. vaginalis* in different countries.

The epidemiological survey data on *T. vaginalis* in each country were subjected to individual meta-analysis in accordance with the inclusion and exclusion criteria and a world map with the prevalence of *T. vaginalis* illustrated using Tableau software (Fig. 2). The results demonstrated that the prevalence of *T. vaginalis* in each country ranged from 1% to 35% globally (Fig. S2), among these 85 countries with reported prevalence of *T. vaginalis* (Table 2 and Table S2).

**Table 2:** Subgroup analysis of *T. vaginalis* prevalence based on geographic area and reporting countries.

WHO region	No. Studies	Pooled prevalence (95% CI)	Heterogeneity		
			*I* ^*2*^	*Q*-value	*p*-value
**Asia region**	**126**	**8% (7%–9%)**	**99.69**	**325.86**	**0.00**
China	30	5% (4%–5%)	98.55	2005.68	0.00
Bangladesh	7	10% (1%–21%)	99.67	206.54	0.08
India	29	10% (9%–17%)	99.32	2389.93	0.00
Indonesia	5	25% (2%–48%)	95.96	97.60	0.00
Iran	16	7% (0%–14%)	99.97	311.16	0.04
Iraq	4	35% (8%–62%)	99.10	338.73	0.01
Korea	6	1% (0%–1%)	62.74	14.55	0.00
South Korea	4	2% (0%–3%)	90.05	31.96	0.03
Sri Lanka	4	5% (3%–7%)	80.19	16.59	0.00
Vietnam	5	5% (2%–8%)	96.65	95.61	0.00
Israel	3	3% (2%–8%)	91.78	12.17	0.20
Mongolia	3	27% (12%–66%)	93.22	29.40	0.01
Pakistan	2	5% (3%–6%)	0.10	0.44	0.00
**Africa region**	**130**	**12% (10%–14%)**	**99.29**	**140.12**	**0.00**
Egypt	3	20% (5%–36%)	96.73	83.16	0.01
Ghana	4	17% (1%–35%)	99.55	117.37	0.07
Madagascar	6	23% (15%–32%)	97.74	224.34	0.00
Senegal	3	9% (3%–22%)	99.14	75.10	0.13
Zimbabwe	2	10% (6%–14%)	76.39	4.23	0.00
Benin	2	25% (21%–28%)	52.75	2.12	0.00
Guinea Bissau	2	13% (1%–27%)	98.31	59.29	0.00
Burkina Faso	6	12% (3%–22%)	99.48	192.74	0.01
Kenya	18	6% (3%–9%)	99.01	438.73	0.00
Côte d’Ivoire	6	9% (4%–14%)	95.86	99.62	0.00
Nigeria	11	7% (4%–9%)	90.13	83.19	0.00
Sudan	2	6% (5%–18%)	99.49	195.95	0.28
South Africa	29	13% (9%–17%)	99.39	2461.02	0.00
Tanzania	13	11% (7%–15%)	98.72	562.94	0.00
Togo	2	5% (3%–7%)	36.40	1.57	0.00
Uganda	8	9% (5%–13%)	95.49	130.10	0.00
Zambia	3	20% (4%–35%)	98.94	231.19	0.01
Botswana	3	11% (3%–19%)	96.21	26.37	0.01
**North America**	**53**	**11% (8%–15%)**	**99.29**	**140.60**	**0.00**
United States	37	14% (11%–18%)	99.91	1061.85	0.00
Canada	5	6% (0%–12%)	99.36	97.71	0.07
Jamaica	3	21% (18%–25%)	35.32	1.55	0.00
Mexico	3	32% (26%–38%)	76.18	6.02	0.00
El Salvador	2	3% (1%–7%)	94.72	18.94	0.13
**South America**	**40**	**5% (1%**–**10%)**	**99.89**	**893.6**	**0.00**
Argentina	4	3% (3%–4%)	13.64	2.57	0.00
Colombia	3	4% (3%–10%)	99.23	11.57	0.28
Peru	6	5% (3%–7%)	80.34	20.82	0.00
Brazil	23	8% (5%–12%)	99.59	9261.95	0.00
**Europe**	**49**	**3% (3%**–**4%)**	**99.26**	**134.86**	**0.00**
United Kingdom	16	6% (2%–9%)	99.79	476.85	0.00
Türkiye	4	2% (0%–3%)	64.22	9.71	0.01
Italy	5	2% (1%–3%)	82.29	17.03	0.00
Netherlands	5	1% (0%–1%)	97.20	35.76	0.06
Russia	3	5% (3%–14%)	99.17	51.42	0.20
Estonia	2	5% (4%–15%)	95.58	22.61	0.27
Spain	3	11% (8%–30%)	99.98	220.2	0.25
Ukraine	2	12% (10%–13%)	2.21	1.02	0.00
**Oceania**	**27**	**16% (8%–24%)**	**99.71**	**370.65**	**0.00**
Papua New Guinea	9	32% (24%–41%)	95.26	143.27	0.00
Australia	13	10% (4%–16%)	99.71	343.49	0.00
New Zealand	2	3% (1%–4%)	64.42	2.81	0.00
Vanuatu	3	25% (21%–28%)	71.84	6.58	0.00

In some countries, the prevalence of *T. vaginalis* infection was found to be relatively low. Korea exhibited a prevalence of 1% (95% CI: 0%–1%) in Asia, and several European countries, including the Netherlands (1%, 95% CI: 0%–1%), Türkiye (2%, 95% CI: 0%–3%), and Italy (2%, 95% CI: 1%–3%), also showed lower prevalence [12, 13, 43, 45–48, 52, 53, 74–77, 101]. In contrast, certain countries exhibited higher infection rates. The prevalence of *T. vaginalis* was 35% (95% CI: 8%–62%) in Iraq, Asia. In Africa, the rates were 23% (95% CI: 15%–32%) in Madagascar and 25% (95% CI: 21%–28%) in Benin [2, 4, 5, 11, 38, 40, 44, 55, 56, 58, 82, 97]. In North America, the prevalence of *T. vaginalis* was 21% (95% CI: 18%–25%) in Jamaica, while the prevalence of this parasite was 32% (95% CI: 26%–38%) in Mexico [65, 92, 94, 105, 112, 113]. In Oceania, the prevalence of *T. vaginalis* was 32% (95% CI: 24%–41%) in Papua New Guinea, and 25% (95% CI: 21%–28%) in Vanuatu [10, 14, 33, 35, 62, 70–73, 79, 98–100, 102].

### Prevalence of *T. vaginalis* based on different detection methods

A total of 321 articles reported clear detection methods in analyzing the prevalence of *T. vaginalis.* We conducted a pooled analysis of the prevalence of *T. vaginalis* under different detection methods, including 77 articles about direct microscopy, 34 articles about the culture method, 61 articles about the swab detection method, 121 articles about polymerase chain reaction (PCR), 19 articles about nucleic acid amplification tests (NAAT), 5 articles about the One-Step-One-Minute (OSOM) detection method, and 4 articles about real-time PCR (qPCR).

The direct microscopy method involving 68,624 participants showed a pooled prevalence of 10% (95% CI: 7%–12%). The culture method involving 34,648 participants had a pooled prevalence of 13% (95% CI: 8%–18%). The swab detection method involving 83,113 participants presented a pooled prevalence of 10% (95% CI: 8%–12%). The PCR detection method involving 408,121 participants exhibited a pooled prevalence of 12% (95% CI: 10%–14%). The NAAT method involving 6,147 participants showed a pooled prevalence of 6% (95% CI: 2%–11%) for detecting *T. vaginalis.* The OSOM detection method involving 1,632 participants had a pooled prevalence of 11% (95% CI: 4%–19%). The qPCR method involving 3,222 participants presented a pooled prevalence of 8% (95% CI: 2%–14%). The higher detection rates with PCR and culture methods may be attributed to the higher sensitivity of these two detection techniques, as well as dynamic fluctuations in prevalence rates among different tested populations (Fig. S3).

### Prevalence of *T. vaginalis* in China and the USA

The prevalence of *T. vaginalis* in China and the United States was summarized through 67 articles from these two countries (Table S3). The prevalence rates of *T. vaginalis* in China and the USA were 7% and 14%, respectively. The prevalence in these two countries ranged from 1% to 25%, with higher prevalence in Tibet, China, and Rhode Island, USA. The articles displayed that the higher prevalence of *T. vaginalis* in the Tibet region of China might be related to economic level and hygiene practices [54]. The prevalence data for Rhode Island, USA were from women in a prison, and the higher prevalence in this state might be due to behavioral variables among female prison inmates.

### Potential risk factors for *T. vaginalis* in infection

#### Correlation between *T. vaginalis* infection and behavioral variables

A significant association was observed between various behavioral habits and the higher prevalence of *T. vaginalis.* In this study, three risk factors on behavioral variables were collected from the relevant literatures, as follows: 12 articles on drug use, 7 articles on smoking, and 13 articles on the use of contraception.

**Figure 3:**
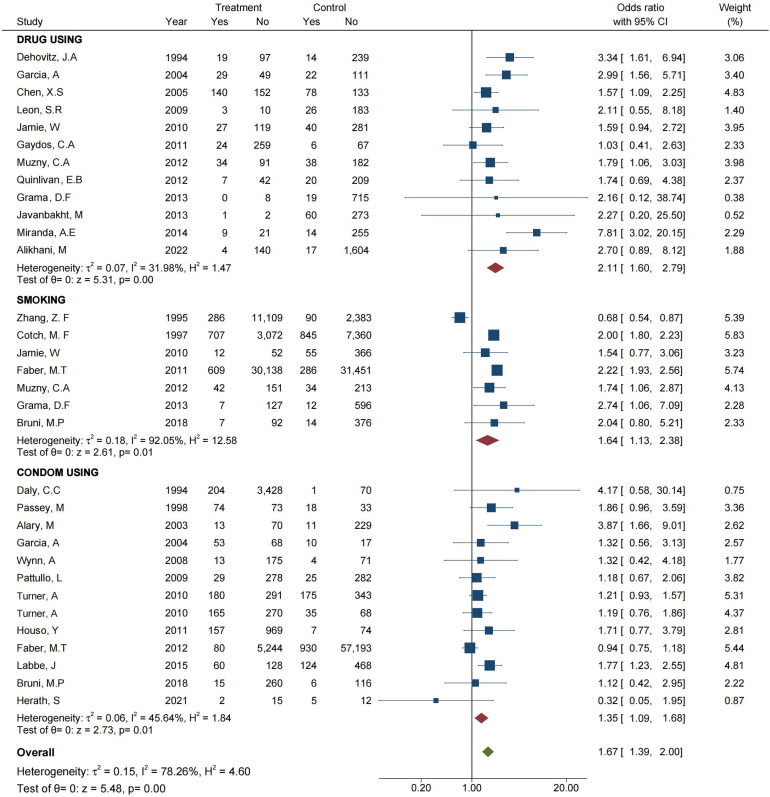
Forest plot of the meta-analysis representing the association between *T. vaginalis* and behavioral variables.

A meta-analysis was conducted with drug users as the experimental group and non-drug users as the control group. As shown in Figure 3, the forest plot demonstrates that there was significant heterogeneity between the drug-taking experimental group and non-drug-taking control group, with *I*^2^ = 31.98% and OR = 2.11 (95% CI: 1.60–2.79), and the pooled effect value was *Z* = 5.31 (*p =* 0.00, *p* < 0.05) [7, 17, 25, 32, 34, 36, 42, 49, 54, 63, 67, 86]. The possibility of smoking as a risk factor for *T. vaginalis* infection was evaluated by meta-analysis with the smokers as the experimental group and non-smokers as the control group, and this result revealed *I*^2^ = 92.05%, OR = 1.64 (95% CI: 1.13–2.38), and the combined effect value of *Z* = 2.61 (*p* = 0.01, *p* < 0.05) [15, 20, 27, 36, 49, 66, 110], which indicated that smoking as a risk factor could promote *T. vaginalis* infection. In addition, we also found that the use of condoms could effectively reduce *T. vaginalis* infection, according to the result of a meta-analysis with *I*^2^ = 45.64%, OR = 1.35 (95% CI: 1.09–1.68), *Z* = 2.73 and *p* = 0.01 (*p* < 0.05) [5, 15, 23, 27, 31, 39, 41, 81, 82, 97, 106].

The combined meta-analysis of the three factors showed a pooled OR = 1.67 (95% CI: 1.39–2.00), *Z* = 5.48 and *p* = 0.00 (*p* < 0.05), which indicated a statistically significant difference in the prevalence of *T. vaginalis* infection between the experimental and control groups (*p* < 0.05). These findings implied that the three kinds of behavioral variables were risk factors for *T. vaginalis* infection (Fig. 3).

#### Correlation between *T. vaginalis* infection and other STIs

In this study, based on the inclusion and exclusion criteria, we collected 19 articles related to the prevalence of *T. vaginalis* and other sexually transmitted infections (STIs). Of these 19 articles, 5 focused on the prevalence of *T. vaginalis* in individuals infected with *Chlamydia*, 9 related to the prevalence of *T. vaginalis* in individuals infected with HIV, and 5 involved the prevalence of *T. vaginalis* in individuals infected with HSV.

**Figure 4:**
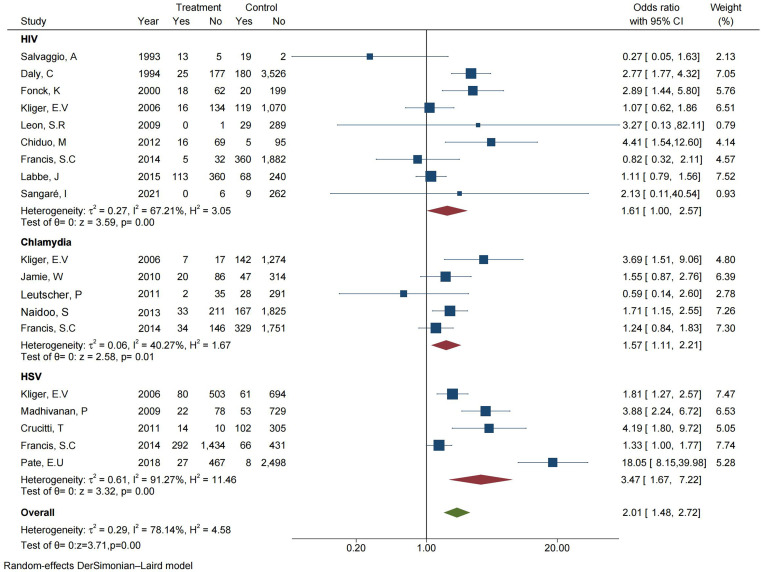
Forest plot of the meta-analysis representing the association between *T. vaginalis* and other STIs.

As shown in Figure 4, comparing the prevalence of *T. vaginalis* between individuals with *Chlamydia* infection and without *Chlamydia* infection through meta-analysis, the forest plot showed *I*^2^ = 40.27%, OR = 1.57 (95% CI: 1.11–2.21), and *Z* and *p-*values of the combined effect size were 2.58 and 0.01 (*p* < 0.05) [30, 34, 50, 51, 68]. A total of 9,366 subjects were included in the 9 articles, including 992 cases with HIV infection and 8,374 cases without HIV infection, and *I*^*2*^ = 67.21%, OR = 1.61 (95% CI: 1.0–2.57), *Z* = 3.59 and *p* = 0.00 [18, 23, 29, 30, 50, 54, 82, 89, 90]. The result showed that *I*^*2*^ = 91.27%, OR = 3.32 (95% CI: 1.67 – 7.22), *Z* = 3.32 and *p* = 0.00 (*p* < 0.05) [21, 22, 30, 50, 80], comparing the prevalence of *T. vaginalis* between individuals with HSV infection and without HSV infection.

The populations with *Chlamydia*, HSV, or HIV infection were combined into the other STI group. The results showed that 24,053 subjects were included in the articles, including 4,564 cases with STIs and 19,489 cases without STIs. The pooled OR was 2.01 (95% CI: 1.48–2.72), and the combined effect were *Z* = 3.71 and *p* = 0.00 (*p* < 0.05), which meant that there was a significant difference in the with other STIs group and without other STIs group (*p* < 0.05).

#### Correlation of *T. vaginalis* prevalence with socioeconomic variables

The present study included 41 articles on the effect of socioeconomic variables on the transmission of *T. vaginalis.* The investigations encompassed three main aspects: marital status (21 articles), income level (11 articles), and work status (9 articles).

**Figure 5:**
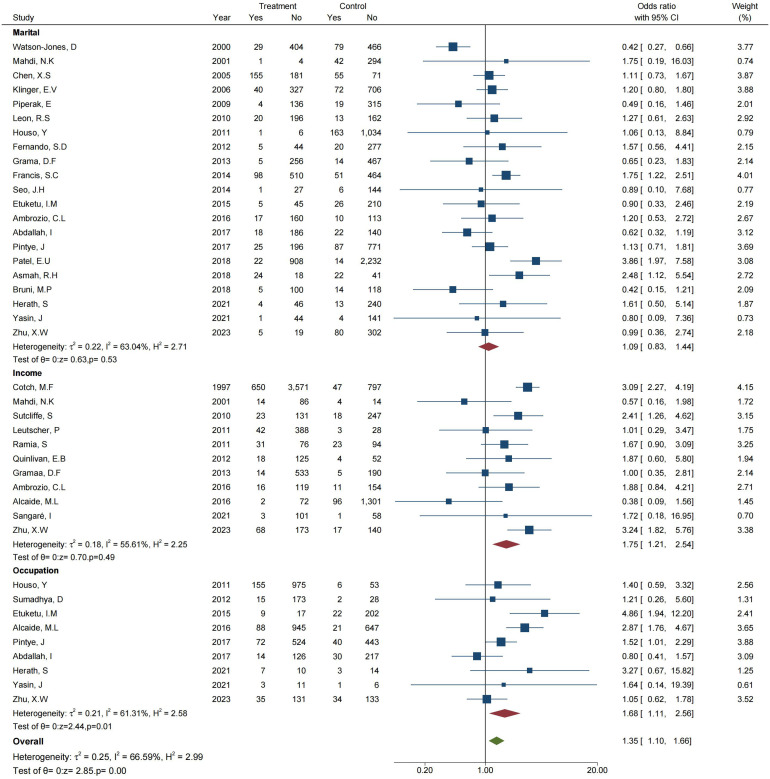
Forest plot of the meta-analysis representing the association between *T. vaginalis* prevalence and socioeconomic variables.

A meta-analysis was conducted with the working population as the experimental group and the non-working population as the control group. As shown in Figure 5, *I*^2^ = 61.31%, OR = 1.68 (95% CI: 1.11–2.56), the combined effect *Z* = 2.44, *p* = 0.01 (*p* < 0.05) [78, 79, 93–99] suggests that working is a risk factor for *T. vaginalis* infection [1, 6, 26, 28, 39, 41, 83, 107, 111].

The results of the impact of marital status and income level on the prevalence of *T. vaginalis* showed that the pooled OR was 1.75 (95% CI: 1.21–2.54), *I*^2^ = 55.61%, *Z* = 0.70 and *p* = 0.49 (*p* > 0.05) in the analysis of marital factors, and OR = 1.09 (95% CI: 0.83–1.44), *I*^*2*^ = 63.04%, the combined effect *Z* = 0.63 and *p* = 0.53 (*p* > 0.05) in the analysis of income factors. Although the *p-*value was greater than 0.05, the pooled OR values were 1.75 and 1.09 in the analysis of marital and income factors, respectively, which indicates that marital status and income level might be risk factors for *T. vaginalis* infection [1, 6, 8, 9, 15, 17, 20, 26, 28, 30, 36, 39, 41, 50, 54, 58, 80, 84, 86, 87, 90, 91, 95, 103, 104, 107, 111].

Additionally, the combined meta-analysis of the three kinds of socioeconomic variables showed that OR = 1.35 (95% CI: 1.10–1.66), *I*^*2*^ = 66.59%, *Z* = 2.85 and *p* = 0.00 (*p* < 0.05). The findings proved that socio–economic variables played a role in influencing *T. vaginalis* infection (Fig. 5).

## Discussion

*Trichomonas vaginalis* is a widely spread sexually transmitted pathogen that poses a serious threat to the health of the human urinary and reproductive system. However, few studies have systematically reviewed the global prevalence of *T. vaginalis.* In order to summarize the global prevalence of *T. vaginalis* and explore the potential risk factors for *T. vaginalis* infection, we collated and analyzed literature articles published between 1992 and 2023 on the prevalence of *T. vaginalis.* After rigorous inclusion and exclusion criteria were applied, 425 high-quality articles were screened, encompassing data on the prevalence of *T. vaginalis* in 85 countries worldwide. Our research aims to provide valuable references for the development of global public health strategies.

This work provides the first comprehensive global analysis of *T. vaginalis* infection, revealing valuable insights into its prevalence and distribution worldwide. By consolidating data from multiple countries and regions, we found that the global average prevalence of *T. vaginalis* was 8% (95% CI: 7%–10%), a result consistent with previous studies [88]. Harfouche et al indicated that the prevalence of *T. vaginalis* among the general population in the Middle East and North Africa was 4.7% [37], while our statistical analysis results for the Middle East region and South Africa were 10% (95% CI: 0%–20%). The possible reason for the difference is that our summary results are based on all the populations included in the literature, including high-risk groups. In addition, we analyzed the prevalence rates in different countries in the Middle East and South Africa, and the results are as follows: 7% (95% CI: 0%–14%) in Iran, 35% (95% CI: 8%–62%) in Iraq, 3% (95% CI: 2%–8%) in Israel, 20% (95% CI: 5%–36%) in Egypt, 6% (95% CI: 5%–18%) in Sudan, and 2% (95% CI: 0%–3%) in Türkiye. Although *T. vaginalis* infection was widespread, the prevalence varied significantly across different countries and regions, ranging from 1% to 35%. This variation suggests that the occurrence of *T. vaginalis* infection may be influenced by a complex interplay of risk factors, including socioeconomic conditions, cultural differences, and behavioral variables.

*Trichomonas vaginalis* is a parasite primarily transmitted through sexual contact. Previous research has established that commercial sex workers have significantly higher infection rates compared to other populations [16, 93]. Our study observed that in some countries, the infection rate exceeded 20%, particularly among female commercial sex workers, which may account for the higher prevalence in these regions. However, due to the lack of specific focus on commercial sex workers in some studies, our meta-analysis revealed substantial heterogeneity (*I*^2^ > 95%) in infection rates across countries, indicating that additional risk factors may contributed to this variability. Thus, further investigation into these factors is essential for uncovering new transmission pathways for *T. vaginalis*.

Healthy behavioral habits are crucial in reducing the risk of *T. vaginalis* transmission and infection OR = 1.67 (95% CI: 1.39–2.0). Our findings indicate that substance abuse and smoking are associated with an increased risk of *T. vaginalis* infection. Although current evidence does not establish a direct link between smoking and sexually transmitted infections, some researchers suggest that excessive smoking may impair the immune system, increasing susceptibility to various infections, including sexually transmitted pathogens. In addition, the transmission of *T. vaginalis* can be effectively blocked through appropriate contraception, which is consistent with previous research and further underscores the importance of healthy behavior habits in preventing sexually transmitted diseases [85].

Infections with other sexually transmitted pathogens also are considered to be related to *T. vaginalis* infection, OR = 2.01 (95% CI: 1.48–2.72). Our study found that infections with *Chlamydia*, HIV, and HSV may elevate the risk of *T. vaginalis* infection, aligning with the research on female sex workers in Kenya. *Chlamydia* and HSV can secrete adhesins or upregulate glycoproteins on host cell surfaces, thereby facilitating the adhesion of *T. vaginalis* to host cells, promoting its infection. Additionally, previous research has found that *T. vaginalis* is positively associated with HPV infection, and HPV is also a risk factor for *T. vaginalis* infection [19, 59, 61, 69].

Our research highlights a significant relationship between economic status and *T. vaginalis* infection, risk OR = 1.35 (95% CI: 1.10–1.66). The prevalence of *T. vaginalis* is generally higher in low-income countries. The World Health Organization has noted that women in low-income settings experience a greater burden of *T. vaginalis* infections. Low income is often linked to lower education levels, which may contribute to inadequate knowledge of safe sexual practices [17]. Additionally, low-income individuals may face challenges in accessing essential healthcare services, further increasing infection risk. Our study also found that individuals without stable employment are at higher risk of *T. vaginalis* infection, likely due to lower economic status and reduced access to healthcare. Conversely, stable marriages, often associated with economic stability, may help reduce infection risk. However, individuals in stable marriages who do not use effective contraceptive measures may also contribute to the spread of *T. vaginalis.*

In the initial screening process, we found some factors that might cause the spread of *T. vaginalis*, including religious beliefs, sexual orientation, cervical intraepithelial neoplasia (CIN), the number of CD4 cells, the number of female pregnancies, male circumcision, the use of female intrauterine devices and hormone contraceptives, sexual practices, menstrual cycle, and the types of latrines [3, 22, 24, 90].

Through the analysis of seven methods for detecting *T. vaginalis*, it was found that there were significant differences in the pooled prevalences of *T. vaginalis* with the different methods (range from 6% to 13%), among which PCR and culture method showed a higher detection rate. The results in this study are consistent with the previous literature [58, 59].

Despite comprehensive efforts to compile data on *T. vaginalis* prevalence, our analysis of global trends and associated risk factors remains constrained by several limitations. There is a lack of clear population classification, difficulty in effectively distinguishing between incidental and persistent *T. vaginalis* infections, and limitations in data capacity. Therefore, more attention needs to be paid to studying the prevalence of *T. vaginalis* and the risk factors for parasitic infections. Additionally, it is recommended to adopt more scientific detection methods to improve the accuracy and reliability of the data.

## Conclusion

*Trichomonas vaginalis* is a serious sexually transmitted pathogen that deserves close attention. In this work, we provided information about the prevalence of *T. vaginalis* in different countries around the world in terms of space, time, and population distribution, by summarizing and analyzing published epidemiological articles on *T. vaginalis.* Moreover, we discovered that certain behaviors including smoking, drug use, and not using condoms, infection with other sexually transmitted diseases, and low income are potential risk factors for *T. vaginalis* infection*.* This research advances our understanding of the global prevalence of *T. vaginalis* and provides new insights into interrupting *T. vaginalis* transmission.

## Data Availability

All resources used in this article are provided in the Supporting Information and all the analyses are detailed allowing the assessment or verification of the manuscript’s findings.

## Supplementary Materials

The supplementary material for this article can be found at https://www.parasite-journal.org/10.1051/parasite/2025051/olm. Supplementary file supplied by the authors.
