# Supplementary material for: Systematic review and meta-analysis of the global prevalence and infection risk factors of Trichomonas vaginalis
Source: Parasite. 2025 Aug 27;32:56. doi: 10.1051/parasite/2025051 (PMC12386857; doi:10.1051/parasite/2025051)
Supplement: Supplementary file 1 — Supplementary file supplied by the authors. [file parasite-32-56-s1.zip › parasite240166-1-olm/Figure S1.docx]

(A)


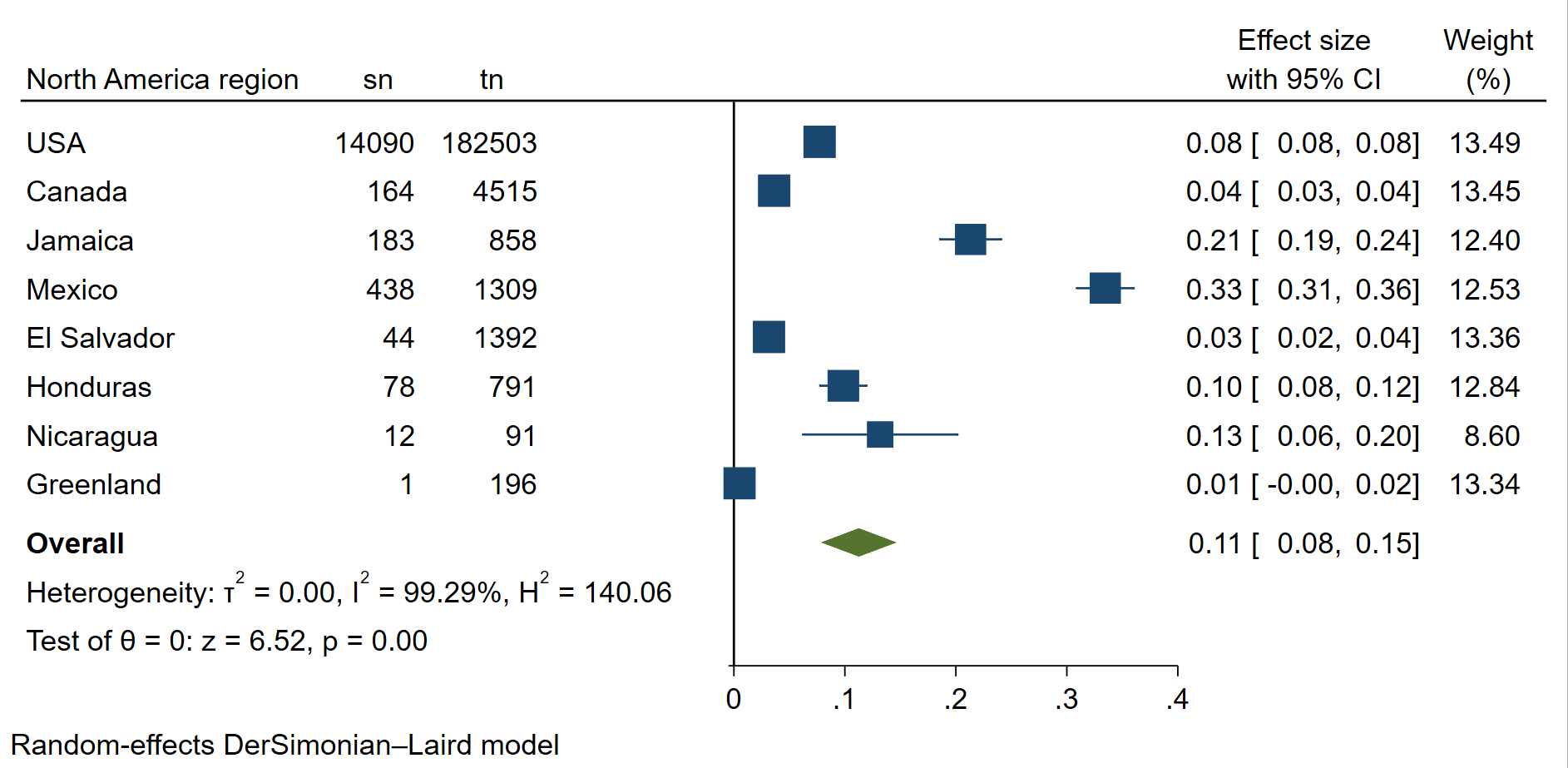


(B)

**
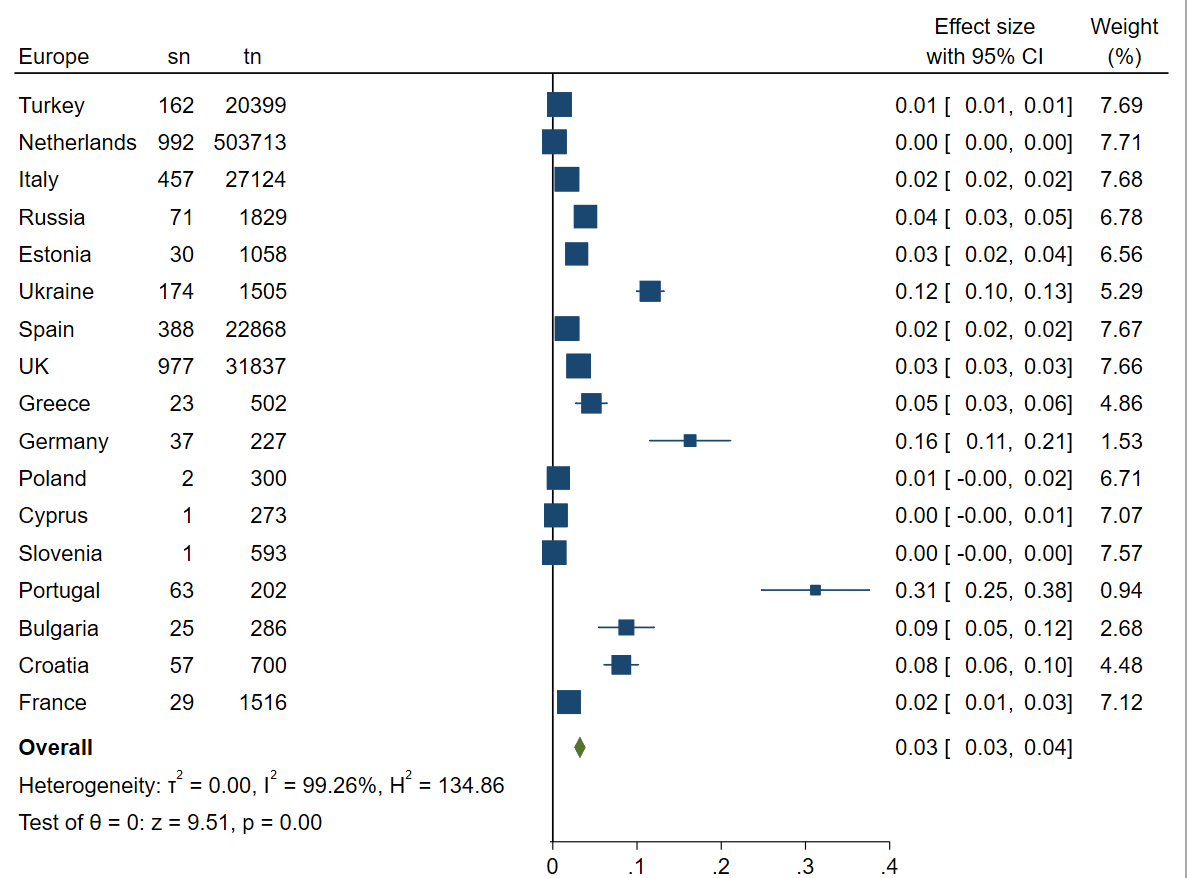
**


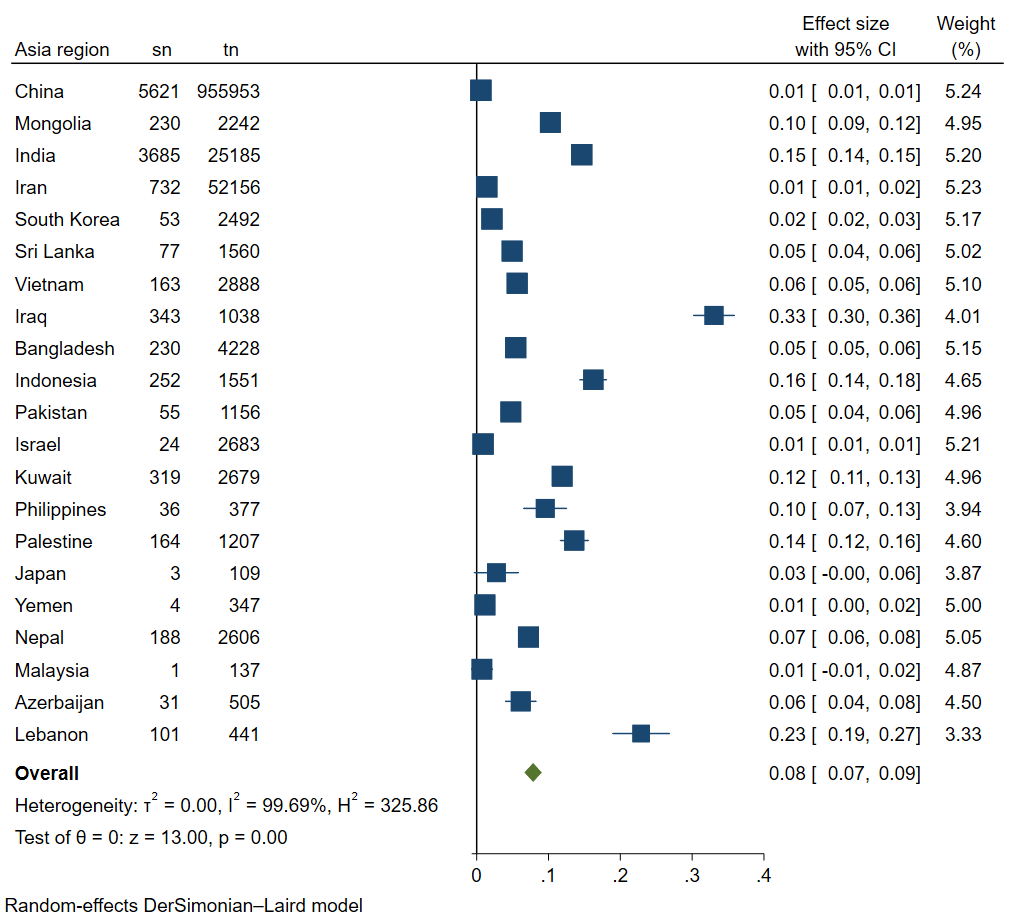
(C)

(D)


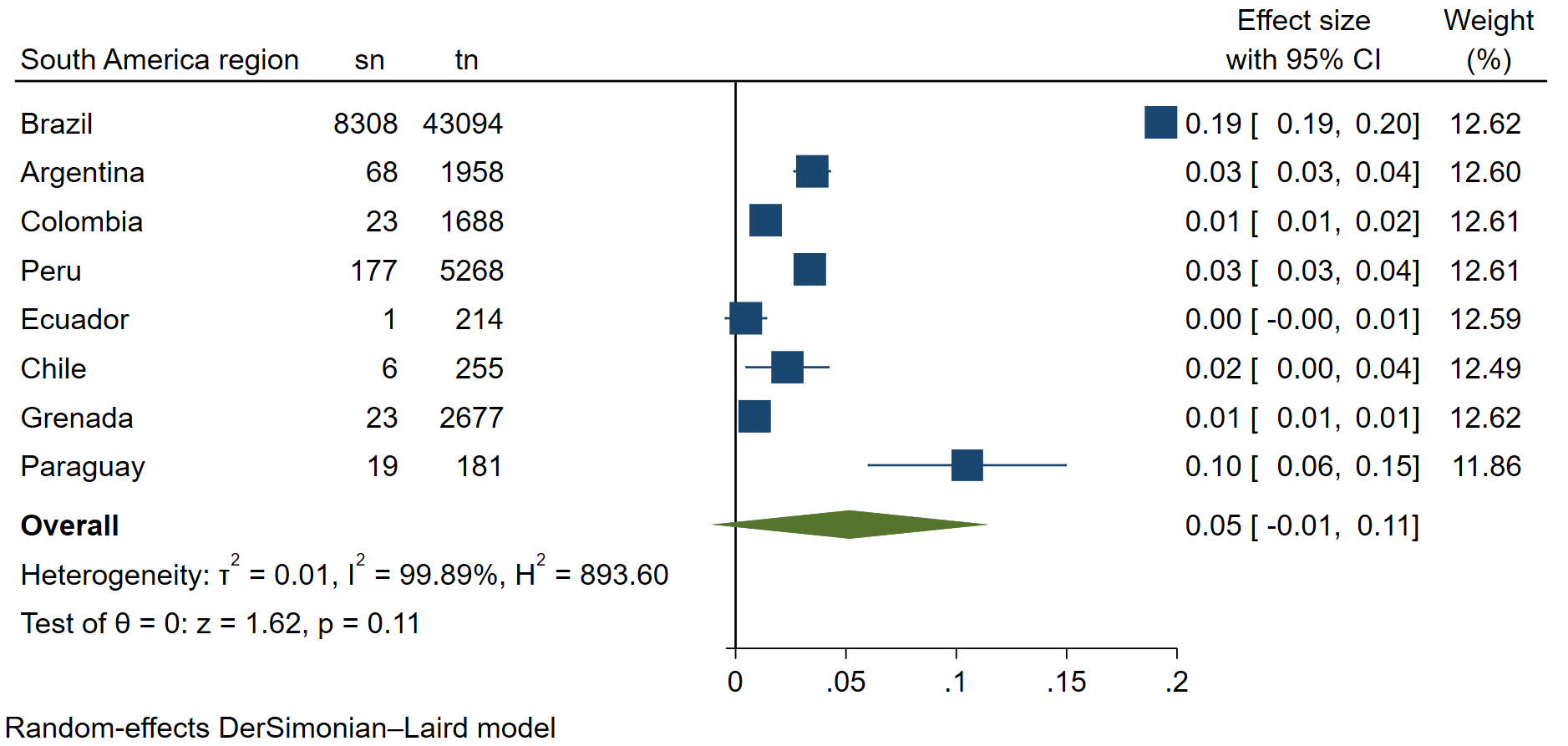


(E)


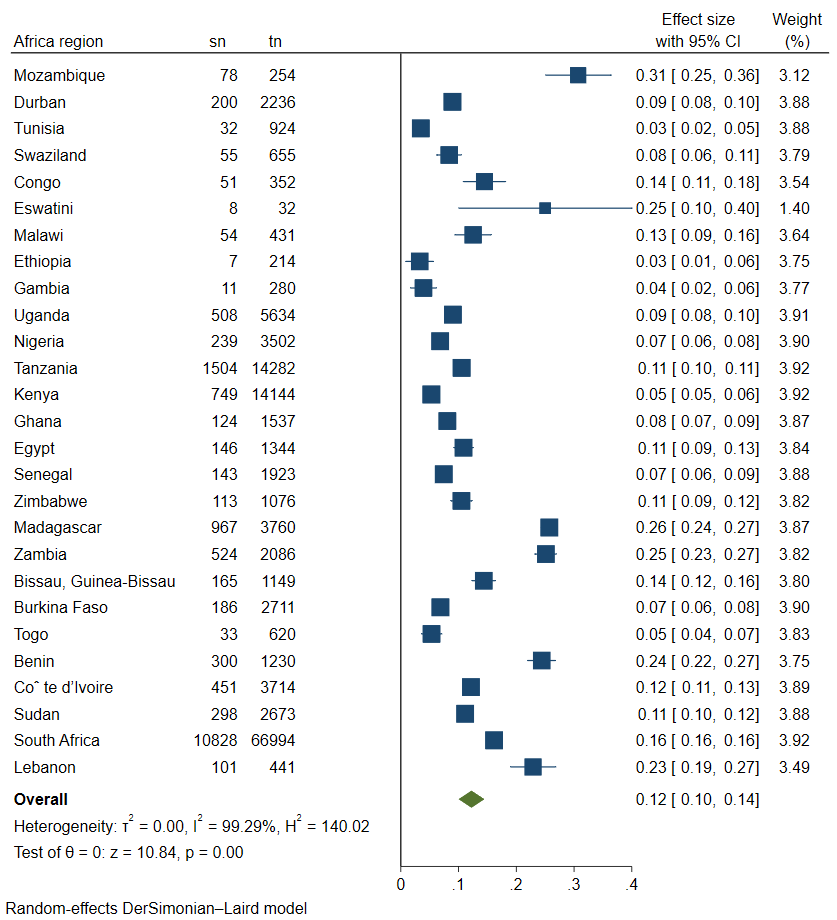


(F)


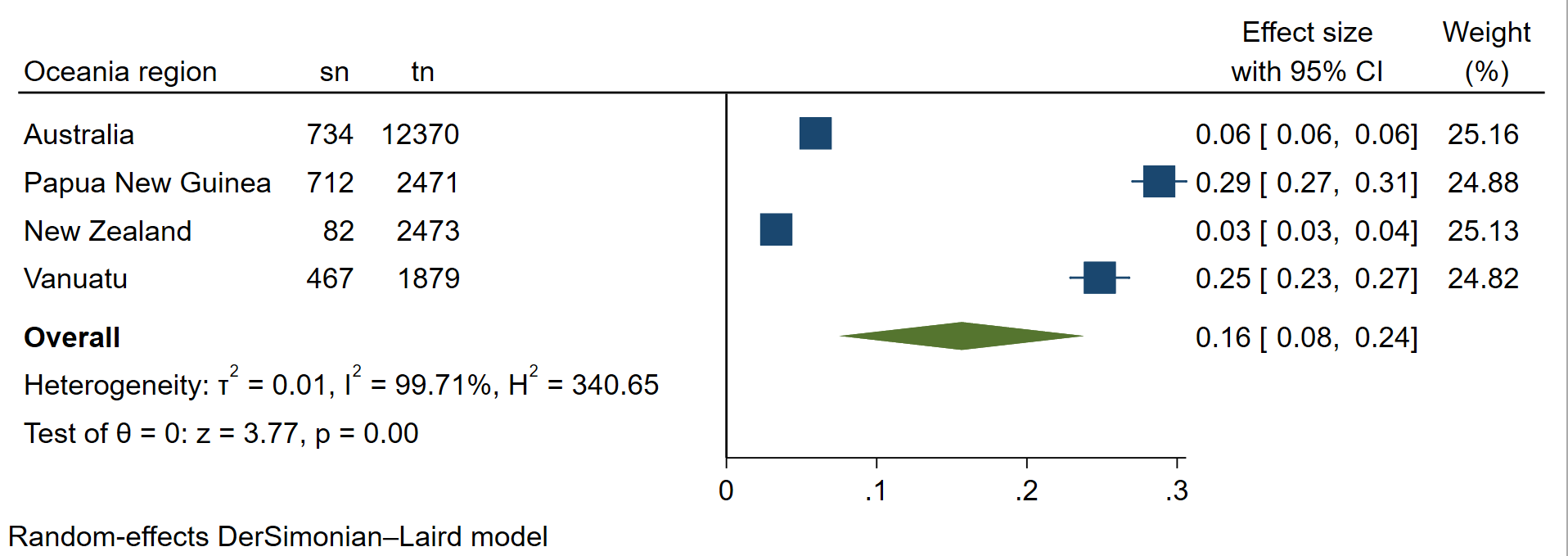


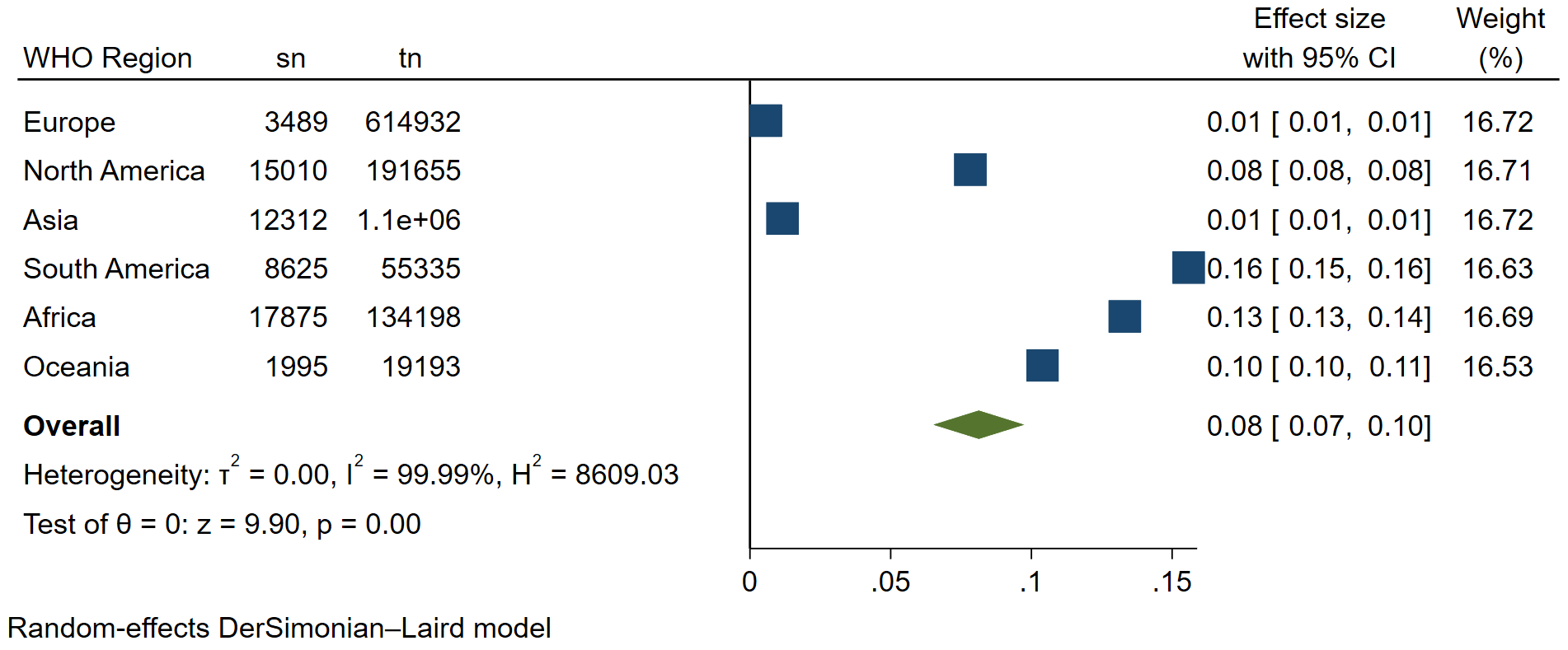
(G)

**Figure S1:**The forest plots map representing the global prevalence of *T. vaginalis* in different countries based on included studies.

(A)North America region; (B)Europe; (C)Asia region (D)South America region; (E)Africa region; (F)Oceania region; (G)WHO Region.
