# Supplementary material for: Systematic review and meta-analysis of the global prevalence and infection risk factors of Trichomonas vaginalis
Source: Parasite. 2025 Aug 27;32:56. doi: 10.1051/parasite/2025051 (PMC12386857; doi:10.1051/parasite/2025051)
Supplement: Supplementary file 1 — Supplementary file supplied by the authors. [file parasite-32-56-s1.zip › parasite240166-1-olm/Figure S2.docx]

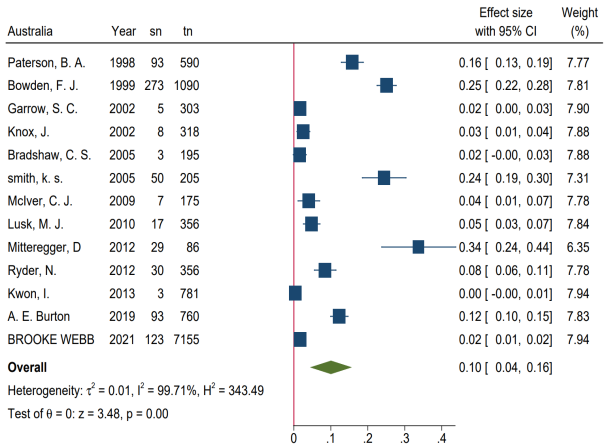
（1）

（2）


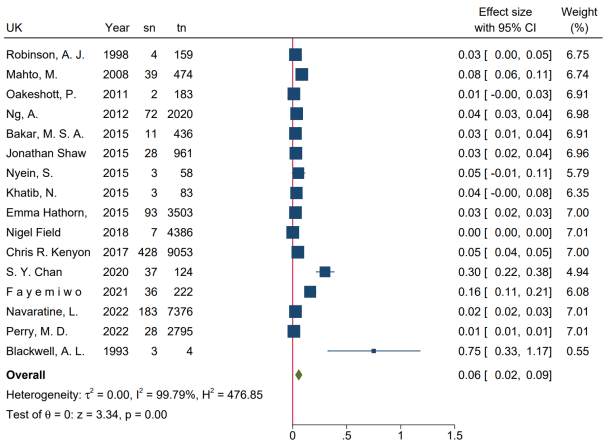


（3）


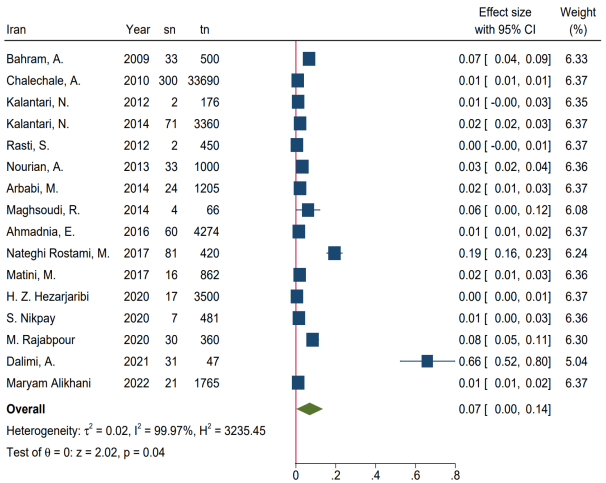


（4）


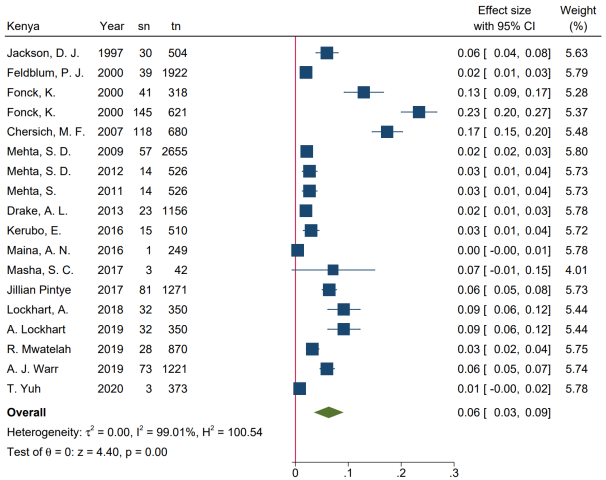


（5）


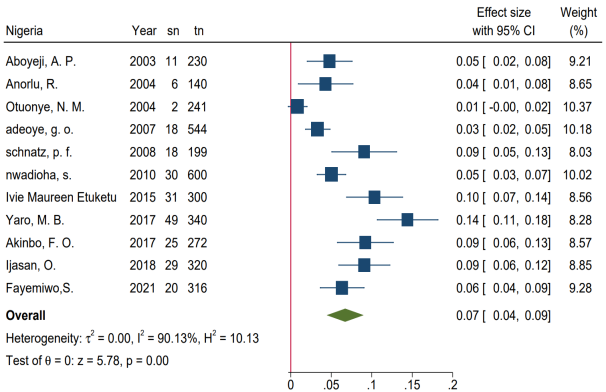


（6）


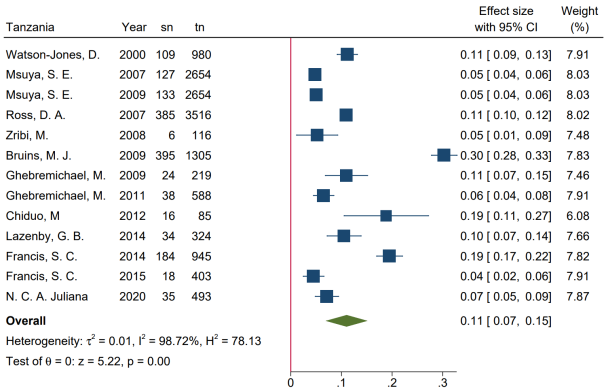


（7）


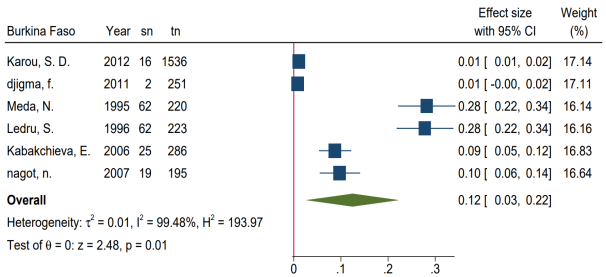


（8）


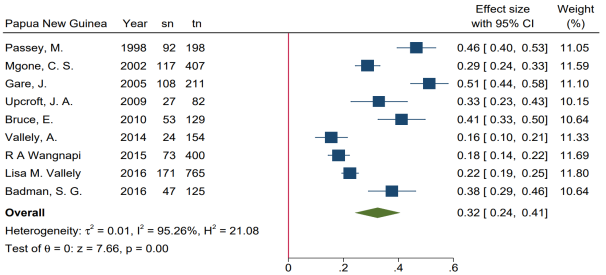


（9）


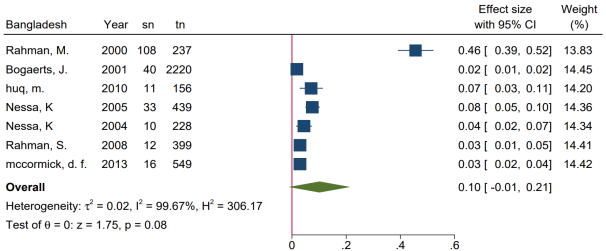


（10）


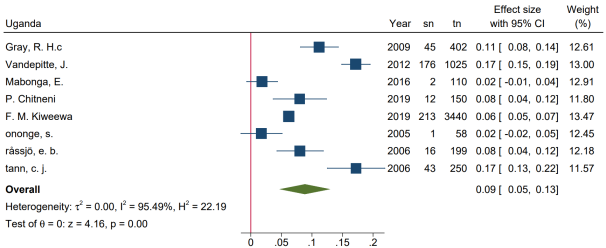


（11）


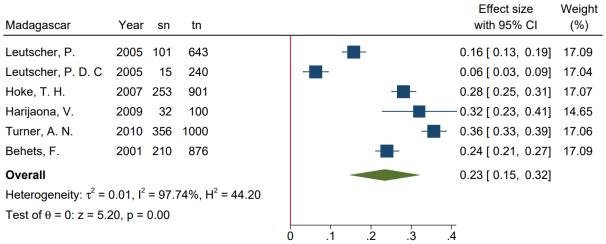


（12）


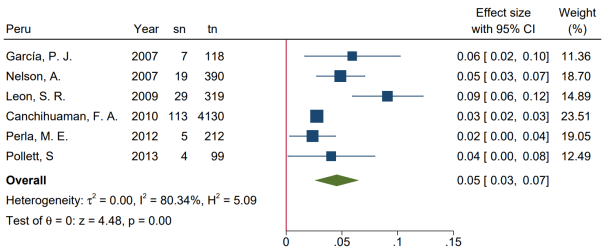


（13）


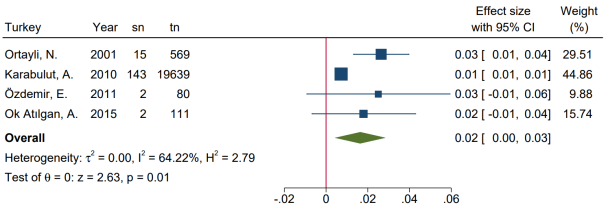


（14）


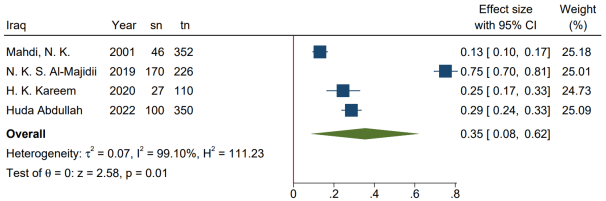


（15）


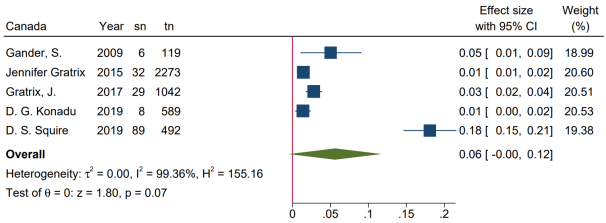


（16）


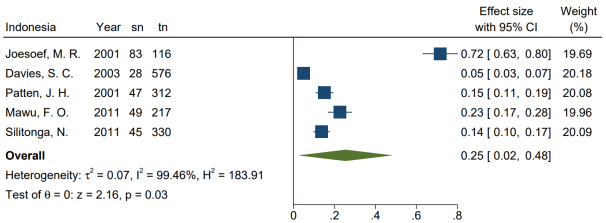


（17）


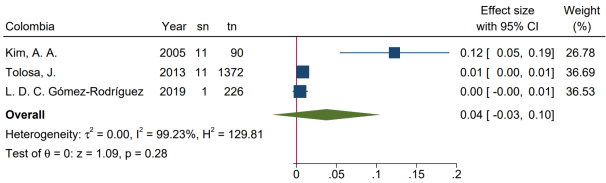


（18）


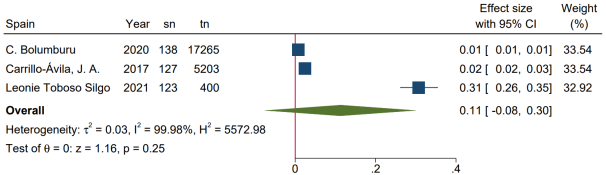


（19）


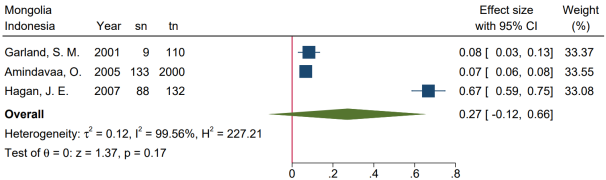


（20）


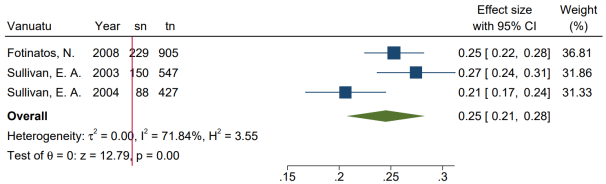


（21）


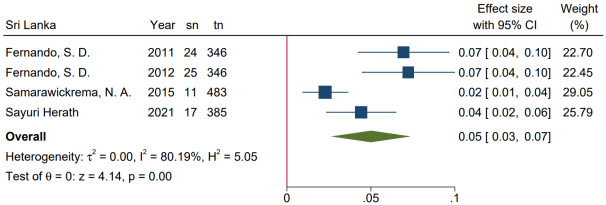


（22）


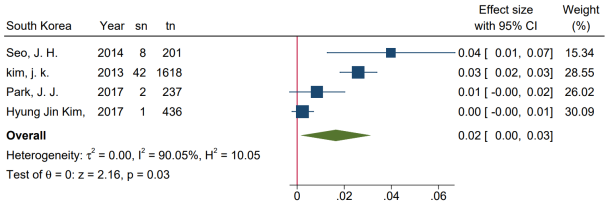


（23）


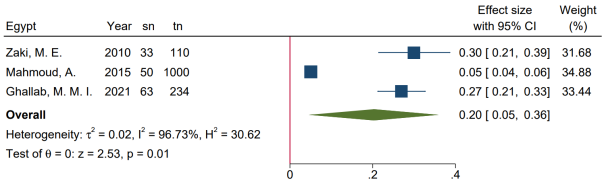


（24）


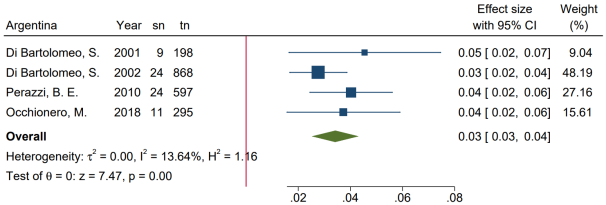


（25）


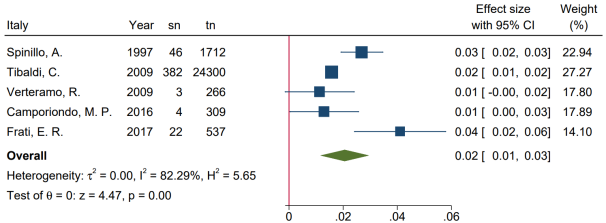


（26）


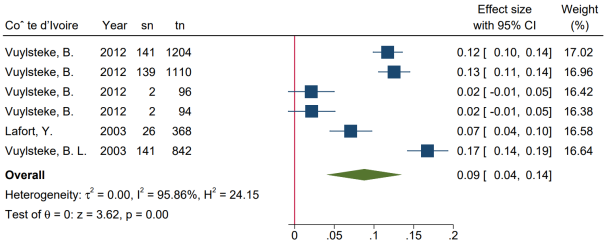


（27）


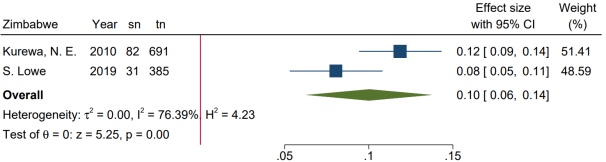


（28）


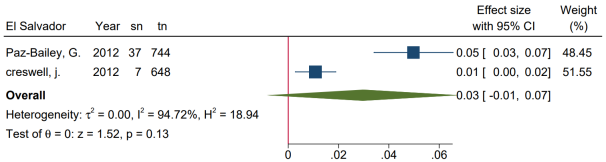


（29）


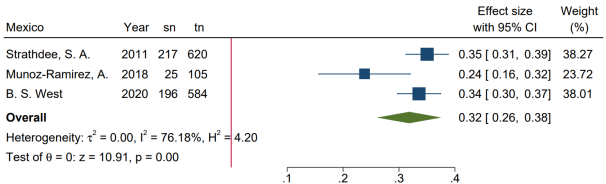


（30）


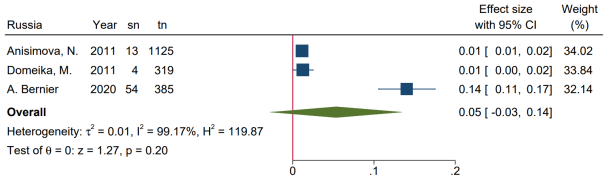


（31）


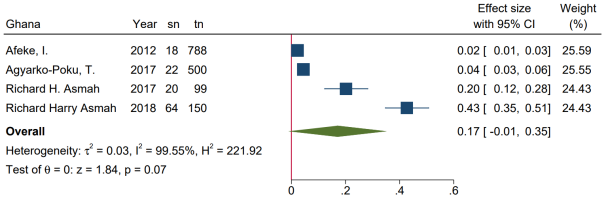


（32）


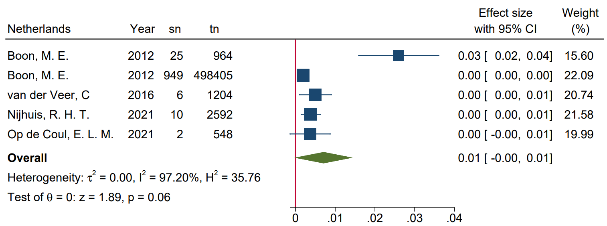


（33）


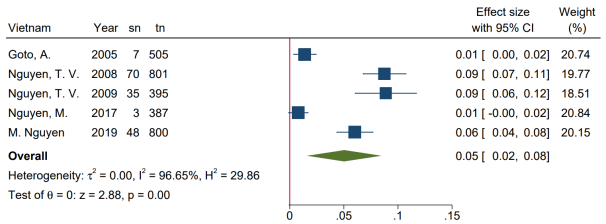


（34）


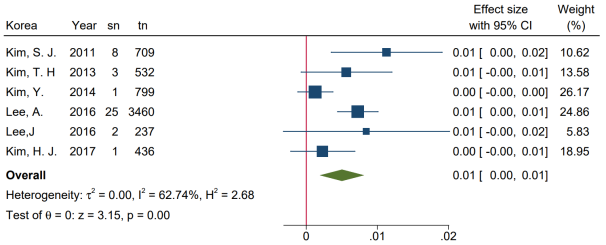


（35）


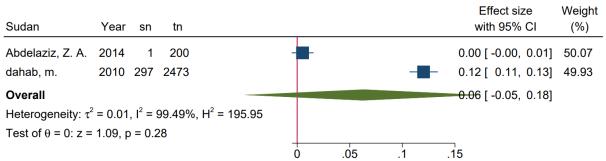


（36）


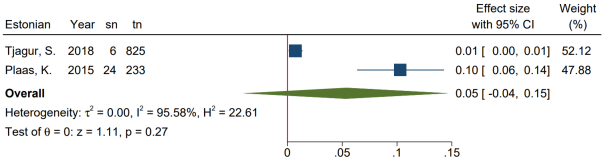


（37）


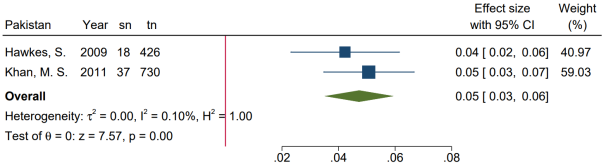


（38）


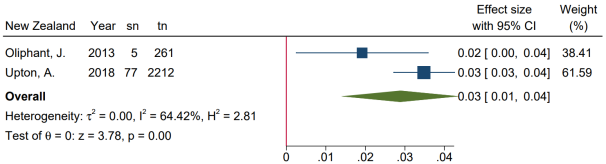


（39）


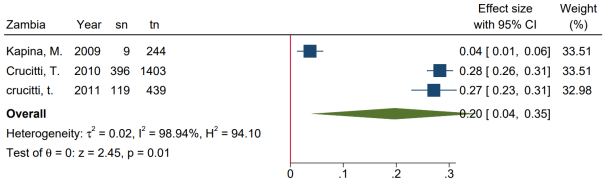


（40）


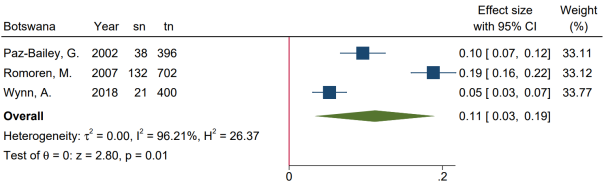


（41）


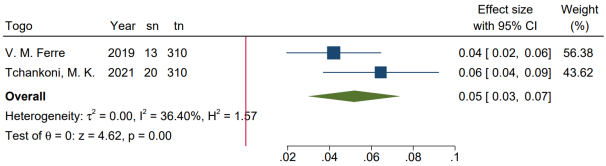


（42）


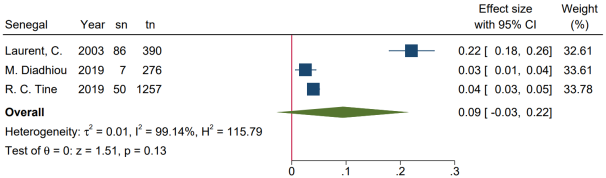


（43）


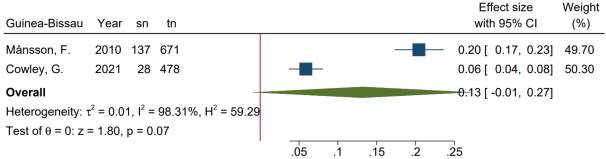


（44）


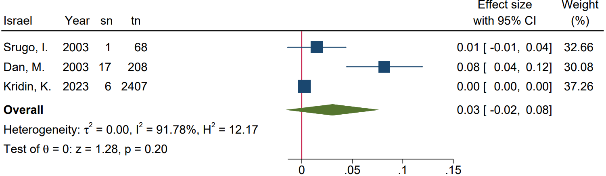


（45）


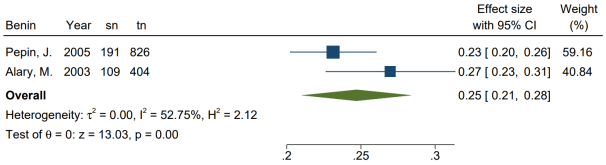


（46）


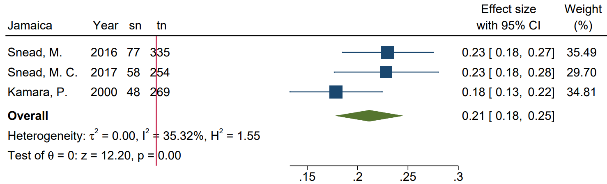


（47）


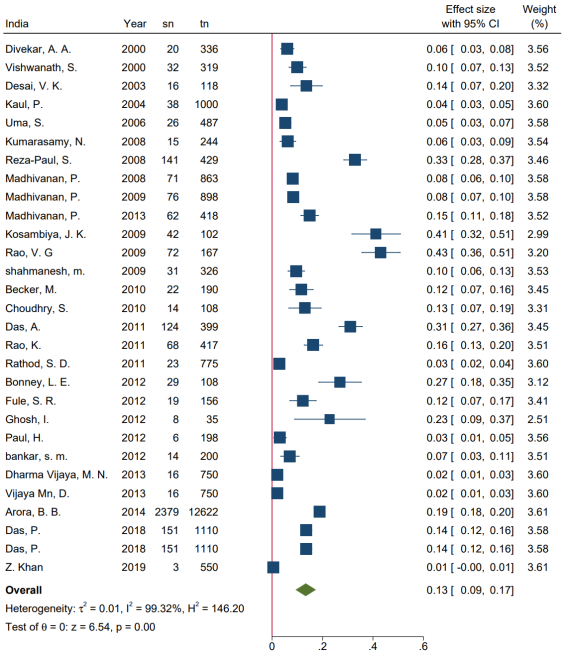


（48）


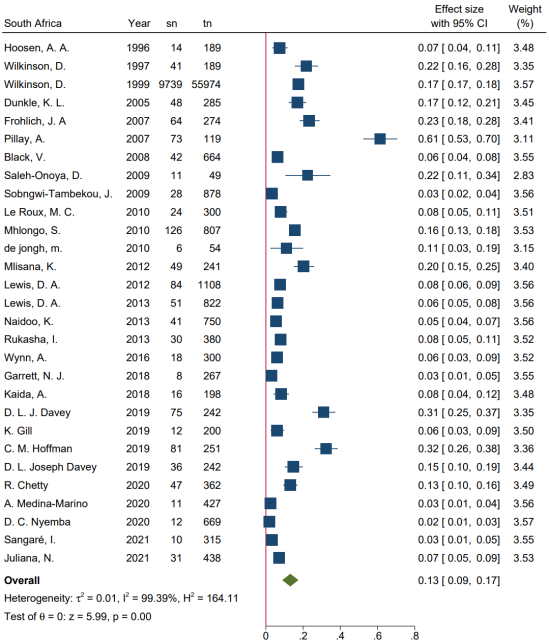


（49）


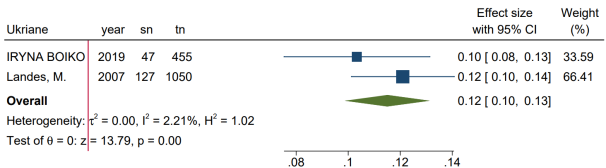


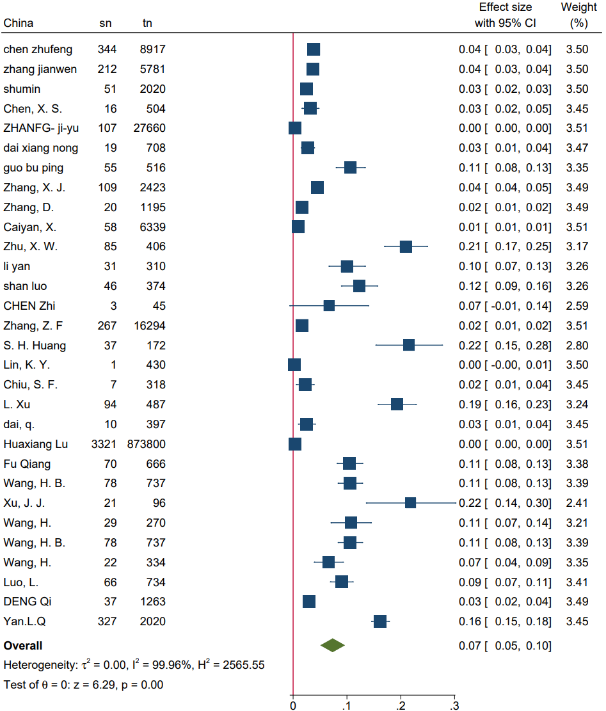
（50）

（51）


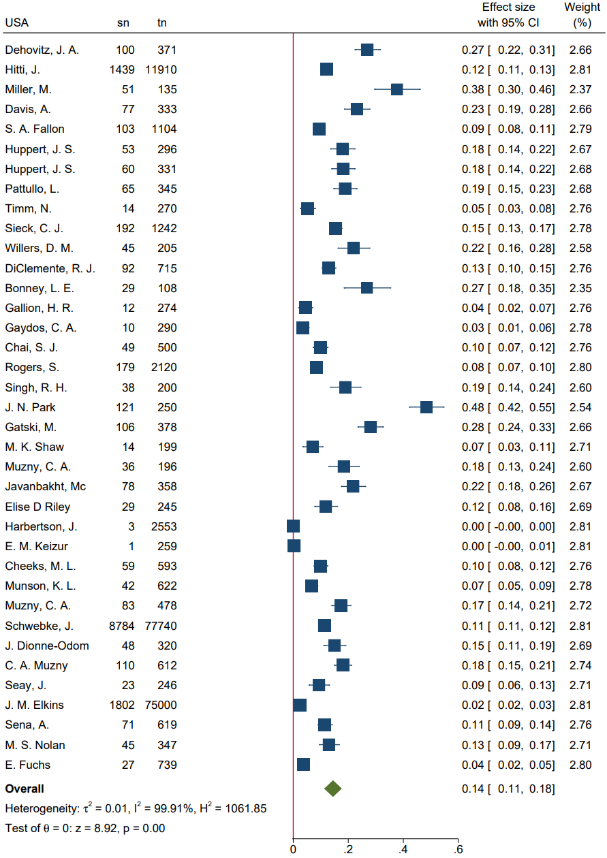


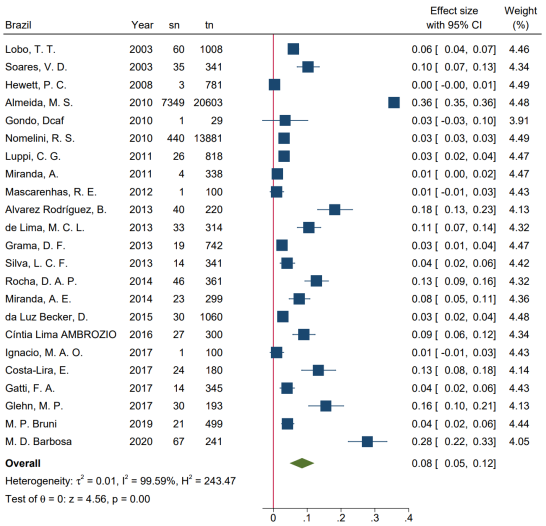
（52）

**Figure S2:**The forest plots map representing the prevalence of *T. vaginalis* in different countries based on included studies.

1. Australia; (2) UK; (3) Iran; (4) Kenya; (5) Nigeria; (6) Tanzania; (7) Burkina Faso; (8) Papua New Guinea; (9) Bangladesh; (10) Uganda; (11) Madagascar; (12) Peru; (13) Turkey; (14) Iraq; (15) Canada; (16) Indonesia; (17) Colombia; (18) Spain; (19) Mongolia; (20) Vanuatu; (21) Sri Lanka; (22) South Korea; (23) Egypt; (24) Argentina; (25) Italy; (26) Coˆ te d’Ivoire; (27 )Zimbabwe; (28) El Salvador; (29) Mexico; (30) Russia; (31) Ghana; (32) Netherlands; (33) Vietnam; (34) Korea; (35) Sudan; (36) Estonian; (37) Pakistan; (38) New Zealand; (39) Zambia; (40) Botswana; (41 )Togo; (42) Senegal; (43) Guinea-Bissau; (44) Israel; (45) Benin; (46) Jamaica; (47) India; (48) South Africa; (49) Ukraine; (50) China; (51) America; (52) Brazil.
