# Supplementary material for: Systematic review and meta-analysis of the global prevalence and infection risk factors of Trichomonas vaginalis
Source: Parasite. 2025 Aug 27;32:56. doi: 10.1051/parasite/2025051 (PMC12386857; doi:10.1051/parasite/2025051)
Supplement: Supplementary file 1 — Supplementary file supplied by the authors. [file parasite-32-56-s1.zip › parasite240166-1-olm/Figure S3.docx]

(A)


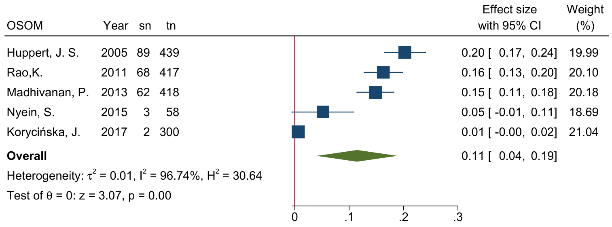


(B)


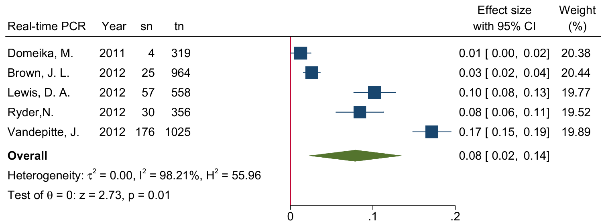


(C)


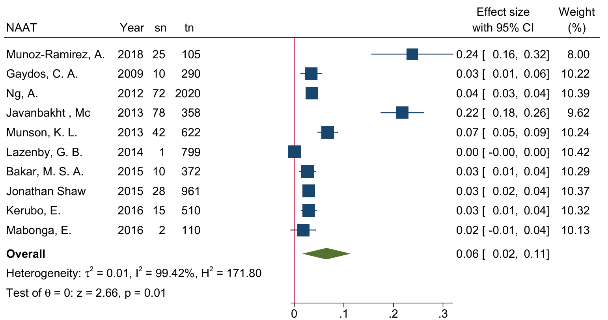


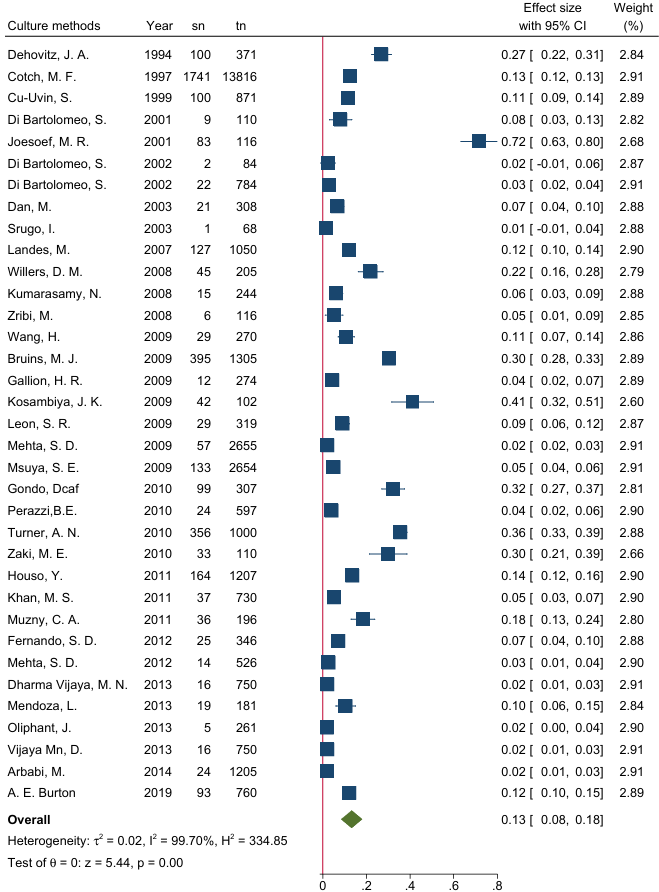
(D)


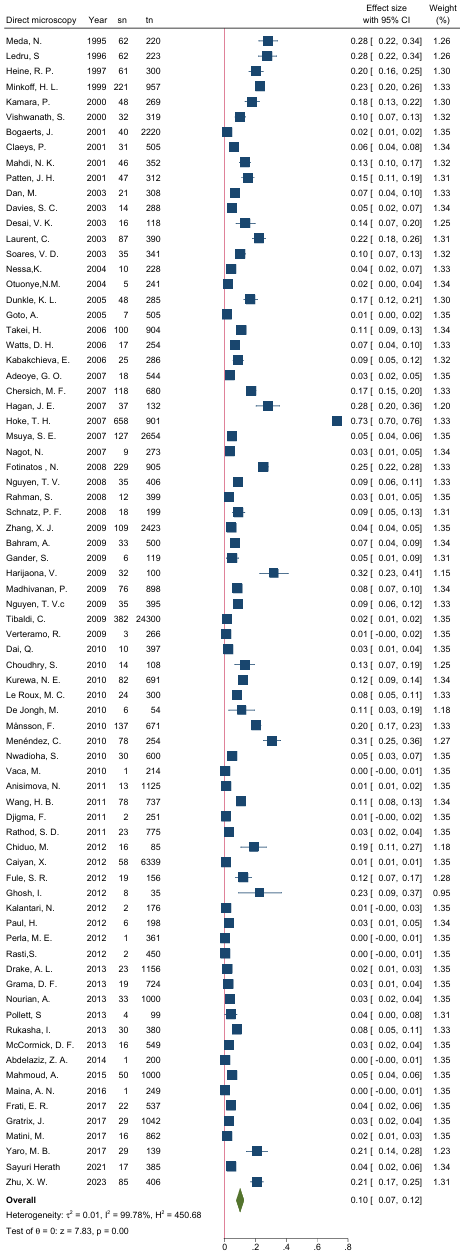


(F)


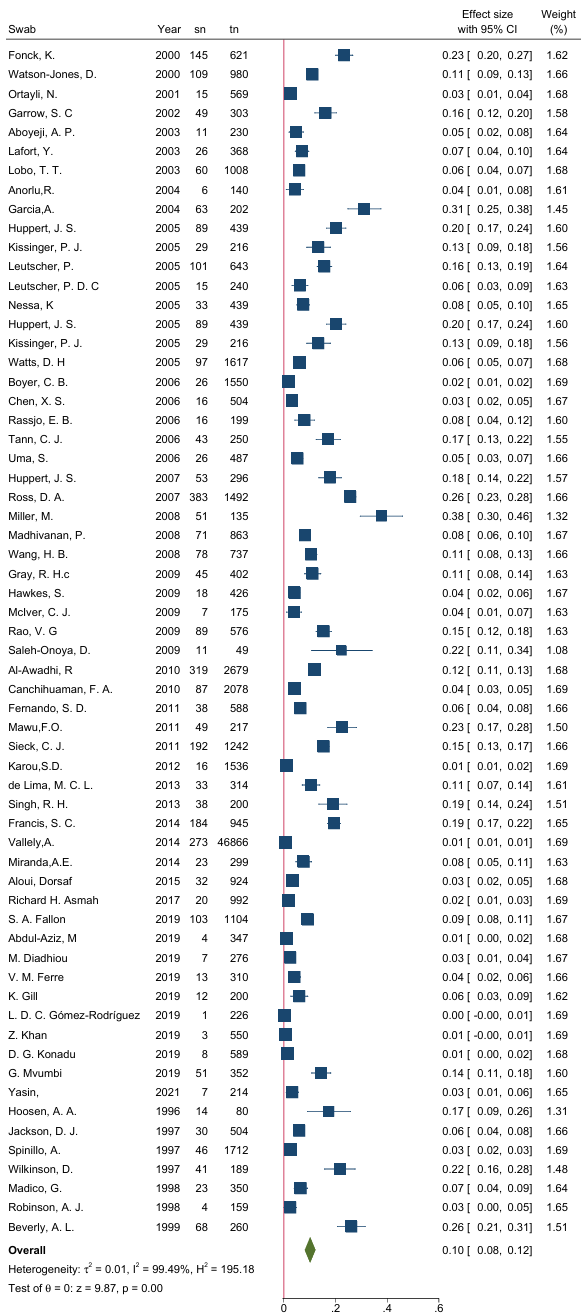


(G)


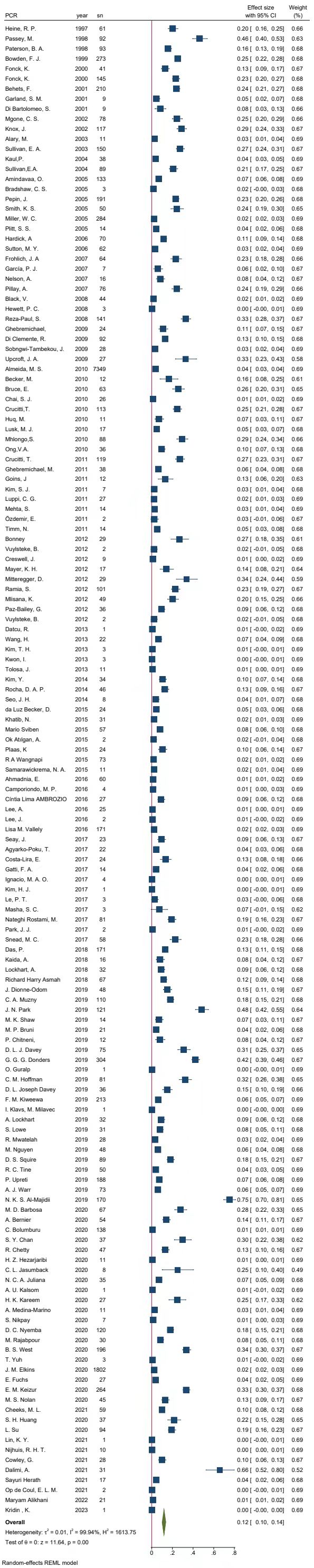


(G)


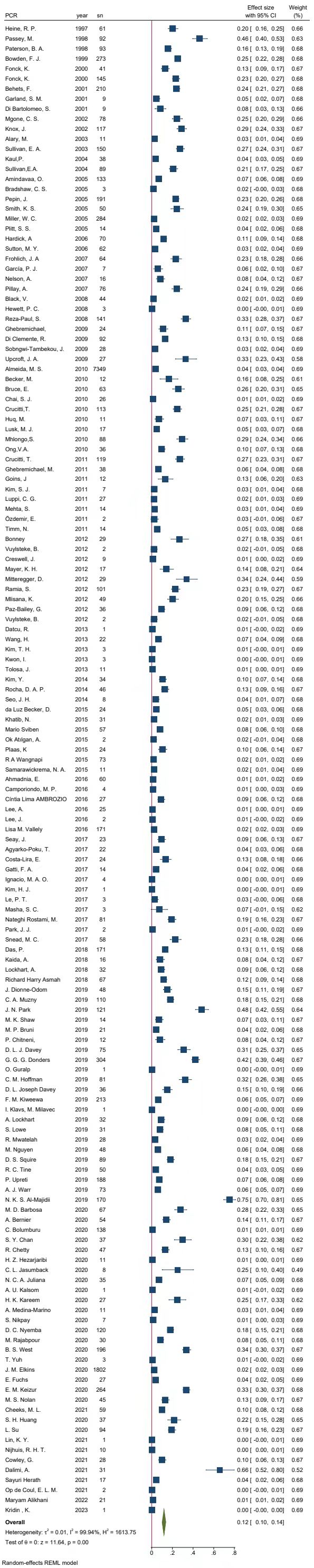


(G)


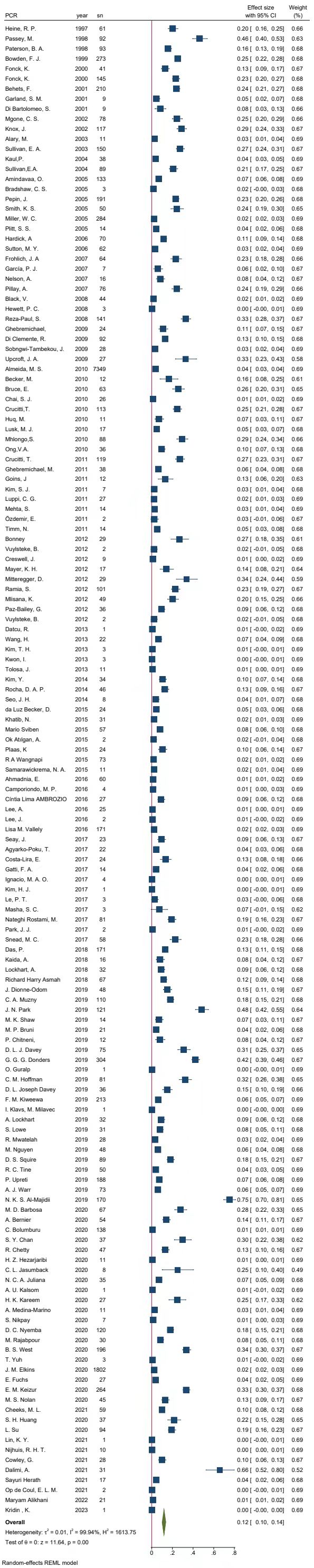
 **Figure S3 :** Forest plot analysis of *T. vaginalis* prevalence based on different detection methods.

(A) OSOM detection method; (B) Real time PCR detection method; (C) NAAT detection method;

(D) Culture method;(E) Swab detection method; (F) Direct microscopy method; (G) PCR detection

Method.
