# Supplementary material for: Systematic review and meta-analysis of the global prevalence and infection risk factors of Trichomonas vaginalis
Source: Parasite. 2025 Aug 27;32:56. doi: 10.1051/parasite/2025051 (PMC12386857; doi:10.1051/parasite/2025051)
Supplement: Supplementary file 1 — Supplementary file supplied by the authors. [file parasite-32-56-s1.zip › parasite240166-1-olm/Table S1.docx]

**Table S1.** Systematic review of 425 studies reporting the prevalence of *T. vaginalis* infection.

| **Author** | **References** | **Year** | **Country/Region** | **Diagnostic**  **Method** | **Populations** | **Sex** | **Total**  **Individuals** | **Positive**  **Cases** | **Prevalence**  **(%)** |
| --- | --- | --- | --- | --- | --- | --- | --- | --- | --- |
| **Blackwell, A. L.** | [38] | 1993 | UK | ND | ND | ND | 4 | 3 | 75% |
| **Dehovitz, J. A.** | [91] | 1994 | USA | Culture methods | Women | Female | 371 | 100 | 26.95% |
| **Meda, N.** | [250] | 1995 | Burkina Faso | Direct microscopy | Non-pregnant Women | Female | 220 | 62 | 28% |
| **Ledru, S.** | [217] | 1996 | Burkina Faso | Direct microscopy (on saline mount) | Patients | Female | 223 | 62 | 27.8% |
| **Wang, P. D.** | [403] | 1995 | China | Papanicolaou (Pap) smears biopsy | Women | Female | 17047 | 320 | 1.88% |
| **Zhang, Z. F** | [421] | 1995 | China | Papanicolaou (Pap) smears biopsy | Women | Female | 16797 | 421 | 2.51% |
| **Hoosen, A. A.** | [161] | 1996 | South Africa | Swab | Non-pregnant Women | Female | 80 | 14 | 17.5% |
| **Jackson, D. J.** | [170] | 1997 | Kenya | Swab | Men | Male | 504 | 30 | 6% |
| **Spinillo, A.** | [364] | 1997 | Italy | Swab | Patients | Female | 1712 | 46 | 2.69% |
| **Wilkinson, D.** | [411] | 1997 | South Africa | Swab | Women | Female | 189 | 41 | 21.69% |
| **Heine, R. P.** | [154] | 1997 | USA | Direct microscopy and Culture methods and PCR | Women | Female | 300 | 61 | 20.3% |
| **Cotch, M. F.** | [71] | 1997 | USA | Culture methods | Women | Female | 13816 | 1741 | 12.6% |
| **Madico, G.** | [236] | 1998 | USA | Swab | Women | Female | 350 | 23 | 6.57% |
| **Passey, M.** | [308] | 1998 | PapuaNew Guinea | PCR | Women | Female | 198 | 92 | 46.46% |
| **Paterson, B. A.** | [309] | 1998 | Australia | PCR | Women | Female | 590 | 93 | 15.76% |
| **Robinson, A. J.** | [338] | 1998 | UK | Swab | Adolescents | Female | 159 | 4 | 2.52% |
| **Bowden, F. J.** | [45] | 1999 | Australia | PCR | Women | Female | 1090 | 273 | 25.05% |
| **Wilkinson, D.** | [412] | 1999 | South Africa | ND | Women | Female | 55974 | 9739 | 17.4% |
| **Beverly, A. L.** | [36] | 1999 | USA | Swab | Women | Female | 260 | 68 | 26.15% |
| **Minkoff, H. L.** | [292] | 1999 | USA | Direct microscopy | HIV Infected and uninfected women | Female | 957 | 221 | 23.09% |
| **Cu-Uvin, S.** | [76] | 1999 | USA | Culture methods | HIV Infected women | Female | 871 | 100 | 11.48% |
| **Divekar, A. A.** | [99] | 2000 | Mumbai | ND | Women | Female | 336 | 20 | 5.9% |
| **Feldblum, P. J.** | [109] | 2000 | Kenya | ELISA | Women | Female | 1922 | 41 | 20.4% |
| **Fonck, K.** | [113] | 2000 | Kenya | PCR | Women | Female | 318 | 41 | 13% |
| **Fonck, K.** | [281] | 2000 | Kenya | PCR and Swab | Women | Female | 621 | 145 | 23.35% |
| **Kamara, P.** | [182] | 2000 | Jamaica | Direct microscopy | Women and Adolescents | Female | 269 | 48 | 17.84% |
| **Rahman, M.** | [327] | 2000 | Bangladesh | Swab | Women | Female | 237 | 108 | 45.57% |
| **Vishwanath, S.** | [393] | 2000 | India | Direct microscopy | Madagascar | Female | 319 | 32 | 10.03% |
| **Watson-Jones, D.** | [406] | 2000 | Tanzanian | Swab | Men | Male | 980 | 109 | 11.12% |
| **Behets, F.** | [34] | 2001 | Madagascar | PCR | Women | Female | 876 | 210 | 24% |
| **Bogaerts, J.** | [39] | 2001 | Bangladesh | Direct microscopy | Women | Female | 2220 | 40 | 1.8% |
| **Claeys, P.** | [69] | 2001 | Azerbaijan | Direct microscopy | Men and Women | Female | 323 | 23 | 7.12% |
|  |  |  |  |  |  | Male | 182 | 8 | 4.4% |
| **Garland, S. M.** | [126] | 2001 | Mongolia | PCR | Women | Female | 198 | 9 | 4.5% |
| **Di Bartolomeo, S.** | [96] | 2001 | Argentina | Culture methods and PCR | Women | Female | 110 | 9 | 8.18% |
| **Joesoef, M. R.** | [174] | 2001 | Indonesia | Culture methods | Women | Female | 116 | 83 | 71.55% |
| **Ortayli, N.** | [303] | 2001 | Turkey | Swab | Women | Female | 569 | 15 | 2.6% |
| **Mahdi, N. K.** | [238] | 2001 | Iran | Direct microscopy | Women | Female | 352 | 46 | 13.01% |
| **Patten, J. H.** | [310] | 2001 | Indonesia. | Direct microscopy | Women | Female | 312 | 47 | 15.1% |
| **Di Bartolomeo, S.** | [95] | 2002 | Argentina | Culture methods | Adolescents | Female | 84 | 2 | 2.4% |
| **Di Bartolomeo, S.** | [94] | 2002 | Argentina | Culture methods | Adults | Female | 784 | 22 | 2.8% |
| **Garrow, S. C** | [128] | 2002 | Australia | Swab | Women | Female | 303 | 49 | 16.1% |
| **Mgone, C. S.** | [257] | 2002 | Papua New Guinea | PCR | Women | Female | 318 | 78 | 24.6% |
| **Knox, J.** | [203] | 2002 | Australia | PCR | Women | Female | 407 | 117 | 28.75% |
| **Aboyeji, A. P.** | [5] | 2003 | Nigeria | Swab | Pregnant women | Female | 230 | 11 | 4.7% |
| **Alary, M.** | [13] | 2003 | Benin | PCR | Men | Male | 404 | 11 | 2.7% |
| **Dan, M.** | [81] | 2003 | Israel | Culture methods and Direct microscopy | Women | Female | 308 | 21 | 6.8% |
| **Davies, S. C.** | [87] | 2003 | Indonesia | Direct microscopy | Sex workers | Female | 288 | 14 | 4.86% |
| **Desai, V. K.** | [92] | 2003 | India | Direct microscopy | Women | Female | 118 | 16 | 13.56% |
| **Lafort, Y.** | [211] | 2003 | Coˆ te D’Ivoire | Swab | Women | Female | 368 | 26 | 7.1% |
| **Laurent, C.** | [213] | 2003 | Senegal | Direct microscopy | Sex workers | Female | 390 | 87 | 22.4% |
| **Lobo, T. T.** | [225] | 2003 | Brazil | Swab | Women | Female | 1008 | 60 | 6% |
| **Soares, V. D.** | [362] | 2003 | Brazil | Direct microscopy | Women | Female | 341 | 35 | 10.26% |
| **Srugo, I.** | [366] | 2003 | Israel | Culture methods (on saline mount) | NGU patients | Male | 68 | 1 | 1.47% |
| **Sullivan, E. A.** | [369] | 2003 | Vanuatu | PCR | Pregnant women | Female | 547 | 150 | 27.5% |
| **Vuylsteke, B. L.** | [398] | 2003 | Coˆ te D’Ivoire | Gold standard diagnostics | Sex workers | Female | 842 | 141 | 16.7% |
| **Anorlu,R.** | [22] | 2004 | Nigeria | Swab | Women | Female | 140 | 6 | 4.3% |
| **Garcia,A.** | [123] | 2004 | Portugal | Swab | Women | Female | 202 | 63 | 31.2% |
| **Kaul,P.** | [187] | 2004 | India | PCR | Women | Female | 1000 | 38 | 3.8% |
| **Nessa,K.** | [279] | 2004 | Bangladesh | Direct microscopy | Sex Workers | Female | 228 | 10 | 4.3% |
| **Miller, W. C.** | [326] | 2004 | Malawi | ND | ND | ND | 431 | 54 | 12.5% |
| **Otuonye,N.M.** | [304] | 2004 | Nigeria | Direct microscopy | Patients | Female | 241 | 5 | 2.1% |
| **Sullivan,E.A.** | [370] | 2004 | Samoa | PCR | Pregnant women | Female | 427 | 89 | 20.8% |
| **Amindavaa, O.** | [19] | 2005 | Mongolia | PCR | Pregnant women | Female | 2000 | 133 | 6.7% |
| **Bradshaw, C. S.** | [47] | 2005 | Australia | PCR | Drug users | ND | 195 | 3 | 2% |
| **Ononge, S.** | [301] | 2005 | Uganda | ND | Sexually  assaulted females | Female | 58 | 1 | 1.7% |
| **Dunkle, K. L.** | [104] | 2005 | South Africa | Direct microscopy | Sex Workers | Female | 285 | 48 | 16.8% |
| **Gare, J.** | [125] | 2005 | Papua New Guinea | Swab | Sex Workers | Female | 211 | 108 | 51% |
| **Huppert, J. S.** | [163] | 2005 | USA | OSOM and Swab | Women | Female | 439 | 89 | 20.3% |
| **Kissinger, P. J.** | [200] | 2005 | USA | Swab | HIV Infected women | Female | 216 | 29 | 13.3% |
| **Goto, A.** | [141] | 2005 | Vietnam | Direct microscopy | Pregnant women | Female | 505 | 7 | 1.39% |
| **Kim, A. A.** | [194] | 2005 | Cambodia | Trich In Pouch | Sex Workers | Female | 90 | 11 | 12.2% |
| **Leutscher, P.** | [222] | 2005 | Madagascar | Swab | Men and Women | Female | 333 | 78 | 23.4% |
|  |  |  |  |  |  | Male | 310 | 23 | 7.4% |
| **Leutscher, P. D. C** | [221] | 2005 | Madagascar | Swab | Men | Male | 240 | 15 | 6% |
| **Nessa, K** | [278] | 2005 | Bangladesh | Swab | Sex Workers | Female | 439 | 33 | 7.5% |
| **Pepin, J.** | [315] | 2005 | Ghana Benin | PCR | Sex Workers | Female | 826 | 191 | 23.12% |
| **Smith, K. S.** | [360] | 2005 | Australia | PCR | Women | Female | 205 | 50 | 24% |
| **Huppert, J. S.** | [163] | 2005 | USA | OSOM and Swab | Women | Female | 439 | 89 | 20.3% |
| **Kissinger, P. J.** | [200] | 2005 | USA | Swab | HIV Infected women | Female | 216 | 29 | 13.3% |
| **Miller, W. C.** | [260] | 2005 | USA | PCR | People | Female | 6533 | 183 | 2.8% |
|  |  |  |  |  |  | Male | 5916 | 101 | 1.7% |
| **Plitt, S. S.** | [324] | 2005 | USA | PCR | Drug users | Female | 115 | 10 | 8.6% |
|  |  |  |  |  |  | Male | 211 | 4 | 1.9% |
| **Watts, D. H** | [407] | 2005 | USA | Swab | HIV Infected women | Female | 1617 | 97 | 6% |
| **Boyer, C. B.** | [46] | 2006 | USA | Swab | Women | Female | 1550 | 26 | 1.7% |
| **Hardick, A** | [150] | 2006 | USA | PCR | ND | Female | 321 | 57 | 17.8% |
|  |  |  |  |  |  | Male | 290 | 13 | 4.5% |
| **Sutton, M. Y.** | [371] | 2006 | USA | PCR | Women | Female | 1999 | 62 | 3.1% |
| **Takei, H.** | [373] | 2006 | USA | Direct microscopy | Women | Female | 904 | 100 | 11.1% |
| **Watts, D. H.** | [408] | 2006 | USA | Direct microscopy | HIV Infected women | Female | 254 | 17 | 6.1% |
| **Chen, X. S.** | [62] | 2006 | China | Swab | Pregnant women | Female | 504 | 16 | 3.2% |
| **Zhao, F. H.** | [422] | 2006 | China | ND | Women | Female | 8798 | 1845 | 21% |
| **Kabakchieva, E.** | [177] | 2006 | Bulgaria | Direct microscopy | Men | Male | 286 | 25 | 8.7% |
| **Rassjo, E. B.** | [333] | 2006 | Uganda | Swab | Adolescents | Female | 199 | 16 | 8% |
| **Tann, C. J.** | [374] | 2006 | Uganda | Swab | Pregnant women | Female | 250 | 43 | 17.3% |
| **Uma, S.** | [383] | 2006 | India | Swab | Women | Female | 487 | 26 | 5.3% |
| **Hitti, J.** | [158] | 2007 | USA | ND | Pregnant women | Female | 11910 | 1439 | 12.08% |
| **Huppert, J. S.** | [164] | 2007 | USA | Swab | Indonesia | Female | 296 | 53 | 18% |
| **Adeoye, G. O.** | [6] | 2007 | Nigeria | Direct microscopy | Patients | Female | 544 | 18 | 3.3% |
| **Chersich, M. F.** | [63] | 2007 | Kenya | Direct microscopy | Sex workers | Female | 680 | 118 | 17.35% |
| **Frohlich, J. A** | [117] | 2007 | South Africa | PCR | Women | Female | 274 | 64 | 23.36% |
| **García, P. J.** | [124] | 2007 | Peru | PCR | Women | Female | 118 | 7 | 5.9% |
| **Hagan, J. E.** | [148] | 2007 | Mongolia | Direct microscopy | Women | Female | 132 | 37 | 28% |
| **Hoke, T. H.** | [160] | 2007 | Madagascar | Direct microscopy | Sex workers | Female | 901 | 658 | 74.2% |
| **Landes, M.** | [212] | 2007 | Ukraine | Culture methods | HIV Infected women | Female | 1050 | 127 | 12.1% |
| **Msuya, S. E.** | [266] | 2007 | Tanzania | Direct microscopy | Women | Female | 2654 | 127 | 5% |
| **Nagot, N.** | [273] | 2007 | Burkina Faso | Direct microscopy | Sex workers | Female | 273 | 9 | 3.3% |
| **Nelson, A.** | [277] | 2007 | Peru | PCR | Couples | Female | 195 | 16 | 8.2% |
| **Pillay, A.** | [320] | 2007 | South Africa | PCR | Patients | Male | 195 | 3 | 1.5% |
|  |  |  |  |  |  | Female | 119 | 73 | 61.3% |
| **Ross, D. A.** | [341] | 2007 | Tanzania | Swab | Adolescents | Female | 1492 | 383 | 25.8% |
| **Black, V.** | [37] | 2008 | South Africa | PCR | Patients | Male | 2024 | 2 | 0.1% |
|  |  |  |  |  |  | Female | 664 | 42 | 6.3% |
| **Fotinatos , N.** | [114] | 2008 | Vanuatu | Direct microscopy | Women | Female | 905 | 229 | 25.3% |
| **Miller, M.** | [259] | 2008 | USA | Swab | Women | Female | 135 | 51 | 38% |
| **Willers, D. M.** | [413] | 2008 | USA | Culture methods | Women | Female | 205 | 45 | 22% |
| **Hewett, P. C.** | [156] | 2008 | Brazil | PCR | Women | Female | 781 | 3 | 0.38% |
| **Kumarasamy, N.** | [208] | 2008 | India | Culture methods | People | Female | 244 | 15 | 6% |
| **Madhivanan, P.** | [233] | 2008 | India | Swab | Women | Female | 863 | 71 | 8.2% |
| **Mahto, M.** | [240] | 2008 | UK | ND | Women | Female | 474 | 39 | 8.2% |
| **Nguyen, T. V.** | [284] | 2008 | Vietnam | Direct microscopy | Sex workers | Female | 406 | 35 | 8.9% |
| **Rahman, S.** | [328] | 2008 | Bangladesh | Direct microscopy | Patients | Female | 399 | 12 | 8% |
| **Reza-Paul, S.** | [336] | 2008 | India | PCR | Sex workers | Female | 429 | 141 | 32.9% |
| **Schnatz, P. F.** | [347] | 2008 | Nigeria | Direct microscopy | Patients | Female | 199 | 18 | 9% |
| **Zribi, M.** | [425] | 2008 | Tunisia | Culture methods | Women | Female | 116 | 6 | 5% |
| **Wang, H. B.** | [402] | 2008 | China | Swab | Sex workers | Female | 737 | 78 | 10.6% |
| **Xu, J. J.** | [415] | 2008 | China | ND | Sex workers | Female | 96 | 21 | 22.1% |
| **Wang, H.** | [400] | 2009 | China | Culture methods | Sex workers | Female | 270 | 29 | 10.7% |
| **Zhang, X. J.** | [420] | 2009 | China | Direct microscopy | Women | Female | 2423 | 109 | 4.5% |
| **Bahram, A.** | [29] | 2009 | Iran | Direct microscopy | Women | Female | 500 | 33 | 6.6% |
| **Bruins, M. J.** | [51] | 2009 | Tanzania | Culture methods | HSV-2 positive women | Female | 1305 | 395 | 30.3% |
| **Gander, S.** | [122] | 2009 | Canada | Direct microscopy | Adolescents | Female | 119 | 6 | 5% |
| **Ghebremichael,** | [133] | 2009 | Tanzania | PCR | Women | Female | 219 | 24 | 10.9% |
| **Pattullo, L.** | [311] | 2009 | USA | ND | Adolescents | Female | 345 | 65 | 18.8% |
| **Di Clemente, R.** | [352] | 2009 | USA | PCR | Adolescents | Female | 715 | 92 | 12.9% |
| **Gallion, H. R.** | [121] | 2009 | USA | Culture methods | Adolescents | Female | 274 | 12 | 4% |
| **Gaydos, C. A.** | [130] | 2009 | USA | NAAT | Patients | Male | 290 | 10 | 3.4% |
| **Gray, R. H.c** | [146] | 2009 | Uganda | Swab | Women | Female | 402 | 45 | 11.2% |
| **Harijaona, V.** | [151] | 2009 | Madagascar | Direct microscopy | Sex workers | Female | 100 | 32 | 32% |
| **Hawkes, S.** | [153] | 2009 | Pakistan | Swab | Sex workers | Female | 426 | 18 | 4.3% |
| **Kapina, M.** | [183] | 2009 | Zambia | ND | Women | Female | 244 | 9 | 3.7% |
| **Kosambiya, J. K.** | [206] | 2009 | India | Culture methods | Women | Female | 102 | 42 | 41% |
| **Leon, S. R.** | [220] | 2009 | Peru | Culture methods | Women | Female | 319 | 29 | 9.1% |
| **Madhivanan, P.** | [234] | 2009 | India | Direct microscopy | women | Female | 898 | 76 | 8.5% |
| **McIver, C. J.** | [249] | 2009 | Australia | Swab | Patients | Female | 175 | 7 | 4% |
| **Mehta, S. D.** | [254] | 2009 | Kenya | Culture methods | Patients | Male | 2655 | 57 | 2.14% |
| **Msuya, S. E.** | [267] | 2009 | Tanzania | Culture methods | Pregnant women | Female | 2654 | 133 | 5% |
| **Nguyen, T. V.c** | [285] | 2009 | Vietnam | Direct microscopy | Sex workers | Female | 395 | 35 | 8.9% |
| **Shahmanesh, M.** | [353] | 2009 | India | ND | Sex workers | Female | 326 | 31 | 9.4% |
| **Paz-Bailey, G.** | [313] | 2009 | Honduras | ND | People | Female | 382 | 61 | 16.4% |
| **Rao, V. G** | [332] | 2009 | India | Swab | People | Male | 409 | 17 | 4.1% |
|  |  |  |  |  |  | Female | 167 | 72 | 43.1% |
| **Saleh-Onoya, D.** | [344] | 2009 | South Africa | Swab | HIV Infected women | Female | 49 | 11 | 22% |
| **Tibaldi, C.** | [376] | 2009 | Italy | Direct microscopy | Women | Female | 24300 | 382 | 1.6% |
| **Sobngwi-Tambekou, J.** | [363] | 2009 | South Africa | PCR | Men | Male | 878 | 28 | 3.2% |
| **Upcroft, J. A.** | [384] | 2009 | Papua New Guinea | PCR | Women | Female | 82 | 27 | 32.9% |
| **Verteramo, R.** | [391] | 2009 | Rome | Direct microscopy | HPV-positive | Female | 266 | 3 | 1.1% |
| **Al-Awadhi, R** | [11] | 2010 | Kuwait | Swab | Women | Female | 2679 | 319 | 11.9% |
| **Almeida, M. S.** | [15] | 2010 | Brazil | PCR | Women | Female | 206034 | 7349 | 3.6% |
| **Dai, Q.** | [79] | 2010 | China | Direct microscopy | Women | Female | 397 | 10 | 2.5% |
| **Becker, M.** | [33] | 2010 | India | PCR | People | Female | 73 | 12 | 16.4% |
| **Bruce, E.** | [50] | 2010 | Papua New Guinea | PCR | Sex workers | Male | 117 | 10 | 8.5% |
|  |  |  |  |  |  | Female | 129 | 53 | 41% |
| **Canchihuaman, F. A.** | [56] | 2010 | Peru | Swab | Couples | Female | 2078 | 87 | 4.18% |
| **Chai, S. J.** | [58] | 2010 | USA | PCR | Men | Male | 2052 | 26 | 1.26% |
| **Chalechale, A.** | [59] | 2010 | Iran | ND | Women | Female | 33690 | 300 | 0.9% |
| **Choudhry, S.** | [68] | 2010 | India | Direct microscopy | Patients | Female | 108 | 14 | 13% |
| **Crucitti,T.** | [74] | 2010 | Zambia | PCR | Adolescents | Female | 460 | 113 | 24.6% |
| **Gondo, Dcaf** | [140] | 2010 | Brazil | Culture methods | Pregnant women | Female | 307 | 99 | 32.2% |
|  |  |  |  |  | Sex workers | Female | 197 | 65 | 33.2% |
|  |  |  |  |  | Pregnant women | Female | 289 | 1 | 0.04% |
| **Dahab, M.** | [78] | 2010 | Sudan | Questionnaire | Women | Female | 2473 | 297 | 12% |
| **Huq, M.** | [165] | 2010 | Bangladesh | PCR | Sex workers | Female | 156 | 11 | 7% |
| **Karabulut, A.** | [184] | 2010 | Turkey | Questionnaire | Women | Female | 19639 | 143 | 0.7% |
| **Kurewa, N. E.** | [209] | 2010 | Zimbabwe | Direct microscopy | Pregnant women | Female | 691 | 82 | 11.8% |
| **Le Roux, M. C.** | [216] | 2010 | South Africa | Direct microscopy | Men | Male | 300 | 24 | 8% |
| **De Jongh, M.** | [89] | 2010 | South Africa | Direct microscopy and Culture methods | Women | Female | 54 | 6 | 11.1% |
| **Lusk, M. J.** | [231] | 2010 | Australia | PCR | Women | Female | 356 | 17 | 4.8% |
| **Månsson, F.** | [242] | 2010 | Guinea-Bissau | Direct microscopy | Women | Female | 671 | 137 | 20.4% |
| **Menéndez, C.** | [256] | 2010 | Mozambique | Direct microscopy | Women | Female | 254 | 78 | 31% |
| **Mhlongo,S.** | [258] | 2010 | South Africa | PCR | People | Female | 300 | 88 | 29.3% |
| **Nomelini,R.S.** | [290] | 2010 | Brazil | ND | Women | Male | 507 | 38 | 7.5% |
|  |  |  |  |  |  | Female | 13881 | 440 | 3.17% |
| **Nwadioha, S.** | [293] | 2010 | Nigeria | Direct microscopy | Women | Female | 600 | 30 | 5% |
| **Ong,V.A.** | [300] | 2010 | Philippines | PCR | Sex workers | Female | 377 | 36 | 9.55% |
| **Perazzi,B.E.** | [316] | 2010 | Argentina | Culture methods | Pregnant women | Female | 597 | 24 | 4.0% |
| **Piperaki, E. T.** | [322] | 2010 | Greece | ND | Women | Female | 502 | 23 | 4.6% |
| **Turner, A. N.** | [382] | 2010 | Madagascar | Culture methods | Sex workers | Female | 1000 | 356 | 35.6% |
| **Vaca, M.** | [387] | 2010 | Ecuador | Direct microscopy | Adolescents | Female | 214 | 1 | 0.5% |
| **Zaki, M. E.** | [419] | 2010 | Egypt | Culture methods | Patients | Female | 110 | 33 | 30% |
| **Anisimova, N.** | [21] | 2011 | Russia | Direct microscopy | Women | Female | 1125 | 13 | 1.16% |
| **Crucitti, T.** | [75] | 2011 | Zambia | PCR | Adolescents | Female | 439 | 119 | 27.1% |
| **Wang, H. B.** | [401] | 2011 | China | Direct microscopy | Sex workers | Female | 737 | 78 | 10.6% |
| **Domeika, M.** | [101] | 2011 | Russia | Real-Time PCR | Women | Female | 319 | 4 | 1.2% |
| **Djigma, F.** | [100] | 2011 | Burkina Faso | Direct microscopy | HIV Infected women | Female | 251 | 2 | 0.8% |
| **Fernando, S. D.** | [111] | 2011 | Sri Lanka | Swab | Patients | Female | 346 | 24 | 6.9% |
| **Ghebremichael, M.** | [132] | 2011 | Tanzania | PCR | Men | Male | 588 | 38 | 6.4% |
| **Goins, J** | [138] | 2011 | Nicaragua | PCR | Women | Female | 91 | 12 | 13.19% |
| **Houso, Y.** | [162] | 2011 | Palestine | Culture methods | Women | Female | 1207 | 164 | 13.6% |
| **Khan, M. S.** | [191] | 2011 | Pakistan | Culture methods | Sex workers | Female | 730 | 37 | 5% |
| **Kim, S. J.** | [196] | 2011 | Korea | PCR | People | Female | 279 | 7 | 2.5% |
| **Luppi, C. G.** | [230] | 2011 | Brazil | PCR | Women | Male | 430 | 1 | 0.23% |
|  |  |  |  |  |  | Female | 818 | 26 | 3.2% |
| **Mawu,F.O.** | [246] | 2011 | Indonesia | Swab | Sex workers | Female | 217 | 49 | 22.6% |
| **Mehta, S.** | [252] | 2011 | Kenya | PCR | Patients | Male | 526 | 14 | 2.7% |
| **Miranda, A.** | [261] | 2011 | Brazil | ND | Women | Female | 33 | 84 | 1.2% |
| **Oakeshott, P.** | [296] | 2011 | UK | ND | Adolescents | Female | 183 | 2 | 1.1% |
| **Özdemir, E.** | [306] | 2011 | Turkey | PCR | Men | Male | 80 | 2 | 2.5% |
| **Das, A.** | [82] | 2011 | India | ND | ND | ND | 399 | 124 | 31.1% |
| **Rao,K.** | [331] | 2011 | India | OSOM | Women | Female | 417 | 68 | 16.3% |
| **Rathod, S. D.** | [335] | 2011 | India | Direct microscopy | Women | Female | 775 | 23 | 3.0% |
| **Silitonga,N.** | [357] | 2011 | Indonesia | ND | Sex workers | Female | 330 | 45 | 13.6% |
| **Muzny, C. A.** | [271] | 2011 | USA | Culture methods | Women | Female | 196 | 36 | 18.3% |
| **Strathdee, S. A.** | [367] | 2011 | Mexico | ND | Sex workers | Female | 620 | 217 | 35% |
| **Rogers, S.** | [340] | 2011 | USA | ND | ND | ND | 2120 | 179 | 8.4% |
| **Sieck, C. J.** | [356] | 2011 | USA | Swab | ND | Female | 1242 | 192 | 15.4% |
| **Afeke, I.** | [7] | 2012 | Ghana | ND | Women | Female | 788 | 18 | 2.3% |
| **Bankar, S. M.** | [31] | 2012 | India | ND | Patients | Female | 200 | 14 | 7% |
| **Timm, N.** | [377] | 2011 | USA | PCR | Adolescents | Male | 270 | 14 | 5% |
| **Boon, M. E.** | [43] | 2012 | Netherlands | ND | ND | ND | 964 | 25 | 2.6% |
| **Bonney** | [42] | 2012 | USA | PCR | ND | ND | 108 | 29 | 26.8% |
| **Boon, M. E.** | [44] | 2012 | Netherlands | ND | Women | Female | 498405 | 949 | 0.19% |
| **Chiduo, M.** | [65] | 2012 | Tanzania | Direct microscopy | HIV Infected women | Female | 85 | 16 | 18.8% |
| **Vuylsteke, B.** | [395] | 2012 | Côte d'Ivoire | ND | ND | ND | 1204 | 141 | 11.7% |
| **Vuylsteke, B.** | [397] | 2012 | Côte d'Ivoire | PCR | Sex workers | Male | 96 | 2 | 2.1% |
| **Creswell, J.** | [73] | 2012 | El Salvador | PCR | Sex workers | Male | 648 | 7 | 1.08% |
| **Brown, J. L.** | [49] | 2012 | USA | Real-Time PCR | Adolescents | Female | 964 | 25 | 2.6% |
| **Cosentino, L. A.** | [20] | 2012 | USA | ND | People | Female | 272 | 24 | 8.8% |
|  |  |  |  |  |  | Male | 225 | 2 | 0.9% |
| **Ginocchio, C. C.** | [137] | 2012 | USA | ATV | Women | Female | 7593 | 663 | 8.7% |
| **Mayer, K. H.** | [247] | 2012 | USA | PCR | Women | Female | 119 | 17 | 14% |
| **Nijhawan, A. E.** | [286] | 2012 | USA | TMA | Women | Female | 378 | 53 | 14% |
| **Fernando, S. D.** | [110] | 2012 | Sri Lanka | Culture methods | Patients | Female | 346 | 25 | 7.2% |
| **Bonney, L. E.** | [42] | 2012 | India | ND | ND | ND | 108 | 29 | 26.9% |
| **Caiyan, X.** | [54] | 2012 | China | Direct microscopy | Women | Women | 6339 | 58 | 15% |
| **Fule, S. R.** | [119] | 2012 | India | Direct microscopy | Women | Female | 156 | 19 | 12% |
| **Ghosh, I.** | [134] | 2012 | India | Direct microscopy | HIV Infected women | Female | 35 | 8 | 22.9% |
| **Kalantari, N.** | [180] | 2012 | Iran | Direct microscopy | Non-pregnant women | Female | 176 | 2 | 1.3% |
| **Karou,S.D.** | [186] | 2012 | Burkina Faso | Swab | Women | Female | 1536 | 16 | 1.04% |
| **Lewis, D. A.** | [223] | 2012 | South Africa | Real-Time PCR | Patients | Female | 558 | 57 | 10.2% |
| **Mascarenhas,R.E.** | [243] | 2012 | Brazil | Papanicolaou’s technique | Adolescents | Male | 550 | 27 | 4.9% |
|  |  |  |  |  |  | Female | 100 | 1 | 1% |
| **Mehta, S. D.** | [253] | 2012 | Kenya | Culture methods | Man | Male | 526 | 14 | 2.67% |
| **Ng, A.** | [280] | 2012 | UK | NAAT | Women | Female | 2020 | 72 | 3.6% |
| **Mitteregger, D.** | [264] | 2012 | Austria | PCR | Patients | Male | 86 | 29 | 34% |
| **Ramia, S.** | [330] | 2012 | Lebanon | PCR | Women | Female | 441 | 101 | 22.9% |
| **Mlisana, K.** | [265] | 2012 | South Africa | PCR | Women | Female | 241 | 49 | 20.3% |
| **Ovalle, A.** | [305] | 2012 | Chile | ND | Women | Female | 255 | 6 | 2.4% |
| **Paul, H.** | [312] | 2012 | India | Direct microscopy | Women | Female | 198 | 6 | 3% |
| **Paz-Bailey, G.** | [314] | 2012 | El Salvador | PCR | People | Female | 383 | 36 | 9.4% |
| **Perla, M. E.** | [318] | 2012 | Peru | Direct microscopy | Sex workers | Male | 361 | 1 | 0.3% |
|  |  |  |  |  |  | Female | 212 | 5 | 2.4% |
| **Rasti,S.** | [334] | 2012 | Iran | Direct microscopy | Pregnant women | Female | 450 | 2 | 0.5% |
| **Ryder,N.** | [343] | 2012 | Australia | Real-Time PCR | Women | Female | 356 | 30 | 8.4% |
| **Vandepitte, J.** | [390] | 2012 | Uganda | Real-Time PCR | Sex workers | Female | 1025 | 176 | 17.1% |
| **Vuylsteke, B.** | [396] | 2012 | Coˆte d’Ivoire | ND | Sex workers | Female | 1110 | 139 | 12.5% |
| **Vuylsteke, B.** | [396] | 2012 | Coˆte d’Ivoire | PCR | Sex workers | Male | 94 | 2 | 2.1% |
| **Abbai, N. S.** | [1] | 2013 | South Arica | ND | Women | Female | 2236 | 200 | 10% |
| **Alvarez Rodríguez, B.** | [17] | 2013 | Brazil | ND | Sex workers | Female | 220 | 40 | 18.18% |
| **Brooks-Smith-Lowe, K.** | [48] | 2013 | Grenada | ND | Women | Female | 2677 | 23 | 0.85% |
| **Datcu, R.** | [84] | 2013 | Denmark | PCR | Women | Female | 196 | 1 | 0.5% |
| **Wang, H.** | [399] | 2013 | China | PCR | Sex workers | Female | 334 | 22 | 6.6% |
| **de Lima, M. C. L.** | [90] | 2013 | Brazil | Swab | Women | Female | 314 | 33 | 10.5% |
| **Dharma Vijaya, M. N.** | [93] | 2013 | India | Culture methods | Women | Female | 750 | 16 | 2.1% |
| **Drake, A. L.** | [103] | 2013 | Kenya | Direct microscopy | Pregnant women | Female | 1156 | 23 | 2% |
| **Grama, D. F.** | [142] | 2013 | Brazil | Direct microscopy and Culture methods | Women | Female | 724 | 19 | 2.6% |
| **Jansen, K.** | [171] | 2013 | Germany | APTIMA | Sex workers | Female | 227 | 37 | 16.2% |
| **Kim, T. H.** | [197] | 2013 | Korea | PCR | Women | Female | 532 | 3 | 0.6% |
| **Kwon, I.** | [210] | 2013 | Australia | PCR | Women | Female | 781 | 3 | 0.38% |
| **Kim, j. k.** | [198] | 2013 | South Korea | ND | ND | ND | 1618 | 42 | 2.6% |
| **Lewis, d. a.** | [263] | 2013 | South Africa | ND | Patients | Male | 822 | 51 | 6.2% |
| **Madhivanan, P.** | [235] | 2013 | India | OSOM | Women | Female | 418 | 62 | 14.8% |
| **Mendoza, L.** | [255] | 2013 | Paraguay | Culture methods | Women | Female | 181 | 19 | 10.5% |
| **Naidoo, K.** | [274] | 2013 | South Africa | ND | HIV Infected women | Female | 750 | 41 | 5.5% |
| **Nourian, A.** | [291] | 2013 | Iran | Direct microscopy | Pregnant women | Female | 1000 | 33 | 3.3% |
| **Oliphant, J.** | [299] | 2013 | New Zealand | Culture methods | Women | Female | 261 | 5 | 1.9% |
| **Singh, R. H.** | [359] | 2013 | USA | Swab | Patients | Female | 200 | 38 | 19% |
| **Javanbakht , Mc** | [173] | 2013 | USA | NAAT | Women | Female | 358 | 78 | 21.8% |
| **Munson, K. L.** | [269] | 2013 | USA | NAAT | Men | Male | 622 | 42 | 6.6% |
| **Muzny, C. A.** | [270] | 2013 | USA | ND | Patients | Female | 478 | 83 | 17.4% |
| **Pollett, S** | [325] | 2013 | Peru | Direct microscopy | Sex workers | Female | 99 | 4 | 4.0% |
| **Rukasha, I.** | [342] | 2013 | South Africa | Direct microscopy | Women | Female | 380 | 30 | 8% |
| **Silva, L. C. F.** | [358] | 2013 | Brazil | ND | HIV Infected women | Female | 341 | 14 | 4.1% |
| **Tolosa, J.** | [381] | 2013 | Colombia | PCR | Patients | Female | 1372 | 11 | 0.8% |
| **Vijaya Mn, D.** | [392] | 2013 | India | Culture methods | Women | Female | 750 | 16 | 2.1% |
| **McCormick, D. F.** | [248] | 2013 | Bangla | Direct microscopy | Sex workers | Female | 549 | 16 | 2.9% |
| **Arora, B. B.** | [24] | 2014 | India | ND | ND | ND | 12622 | 2379 | 18.8% |
| **Abdelaziz, Z. A.** | [2] | 2014 | Sudan | Direct microscopy (Gram smear) and Culture methods | Pregnant women | Female | 200 | 1 | 0.5% |
| **Arbabi, M.** | [23] | 2014 | Iran | Culture methods and direct microscopy | People | Female | 1205 | 24 | 2% |
| **Francis, S. C.** | [115] | 2014 | Tanzania. | Swab | Women | Female | 945 | 184 | 19% |
| **Kalantari, N.** | [179] | 2014 | Iran | Papanicolaou test | Women | Female | 33600 | 71 | 0.2% |
| **Zhou,H.** | [423] | 2014 | China | TCT | Women | Female | 154 | 24 | 15.6% |
| **Lazenby, G. B.** | [214] | 2014 | Tanzania | NAAT | Women | Female | 799 | 1 | 0.1% |
| **Kim, Y.** | [199] | 2014 | Korea | PCR | Women | Female | 324 | 34 | 10.4% |
| **Maghsoudi, R.** | [237] | 2014 | Iran | Disk Diffusion Method | Patients | Female | 66 | 4 | 6% |
| **Rocha, D. A. P.** | [339] | 2014 | Brazil | PCR | Women | Female | 361 | 46 | 12.7% |
| **Seo, J. H.** | [351] | 2014 | Korea | PCR | Patients | Male | 201 | 8 | 4.0% |
| **Vallely,A.** | [388] | 2014 | Papua New Guinea | Swab | Women | Female | 46866 | 273 | 0.1% |
| **Miranda,A.E.** | [262] | 2014 | Brazil | Swab | Pregnant women | Female | 299 | 23 | 7.7% |
| **Aloui, Dorsaf** | [16] | 2015 | Tunisian | Swab | People | Female | 924 | 32 | 3.5% |
| **Bakar, M. S. A.** | [30] | 2015 | UK | NAAT | Women and men | Female | 372 | 10 | 2.7% |
| **da Luz Becker, D.** | [77] | 2015 | Brazil | PCR | People | Female | 530 | 24 | 4.5% |
| **Emma Hathorn,** | [152] | 2015 | UK | TMA | Asymptomatic | Male | 530 | 6 | 1.1% |
|  |  |  |  |  |  | Female | 2020 | 72 | 3.6% |
| **Etuketu Maureen** | [106] | 2015 | Nigeria | HVS | Women | Male | 1483 | 21 | 1.4% |
|  |  |  |  |  |  | Female | 300 | 31 | 10.3% |
| **Jennifer Gratrix** | [143] | 2015 | Canada | Gen-Probe Apti-  ma TV test | People | Female | 1055 | 29 | 2.8% |
| **Khatib, N.** | [193] | 2015 | UK | PCR and Gen-Probe | Men | Male | 1218 | 3 | 0.2% |
|  |  |  |  |  |  | Male | 205 | 28 | 13.7% |
| **Mahmoud, A.** | [239] | 2015 | Egypt | Direct microscopy | Women | Female | 1000 | 50 | 5% |
| **Mario Sviben** | [372] | 2015 | Croatia | PCR | People | Male | 700 | 57 | 8.2% |
| **Ok Atılgan, A.** | [298] | 2015 | Turkey | PCR | Patients | Female | 111 | 2 | 1.8% |
| **Plaas, K** | [323] | 2015 | Estonian | PCR | Patients | Male | 233 | 24 | 10.3% |
| **Nyein, S.** | [294] | 2015 | UK | OSOM | Women | Female | 58 | 3 | 5.2% |
| **R A Wangnapi** | [404] | 2015 | Papua New Guinea | PCR | Pregnant women | Female | 4002 | 73 | 1.3% |
| **Samarawickrema, N. A.** | [345] | 2015 | Sri Lanka | PCR | Women | Female | 483 | 11 | 2.3% |
| **Jonathan Shaw** | [354] | 2015 | UK | NAAT | People | ND | 961 | 28 | 2.9% |
| **Ahmadnia, E.** | [9] | 2016 | Iran | PCR | Women | Female | 4274 | 60 | 1.4% |
| **Luo, L.** | [229] | 2016 | China | ND | Sex Workers | Female | 734 | 66 | 9% |
| **Badman, S. G.** | [27] | 2016 | Papua New Guinea. | ND | Women | Female | 125 | 47 | 37.6% |
| **Camporiondo, M. P.** | [55] | 2016 | Rome | PCR | Women | Female | 309 | 4 | 1.3% |
| **Cíntia Lima AMBROZIO** | [18] | 2016 | Brazil | PCR | Women | Female | 300 | 27 | 9% |
| **Kerubo, E.** | [190] | 2016 | Kenya | NAAT | Adolescents | Female | 510 | 15 | 3% |
| **Lee, A.** | [218] | 2016 | Korea | PCR | Women | Female | 3460 | 25 | 3% |
| **Lee, J.** | [219] | 2016 | Korea | PCR | Adolescents | Male | 237 | 2 | 0.8% |
| **Wynn, A.** | [414] | 2016 | South Africa | ND | ND | ND | 300 | 18 | 6% |
| **Lisa M. Vallely** | [28] | 2016 | Papua New Guinea | PCR | Pregnant women | Female | 7652 | 171 | 2.9% |
| **Mabonga, E.** | [232] | 2016 | Uganda | NAAT | HIV Infected women | Female | 110 | 2 | 1.8% |
| **Maina, A. N.** | [241] | 2016 | Kenya | Direct microscopy | People | Female | 249 | 1 | 0.4% |
| **Snead, M.** | [361] | 2016 | Jamaica | STIs | Women | Female | 335 | 77 | 23% |
| **van der Veer, C.** | [389] | 2016 | Netherlands. | Molecular tests. | Man | Male | 1204 | 6 | 0.5% |
| **Elise D Riley** | [337] | 2016 | USA | ND | Women | Female | 245 | 29 | 12% |
| **Seay, J.** | [349] | 2017 | USA | PCR | Women | Female | 246 | 23 | 9.3% |
| **Agyarko-Poku, T.** | [8] | 2017 | Ghana | PCR | Women | Female | 500 | 22 | 4.5% |
| **Akinbo, F. O.** | [10] | 2017 | Nigeria | Wet mount examination and Culture methods | Adolescents | Female | 272 | 25 | 9.2% |
| **Carrillo-Ávila, J. A.** | [57] | 2017 | Spain | Nucleic acid hybridization system | Non-pregnant women | Female | 5203 | 127 | 2.4% |
| **Chris R. Kenyon** | [189] | 2017 | UK | ND | Women | Female | 9053 | 428 | 4.7% |
| **Costa-Lira, E.** | [70] | 2017 | Brazil | PCR | Women | Female | 180 | 24 | 18.04% |
| **Frati, E. R.** | [116] | 2017 | Italy | Direct microscopy | Women | Female | 537 | 22 | 4.08% |
| **Gatti, F. A.** | [129] | 2017 | Brazil | PCR | Women | Female | 345 | 14 | 4.1% |
| **Glehn, M. P.** | [394] | 2017 | Brazil | ND | Women | Female | 193 | 30 | 16% |
| **Ginindza, T. G.** | [136] | 2017 | Swaziland | Gen-Probe Apti-  ma TV test | Women | Female | 655 | 55 | 8.4% |
| **Gratrix, J.** | [144] | 2017 | Canada | Direct microscopy | STI clinic attendees | Female | 1042 | 29 | 2.8% |
| **Ignacio, M. A. O.** | [166] | 2017 | Brazil | PCR | Lesbians | Male | 1212 | 3 | 0.2% |
|  |  |  |  |  |  | Female | 100 | 1 | 1.1% |
| **Jillian Pintye** | [321] | 2017 | Kenya | Wet mount examination and Direct microscopy | Pregnant women | Female | 1271 | 81 | 6.0% |
| **Kim, H. J.** | [195] | 2017 | Korea | PCR | Patients | Male | 436 | 1 | 0.2% |
| **Korycińska, J.** | [205] | 2017 | Poland | OSOM | Women | Female | 300 | 2 | 0.7% |
| **Le, P. T.** | [215] | 2017 | Japan | PCR | Man | Male | 109 | 3 | 3% |
| **Masha, S. C.** | [244] | 2017 | Kenya | PCR | Women | Female | 42 | 3 | 7.4% |
| **Matini, M.** | [245] | 2017 | Iran | Direct microscopy and Culture methods | Patients | Female | 862 | 16 | 1.9% |
| **Nateghi Rostami, M.** | [275] | 2017 | Iran | PCR | Women | Female | 420 | 81 | 19.3% |
| **Nguyen, M.** | [282] | 2017 | Vietnam | Gen-Probe Apti-  ma TV test | Pregnant women | Female | 387 | 3 | 0.8% |
| **Park, J. J.** | [307] | 2017 | Korea | PCR | Adolescents | Man and women | 237 | 2 | 0.8% |
| **Richard H. Asmah** | [26] | 2017 | Ghana | Swab | Pregnant women | Female | 992 | 20 | 0.2% |
| **Pereyre, S.** | [317] | 2017 | France | ND | ND | ND | 1516 | 29 | 1.9% |
| **Snead, M. C.** | [317] | 2017 | Jamaica | PCR | Women | Female | 254 | 58 | 23% |
| **Yaro, M. B.** | [416] | 2017 | Nigeria | Direct microscopy | Students | Female | 139 | 29 | 20.9% |
| **Das, P.** | [83] | 2018 | India | PCR | Non-pregnant women | Male | 201 | 20 | 10% |
|  |  |  |  |  |  | Female | 1110 | 151 | 13.6% |
| **Garrett, N. J.** | [127] | 2018 | South Africa | POC testing | HIV Infected women | Female | 267 | 8 | 3.0% |
| **Kaida, A.** | [178] | 2018 | South Africa | PCR | Adolescents | Male | 198 | 16 | 8.1% |
| **Lockhart, A.** | [226] | 2018 | Kenya | PCR | Sex workers | Female | 350 | 32 | 9.2% |
| **Munoz-Ramirez, A.** | [268] | 2018 | Mexico | NAAT | Sex workers | Female | 105 | 25 | 23.8% |
| **Occhionero, M.** | [297] | 2018 | Argentina | ND | Patients | Female | 295 | 11 | 3.73% |
| **Tjagur, S.** | [379] | 2018 | Estonian | ND | Man | Male | 825 | 6 | 0.7% |
| **Ijasan, O.** | [167] | 2018 | Nigeria | Wet mount and Giemsa staining technique | People | Female | 320 | 29 | 9.1% |
| **Upton, A.** | [386] | 2018 | New Zealand | Aptima | STI Patients | Female | 2212 | 77 | 3.5% |
| **Richard Harry Asmah** | [25] | 2018 | Ghanaian | PCR | Patients | Male | 431 | 3 | 0.7% |
|  |  |  |  |  |  | Man and women | 150 | 64 | 42.7% |
| **Davis, A.** | [88] | 2018 | USA | ND | Women | Female | 333 | 77 | 23.1% |
| **Sena, A.** | [350] | 2018 | USA | ND | Women | Female | 619 | 71 | 11.48% |
| **Harbertson, J.** | [149] | 2018 | USA | ND | Men | Male | 2553 | 3 | 0.11% |
| **Schwebke, J.** | [348] | 2018 | USA | ND | ND | ND | 77740 | 8784 | 11.3% |
| **J. Dionne-Odom** | [98] | 2019 | USA | PCR | Patients | Female | 320 | 48 | 15% |
| **S. A. Fallon** | [107] | 2019 | USA | Swab | Patients | Female | 1104 | 103 | 9.3% |
| **C. A. Muzny** | [145] | 2019 | USA | PCR | HIV Infected women | Female | 612 | 110 | 18% |
| **J. N. Park** | [168] | 2019 | USA | PCR | Sex workers | Female | 250 | 121 | 48.5% |
| **M. K. Shaw** | [355] | 2019 | USA | PCR | Patients | Female | 199 | 14 | 7% |
| **Abdul-Aziz, M** | [3] | 2019 | Yemen | Swab | Non-pregnant women | Female | 347 | 4 | 1.15% |
| **M. P. Bruni** | [52] | 2019 | Brazil | PCR | Women | Female | 499 | 21 | 4.1% |
| **A. E. Burton** | [53] | 2019 | Australia | Culture methods | Women | Female | 760 | 93 | 12.2% |
| **P. Chitneni,** | [66] | 2019 | Uganda | PCR | HIV-uninfected women | Female | 150 | 12 | 8.0% |
| **D. L. J. Davey** | [85] | 2019 | South Africa | PCR | Pregnant women | Female | 242 | 75 | 31% |
| **IRYNA BOIKO** | [40] | 2019 | Ukraine | ND | ND | ND | 455 | 47 | 10.3% |
| **M. Diadhiou** | [97] | 2019 | Senegal | Swab | Women | Female | 276 | 7 | 2.5% |
| **G. G. G. Donders** | [102] | 2019 | Belgium | PCR | Patients | Female | 718 | 304 | 42.3% |
| **V. M. Ferre** | [112] | 2019 | Togo | Swab | Sex workers | Female | 310 | 13 | 4.2% |
| **K. Gill** | [135] | 2019 | South Africa | Swab | Adolescents | Female | 200 | 12 | 6% |
| **L. D. C. Gómez-Rodríguez** | [139] | 2019 | Colombia | Swab | Pregnant women | Female | 226 | 1 | 0.4% |
| **O. Guralp** | [147] | 2019 | Cyprus | PCR | Women | Female | 273 | 1 | 0.3% |
| **C. M. Hoffman** | [159] | 2019 | South Africa | PCR | Patients | Female | 251 | 81 | 32% |
| **D. L. Joseph Davey** | [86] | 2019 | South Africa | PCR | Women | Female | 242 | 36 | 15% |
| **Z. Khan** | [192] | 2019 | India | Swab | Women | Female | 550 | 3 | 0.5% |
| **F. M. Kiweewa** | [201] | 2019 | Uganda | PCR | Women | Female | 3440 | 213 | 6.2% |
| **I. Klavs, M. Milavec** | [202] | 2019 | Slovenia | PCR | Women | Female | 593 | 1 | 0.2% |
| **D. G. Konadu** | [204] | 2019 | Canada | Swab | Pregnant women | Female | 589 | 8 | 1.4% |
| **A. Lockhart** | [227] | 2019 | Kenya | PCR | FSW | Female | 350 | 32 | 9.2% |
| **S. Lowe** | [228] | 2019 | Zimbabwe | PCR | HIV-infected women | Female | 385 | 31 | 8.1% |
| **G. Mvumbi** | [120] | 2019 | Congo | Swab | Pregnant women | Female | 352 | 51 | 14.6% |
| **R. Mwatelah** | [272] | 2019 | Kenya | PCR | Women | Female | 870 | 28 | 3.2% |
| **M. Nguyen** | [283] | 2019 | Vietnam | PCR | Pregnant women | Female | 800 | 48 | 6.0% |
| **D. S. Squire** | [365] | 2019 | Ghana | PCR | Patients | Female | 492 | 89 | 18.1% |
| **R. C. Tine** | [378] | 2019 | Senegal | PCR | Patients | Female | 1257 | 50 | 4% |
| **P. Upreti** | [385] | 2019 | Nepal | PCR | Women | Female | 2606 | 188 | 7.2% |
| **A. J. Warr** | [405] | 2019 | Kenya | PCR | Pregnant women | Female | 1221 | 73 | 6% |
| **N. K. S. Al-Majidii** | [12] | 2019 | Iraq | PCR | Swab | Female | 226 | 170 | 75.22% |
| **M. D. Barbosa** | [32] | 2020 | Brazil | PCR | Women | Female | 241 | 67 | 27.8% |
| **A. Bernier** | [35] | 2020 | Russia | PCR | FSW | Female | 385 | 54 | 13.9% |
| **C. Bolumburu** | [41] | 2020 | Spain | PCR | Patients | Female | 17265 | 138 | 0.8% |
| **S. Y. Chan** | [60] | 2020 | UK | PCR | Women | Female | 124 | 37 | 30% |
| **R. Chetty** | [64] | 2020 | South Africa | PCR | Pregnant women | Female | 362 | 47 | 12.9% |
| **H. Z. Hezarjaribi** | [157] | 2020 | Iran | PCR | Patients | Female | 2000 | 11 | 0.55% |
| **C. L. Jasumback** | [172] | 2020 | Eswatini | PCR | Women | Female | 32 | 8 | 25% |
| **N. C. A. Juliana** | [175] | 2020 | Tanzania | PCR | Pregnant women | Female | 493 | 35 | 7.1% |
| **A. U. Kalsom** | [181] | 2020 | Malaysia | PCR | Women | Female | 137 | 1 | 0.7% |
| **H. K. Kareem** | [185] | 2020 | Iraq | PCR | T2DM women | Female | 110 | 27 | 24.5% |
| **A. Medina-Marino** | [251] | 2020 | South Africa | PCR | HIV-infected pregnant women | Female | 427 | 11 | 11.7% |
| **S. Nikpay** | [288] | 2020 | Iran | PCR | Patients | Female | 481 | 7 | 1.5% |
| **D. C. Nyemba** | [295] | 2020 | South Africa | PCR | Pregnant women | Female | 669 | 120 | 18% |
| **M. Rajabpour** | [329] | 2020 | Iran | PCR | Patients | Female | 360 | 30 | 8.3% |
| **B. S. West** | [410] | 2020 | Mexico | PCR | FSW | Female | 584 | 196 | 33.6% |
| **T. Yuh** | [418] | 2020 | Kenya | PCR | Adolescents | Female | 373 | 3 | 0.8% |
| **J. M. Elkins** | [105] | 2020 | USA | PCR | Patients | Female | 75000 | 1802 | 10.9% |
| **E. Fuchs** | [118] | 2020 | USA | PCR | Pregnant women | Female | 739 | 27 | 3.7% |
| **E. M. Keizur** | [188] | 2020 | USA | PCR | Women | Female | 793 | 264 | 33.2% |
| **M. S. Nolan** | [289] | 2020 | USA | PCR | Adolescents | Female | 347 | 45 | 13% |
| **Cheeks, M. L.** | [61] | 2021 | USA | ND | Women | Female | 593 | 59 | 9.9% |
| **S. H. Huang** | [67] | 2020 | China | PCR | Women | Female | 172 | 37 | 21.5% |
| **L. Su** | [368] | 2020 | China | PCR | Women | Female | 487 | 94 | 19.3% |
| **Lin, K. Y.** | [224] | 2021 | China | PCR | Men | Male | 430 | 1 | 0.2% |
| **Chiu, S. F.** | [67] | 2021 | China | Cobas TV assay and DNA Extraction | Patients | Female | 318 | 7 | 2.2% |
| **BROOKE WEBB** | [409] | 2021 | Australi | ND | ND | ND | 7155 | 123 | 1.7% |
| **Nijhuis, R. H. T.** | [287] | 2021 | Netherlands | PCR | Women | Female | 2592 | 10 | 0.4% |
| **Cowley, G.** | [72] | 2021 | Guinea Bissau | PCR | People | Female | 283 | 28 | 9.9% |
| **F a y e m i w o** | [108] | 2021 | UK | ND | ND | Male | 195 | 0 | 0 |
|  |  |  |  |  |  |  | 222 | 36 | 16.2% |
| **Juliana, N.** | [176] | 2021 | South Africa | Molecular assays | Women | Female | 90 | 6 | 7.1% |
| **Fayemiwo,S.** | [108] | 2021 | Nigeria | Cobas TV assay | Women | Female | 234 | 63 | 26.9% |
| **Ghallab, M. M. I.** | [131] | 2021 | Egypt | ND | Women | Female | 222 | 36 | 16.2% |
| **Dalimi, A.** | [80] | 2021 | Iran | PCR | Men | Male | 47 | 31 | 69.5% |
| **Sangaré, I.** | [346] | 2021 | South Africa | ND | Pregnant women | Female | 315 | 10 | 3.2% |
| **Tchankoni, M. K.** | [375] | 2021 | Togo | ND | Sex workers | Female | 310 | 20 | 6.5% |
| **Yasin,** | [417] | 2021 | Ethiopia | Swab | Women | Female | 214 | 7 | 3.3% |
| **[Leonie Toboso Silgo](https://sciprofiles.com/profile/1892512)** | [380] | 2021 | Spain | ND | Pregnant women | Female | 375 | 3 | 0.8% |
| **Sayuri Herath** | [155] | 2021 | Sri Lanka | Direct microscopy, Culture methods and PCR | Patients | ND | 385 | 17 | 4.4% |
| **Isara, A.** | [169] | 2021 | Gambia | DFA | Pregnant women | Female | 280 | 11 | 3.9% |
| **Op de Coul, E. L. M.** | [302] | 2021 | Netherlands | PCR | Pregnant women | Female | 548 | 2 | 0.4% |
| **Huda Abdullah** | [4] | 2022 | Iraq | microscopy | Women | Female | 350 | 100 | 28.57% |
| **Maryam Alikhani** | [14] | 2022 | Iran | PCR | Women | Female | 1765 | 21 | 1.18% |
| **Navaratine , L.** | [276] | 2022 | UK | Cobas TV assay | Women | Female | 183 | 5 | 2.5% |
| **Perry, M. D.** | [319] | 2022 | UK | ND | ND | ND | 2795 | 28 | 1.0% |
| **Zhu, X. W.** | [424] | 2023 | China | Direct microscopy | Patients | Female | 406 | 85 | 20.94% |
| **Kridin , K.** | [207] | 2023 | Israel | PCR | Patients | Female | 841 | 1 | 1.4% |

**ND:** Not determined.

References

1. Abbai NS, Wand H, Ramjee G. 2013. Sexually Transmitted Infections in Women Participating in a Biomedical Intervention Trial in Durban: Prevalence, Coinfections, and Risk Factors. J Sex Transm Dis, 2013, 358402.

2. Abdelaziz ZA, Ibrahim ME, Bilal NE, Hamid ME. 2014. Vaginal infections among pregnant women at Omdurman Maternity Hospital in Khartoum, Sudan. J Infect Dev Ctries, 8(4), 490-7.

3. Abdul-Aziz M, Mahdy MAK, Abdul-Ghani R, Alhilali NA, Al-Mujahed LKA, Alabsi SA, Al-Shawish FAM, Alsarari NJM, Bamashmos W, Abdulwali SJH, Al Karawani M, Almikhlafy AA. 2019. Bacterial vaginosis, vulvovaginal candidiasis and trichomonal vaginitis among reproductive-aged women seeking primary healthcare in Sana'a city, Yemen. BMC Infect Dis, 19(1), 879.

4. Abdullah H, Al-Mussawi K. 2022. Role of transforming growth factor beta-1(TGF-?1) in pathogenesis of Trichomonas vaginalis. Journal of Pharmaceutical Negative Results, 13, 2122-2126.

5. Aboyeji AP, Nwabuisi C. 2003. Prevalence of sexually transmitted diseases among pregnant women in Ilorin, Nigeria. Journal of Obstetrics and Gynaecology, 23(6), 637-639.

6. Adeoye GO, Akande AH. 2007. Epidemiology of Trichomonas vaginalis among women in Lagos metropolis, Nigeria. Pak J Biol Sci, 10(13), 2198-201.

7. Afeke I. 2012. Bacterial vaginosis as a risk factor for acquiring sexually transmitted diseases. Tropical Medicine and International Health, 17, 7-8.

8. Agyarko-Poku T, Sarkodie YA, Frempong EH. 2017. Bacterial vaginosis: Leading cause of vaginal discharge among women attending sexually transmitted infection clinic in Kumasi, Ghana. Sexually Transmitted Infections, 93, A75.

9. Ahmadnia E, Kharaghani R, Maleki A, Avazeh A, Mazloomzadeh S, Sedaghatpisheh T, Jalilvand A, Molae B. 2016. Prevalence and Associated Factors of Genital and Sexually Transmitted Infections in Married Women of Iran. Oman Med J, 31(6), 439-445.

10. Akinbo FO, Oronsaye IS. 2017. Trichomonas vaginalis infection among adolescent girls in some secondary schools in Benin City, Edo State, Nigeria. African Journal of Clinical and Experimental Microbiology, 18(4), 223-229.

11. Al-Awadhi R, Al-Ramadan BA, George SS, Sharma PN, Kapila K. 2010. Gynecologic infections seen in cervical smears in Kuwait. Acta Cytologica, 54(1), 50-54.

12. Al-Majidii NKS, Alsaady HAM. 2020. THE PREVALENCE OF TRICHOMONAS VAGINALIS PARASITE AMONG WOMEN IN SOME REGIONS OF MAYSAN PROVINCE. Periodico Tche Quimica, 17(36), 784-797.

13. Alary M, Lowndes CM, Mukenge-Tshibaka L, Gnintoungbé CAB, Bédard E, Geraldo N, Jossou P, Lafia E, Bernier F, Baganizi É, Joly JR, Frost E, Anagonou S. 2003. Sexually transmitted infections in male clients of female sex workers in Benin: Risk factors and reassessment of the leucocyte esterase dipstick for screening of urethral infections. Sexually Transmitted Infections, 79(5), 388-392.

14. Alikhani M, Akhoundi M, Sereno D, Abdi J, Naserifar R, Mahmoudi MR, Mirzaei A. 2022. Molecular characterization of Trichomonas infections in women of Ilam City, southwestern Iran. Parasitology Research, 121(6), 1631-1638.

15. Almeida MS, Argôlo DS, Almeida Júnior JS, Pinheiro MS, Brito AM. 2010. [Trichomoniasis: prevalence in the female gender in 2004-2005 in Sergipe State, Brazil]. Cien Saude Colet, 15 Suppl 1, 1417-21.

16. Aloui D, Trabelsi S, Bouchekoua M, Khaled S. 2015. [Vulvovaginal trichomoniasis: epidemiology, clinical and parasitological characteristics]. Tunis Med, 93(6), 376-80.

17. Alvarez Rodríguez B, Manzanero M, Morales Miranda S. 2013. Results: Of the First HIV prevalence and risk behaviour study among female sex workers, Belize, 2012. Sexually Transmitted Infections, 89.

18. Ambrozio CL, Nagel AS, Jeske S, Bragança GC, Borsuk S, Villela MM. 2016. Trichomonas vaginalis PREVALENCE AND RISK FACTORS FOR WOMEN IN SOUTHERN BRAZIL. Rev Inst Med Trop Sao Paulo, 58, 61.

19. Amindavaa O, Kristensen S, Pak CY, Khalzan D, Chultemsuren B, Randall AS, Mikolon A, Lkhamsuren E, Tulgaa K, Chimeddorj B, Natsag U. 2005. Sexually transmitted infections among pregnant women attending antenatal clinics in Mongolia: Potential impact on the Mongolian HIV epidemic. International Journal of STD and AIDS, 16(2), 153-157.

20. Anderson BL, Cosentino LA, Simhan HN, Hillier SL. 2007. Systemic immune response to Trichomonas vaginalis infection during pregnancy. Sexually Transmitted Diseases, 34(6), 392-396.

21. Anisimova N, Ivanova T, Guschin A, Simonova E, Zakharova A, Novoschilova O, Shakhzadov V, Shevchenko A. 2011. Undiscovered burden of stis in Russia: Current system shortcomings. Sexually Transmitted Infections, 87, A100-A101.

22. Anorlu RI, Fagbenro Beyioku AF, Fagorala T, Abudu OO, Galadanci HS. 2001. Prevalence of trichomonas vaginalis in patients with vaginal discharge in Lagos, Nigeria. Niger Postgrad Med J, 8(4), 183-6.

23. Arbabi M, Fakhrieh Z, Delavari M, Abdoli A. 2014. Prevalence of Trichomonas vaginalis infection in Kashan city, Iran (2012-2013). Iran J Reprod Med, 12(7), 507-12.

24. Arora BB, Maheshwari M, Devgan N, Arora DR. 2014. Prevalence of Trichomoniasis, Vaginal Candidiasis, Genital Herpes, Chlamydiasis, and Actinomycosis among Urban and Rural Women of Haryana, India. J Sex Transm Dis, 2014, 963812.

25. Asmah RH, Agyeman RO, Obeng-Nkrumah N, Blankson H, Awuah-Mensah G, Cham M, Asare L, Ayeh-Kumi PF. 2018. Trichomonas vaginalis infection and the diagnostic significance of detection tests among Ghanaian outpatients. BMC Womens Health, 18(1), 206.

26. Asmah RH, Blankson HNA, Seanefu KA, Obeng-Nkrumah N, Awuah-Mensah G, Cham M, Ayeh-Kumi PF. 2017. Trichomoniasis and associated co-infections of the genital tract among pregnant women presenting at two hospitals in Ghana. BMC Womens Health, 17(1), 130.

27. Badman SG, Causer LM, Guy R, Tabrizi SN, Francis F, Donovan B, Whiley D. 2016. A preliminary evaluation of a new GeneXpert (Gx) molecular point-of-care test for the detection of Trichomonas vaginalis. Sex Transm Infect, 92(5), 350-2.

28. Badman SG, Vallely LM, Toliman P, Kariwiga G, Lote B, Pomat W, Holmer C, Guy R, Luchters S, Morgan C, Garland SM, Tabrizi S, Whiley D, Rogerson SJ, Mola G, Wand H, Donovan B, Causer L, Kaldor J, Vallely A. 2016. A novel point-of-care testing strategy for sexually transmitted infections among pregnant women in high-burden settings: results of a feasibility study in Papua New Guinea. Bmc Infectious Diseases, 16.

29. Bahram A, Hamid B, Zohre T. 2009. Prevalence of bacterial vaginosis and impact of genital hygiene practices in non-pregnant women in Zanjan, Iran. Oman Medical Journal, 24(4), 288-293.

30. Bakar MSA, Parker E, Jaleel H. 2015. A critical comparison of three diagnostic techniques used for the detection of Trichomonas vaginalis in patients attending a sexual health clinic. International Journal of STD and AIDS, 26(11), 41-42.

31. Bankar SM, Powar RM, Patil SA, Kalthur SG. 2012. Prevalence of non-albican candida infection in Maharashtrian women with leucorrhea. Annals of Tropical Medicine and Public Health, 5(2), 119-123.

32. Barbosa MD, de Souza IBA, Schnaufer ECD, da Silva LF, Goncalves CCM, Simionatto S, Marchioro SB. 2020. Prevalence and factors associated with Trichomonas vaginalis infection in indigenous Brazilian women. Plos One, 15(10).

33. Becker M, Stephen J, Moses S, Washington R, Maclean I, Cheang M, Isac S, Ramesh BM, Alary M, Blanchard J. 2010. Etiology and determinants of sexually transmitted infections in Karnataka state, south India. Sex Transm Dis, 37(3), 159-64.

34. Behets F, Andriamiadana J, Rasamilalao D, Ratsimbazafy N, Randrianasolo D, Dallabetta G, Cohen M. 2001. Sexually transmitted infections and associated socio-demographic and behavioural factors in women seeking primary care suggest Madagascar's vulnerability to rapid HIV spread. Trop Med Int Health, 6(3), 202-11.

35. Bernier A, Rumyantseva T, Reques L, Volkova N, Kyburz Y, Maximov O, Derrienic E, Guschin A, Bouscaillou J, Luhmann N, Pataut D. 2020. HIV and other sexually transmitted infections among female sex workers in Moscow (Russia): prevalence and associated risk factors. Sex Transm Infect, 96(8), 601-607.

36. Beverly AL, Venglarik M, Cotton B, Schwebke JR. 1999. Viability of Trichomonas vaginalis in transport medium. J Clin Microbiol, 37(11), 3749-50.

37. Black V, Magooa P, Radebe F, Myers M, Pillay C, Lewis DA. 2008. The detection of urethritis pathogens among patients with the male urethritis syndrome, genital ulcer syndrome and HIV voluntary counselling and testing clients: should South Africa's syndromic management approach be revised? Sexually Transmitted Infections, 84(4), 254-258.

38. Blackwell AL, Thomas PD, Wareham K, Emery SJ. 1993. Health gains from screening for infection of the lower genital tract in women attending for termination of pregnancy. Lancet, 342(8865), 206-10.

39. Bogaerts J, Ahmed J, Akhter N, Begum N, Rahman M, Nahar S, Van Ranst M, Verhaegen J. 2001. Sexually transmitted infections among married women in Dhaka, Bangladesh: Unexpected high prevalence of herpes simplex type 2 infection. Sexually Transmitted Infections, 77(2), 114-119.

40. Boiko I, Golparian D, Krynytska I, Unemo M. 2019. High prevalence of Chlamydia trachomatis, Neisseria gonorrhoeae and particularly Trichomonas vaginalis diagnosed using US FDA-approved Aptima molecular tests and evaluation of conventional routine diagnostic tests in Ternopil, Ukraine. Apmis, 127(9), 627-634.

41. Bolumburu C, Zamora V, Muñoz-Algarra M, Portero-Azorín F, Escario JA, Ibáñez-Escribano A. 2020. Trichomoniasis in a tertiary hospital of Madrid, Spain (2013-2017): prevalence and pregnancy rate, coinfections, metronidazole resistance, and endosymbiosis. Parasitol Res, 119(6), 1915-1923.

42. Bonney LE, Cooper HLF, Caliendo AM, del Rio C, Hunter-Jones J, Swan DF, Rothenberg R, Druss B. 2012. Access to Health Services and Sexually Transmitted Infections in a Cohort of Relocating African American Public Housing Residents: An Association Between Travel Time and Infection. Sexually Transmitted Diseases, 39(2), 116-121.

43. Boon ME, Holloway PA, Breijer H, Bontekoe TR. 2012. Gardnerella, Trichomonas and Candida in cervical smears of 58,904 immigrants participating in the Dutch national cervical screening program. Acta Cytol, 56(3), 242-6.

44. Boon ME, van Ravenswaay Claasen HH, Kok LP. 2002. Urbanization and baseline prevalence of genital infections including Candida, Trichomonas, and human papillomavirus and of a disturbed vaginal ecology as established in the Dutch Cervical Screening Program. Am J Obstet Gynecol, 187(2), 365-9.

45. Bowden FJ, Paterson BA, Mein J, Savage J, Fairley CK, Garland SM, Tabrizi SN. 1999. Estimating the prevalence of Trichomonas vaginalis, Chlamydia trachomatis, Neisseria gonorrhoeae, and human papillomavirus infection in indigenous women in northern Australia. Sexually Transmitted Infections, 75(6), 431-434.

46. Boyer CB, Shafer MAB, Pollack LM, Canchola J, Moncada J, Schachter J. 2006. Sociodemographic markers and behavioral correlates of sexually transmitted infections in a nonclinical sample of adolescent and young adult women. Journal of Infectious Diseases, 194(3), 307-315.

47. Bradshaw CS, Pierce LI, Tabrizi SN, Fairley CK, Garland SM. 2005. Screening injecting drug users for sexually transmitted infections and blood borne viruses using street outreach and self collected sampling. Sexually Transmitted Infections, 81(1), 53-58.

48. Brooks-Smith-Lowe K, Rodrigo S. 2013. Prevalence of bacterial vaginosis in Grenadian women of reproductive age. West Indian Medical Journal, 62(7), 599-603.

49. Brown JL, Sales JM, DiClemente RJ, Salazar LF, Vanable PA, Carey MP, Brown LK, Romer D, Valois RF, Stanton B. 2012. Predicting discordance between self-reports of sexual behavior and incident sexually transmitted infections with African American female adolescents: results from a 4-city study. AIDS Behav, 16(6), 1491-500.

50. Bruce E, Bauai L, Masta A, Rooney PJ, Paniu M, Sapuri M, Keogh L, Kaldor J, Fairley CK. 2011. Effects of periodic presumptive treatment on three bacterial sexually transmissible infections and HIV among female sex workers in Port Moresby, Papua New Guinea. Sexual Health, 8(2), 222-228.

51. Bruins MJ, Damoiseaux RA, Ruijs GJ. 2009. Association between group A beta-haemolytic streptococci and vulvovaginitis in adult women: a case-control study. Eur J Clin Microbiol Infect Dis, 28(8), 1019-21.

52. Bruni MP, da Silveira MF, Stauffert D, Bicca GLD, dos Santos CC, Farias NAD, Golparian D, Unemo M. 2019. Aptima Trichomonas vaginalis assay elucidates significant underdiagnosis of trichomoniasis among women in Brazil according to an observational study. Sexually Transmitted Infections, 95(2), 129-132.

53. Burton AE, Thomas S. 2019. Sexually transmitted infections and preterm birth among Indigenous women of the Northern Territory, Australia: A case-control study. Aust N Z J Obstet Gynaecol, 59(1), 147-153.

54. Caiyan X, Weiyuan Z, Minghui W, Songwen Z. 2012. Prevalence and risk factors of lower genital tract infections among women in Beijing, China. J Obstet Gynaecol Res, 38(1), 310-5.

55. Camporiondo MP, Farchi F, Ciccozzi M, Denaro A, Gallone D, Maracchioni F, Favalli C, Ciotti M. 2016. Detection of HPV and co-infecting pathogens in healthy Italian women by multiplex real-time PCR. Infez Med, 24(1), 12-7.

56. Canchihuaman FA, Carcamo CP, Garcia PJ, Aral SO, Whittington WLH, Hawes SE, Hughes JP, Holmes KK. 2010. Non-monogamy and risk of infection with Chlamydia trachomatis and Trichomonas vaginalis among young adults and their cohabiting partners in Peru. Sexually Transmitted Infections, 86, 37-44.

57. Carrillo-Ávila JA, Serrano-Garcóa ML, Fernández-Parra J, Sorlózano-Puerto A, Navarro-Maró JM, Stensvold CR, Gutiérrez-Fernández J. 2017. Prevalence and genetic diversity of Trichomonas vaginalis in the general population of Granada and co-infections with Gardnerella vaginalis and Candida species. Journal of Medical Microbiology, 66(10), 1436-1442.

58. Chai SJ, Aumakhan B, Barnes M, Jett-Goheen M, Quinn N, Agreda P, Whittle P, Hogan T, Jenkins WD, Rietmeijer CA, Gaydos CA. 2010. Internet-based screening for sexually transmitted infections to reach nonclinic populations in the community: risk factors for infection in men. Sex Transm Dis, 37(12), 756-63.

59. Chalechale A, Karimi I. 2010. The prevalence of Trichomonas vaginalis infection among patients that presented to hospitals in the Kermanshah district of Iran in 2006 and 2007. Turkish Journal of Medical Sciences, 40(6), 971-975.

60. Chan SY, Winchester S, Unal Z. 2020. Very high prevalence of trichomoniasis in a women's prison. International Journal of STD and AIDS, 31(SUPPL 12), 97.

61. Cheeks ML, Schwartz R, Oleson EC, Cohen S, Drey EA, Seidman D. 2021. Offering routine trichomonas vaginalis testing to patients presenting for abortion at an urban hospital-based clinic. Contraception, 103(6), 423-425.

62. Chen XS, Yin YP, Chen LP, Thuy NT, Zhang GY, Shi MQ, Hu LH, Yu YH. 2006. Sexually transmitted infections among pregnant women attending an antenatal clinic in Fuzhou, China. Sex Transm Dis, 33(5), 296-301.

63. Chersich MF, Luchters SMF, Malonza IM, Mwarogo P, King'ola N, Temmerman M. 2007. Heavy episodic drinking among Kenyan female sex workers is associated with unsafe sex, sexual violence and sexually transmitted infections. International Journal of Std & Aids, 18(11), 764-769.

64. Chetty R, Mabaso N, Abbai N. 2020. Genotypic Variation in Trichomonas vaginalis Detected in South African Pregnant Women. Infect Dis Obstet Gynecol, 2020, 1687427.

65. Chiduo M, Theilgaard ZP, Bakari V, Mtatifikolo F, Bygbjerg I, Flanholc L, Gerstoft J, Christiansen CB, Lemnge M, Katzeristein TL. 2012. Prevalence of sexually transmitted infections among women attending antenatal clinics in Tanga, north eastern Tanzania. International Journal of Std & Aids, 23(5), 325-329.

66. Chitneni P, Bwana MB, Owembabazi M, aO'Neil K, Tukwasibwe D, Najjuma A, Kyampire C, Natukynda S, Byamukama A, Mbalibulha Y, Kalyebara PK, Kaida A, Matthews L. 2019. High STI prevalence among HIV-exposed women planning for pregnancy in rural, Southwestern Uganda. Sexually Transmitted Infections, 95, A121.

67. Chiu SF, Huang PJ, Cheng WH, Huang CY, Chu LJ, Lee CC, Lin HC, Chen LC, Lin WN, Tsao CH, Tang P, Yeh YM, Huang KY. 2021. Vaginal Microbiota of the Sexually Transmitted Infections Caused by Chlamydia trachomatis and Trichomonas vaginalis in Women with Vaginitis in Taiwan. Microorganisms, 9(9).

68. Choudhry S, Ramachandran VG, Das S, Bhattacharya SN, Mogha NS. 2010. Pattern of sexually transmitted infections and performance of syndromic management against etiological diagnosis in patients attending the sexually transmitted infection clinic of a tertiary care hospital. Indian Journal of Sexually Transmitted Diseases, 31(2), 104-108.

69. Claeys P, Ismailov R, Rathe S, Jabbarova A, Claeys G, Fonck K, Temmerman M. 2001. Sexually transmitted infections and reproductive health in Azerbaijan. Sexually Transmitted Diseases, 28(7), 372-378.

70. Costa-Lira E, Jacinto AHVL, Silva LM, Napoleão PFR, Barbosa-Filho RAA, Cruz GJS, Astolfi-Filho S, Borborema-Santos CM. 2017. Prevalence of human papillomavirus, Chlamydia trachomatis, and Trichomonas vaginalis infections in Amazonian women with normal and abnormal cytology. Genetics and Molecular Research, 16(2).

71. Cotch MF, Pastorek JG, 2nd, Nugent RP, Hillier SL, Gibbs RS, Martin DH, Eschenbach DA, Edelman R, Carey JC, Regan JA, Krohn MA, Klebanoff MA, Rao AV, Rhoads GG. 1997. Trichomonas vaginalis associated with low birth weight and preterm delivery. The Vaginal Infections and Prematurity Study Group. Sex Transm Dis, 24(6), 353-60.

72. Cowley G, Milne G, Teixeira da Silva E, Nakutum J, Rodrigues A, Vasileva H, Mabey D, Versteeg B, Last A. 2021. Prevalence of and risk factors for curable sexually transmitted infections on Bubaque Island, Guinea Bissau. Sex Transm Infect, 97(1), 51-55.

73. Creswell J, Guardado ME, Lee J, Nieto AI, Kim AA, Monterroso E, Paz-Bailey G. 2012. HIV and STI control in El Salvador: results from an integrated behavioural survey among men who have sex with men. Sex Transm Infect, 88(8), 633-8.

74. Crucitti T, Jespers V, Mulenga C, Khondowe S, Vandepitte J, Buve A. 2011. Non-Sexual Transmission of Trichomonas vaginalis in Adolescent Girls Attending School in Ndola, Zambia. Plos One, 6(1).

75. Crucitti T, Jespers V, Mulenga C, Khondowe S, Vandepitte J, Buvé A. 2011. Non-sexual transmission of Trichomonas vaginalis in adolescent girls attending school in Ndola, Zambia. PLoS ONE, 6(1).

76. Cu-Uvin S, Hogan JW, Warren D, Klein RS, Peipert J, Schuman P, Holmberg S, Anderson J, Schoenbaum E, Vlahov D, Mayer KH. 1999. Prevalence of lower genital tract infections among human immunodeficiency virus (HIV)-seropositive and high-risk HIV-seronegative women. HIV Epidemiology Research Study Group. Clin Infect Dis, 29(5), 1145-50.

77. da Luz Becker D, dos Santos O, Frasson AP, de Vargas Rigo G, Macedo AJ, Tasca T. 2015. High rates of double-stranded RNA viruses and Mycoplasma hominis in Trichomonas vaginalis clinical isolates in South Brazil. Infect Genet Evol, 34, 181-7.

78. Dahab M, Osman E, Koko W. 2010. Transmission patterns of Trichomonas vaginalis among women population in Khartoum State. International Journal of Infectious Diseases, 14, S79.

79. Dai Q, Hu L, Jiang Y, Shi H, Liu J, Zhou W, Shen C, Yang H. 2010. An epidemiological survey of bacterial vaginosis, vulvovaginal candidiasis and trichomoniasis in the Tibetan area of Sichuan Province, China. Eur J Obstet Gynecol Reprod Biol, 150(2), 207-9.

80. Dalimi A, Payameni S. 2021. Trichomonas Vaginalis Infection in Men with High-Risk Sexual Behaviors. Iranian Journal of Parasitology, 16(3), 411-417.

81. Dan M, Kaneti N, Levin D, Poch F, Samra Z. 2003. Vaginitis in a gynecologic practice in Israel: Causes and risk factors. Israel Medical Association Journal, 5(9), 629-632.

82. Das A, Prabhakar P, Narayanan P, Neilsen G, Morineau G, Mehendale S, Risbud A. 2011. Prevalence and incidence of sexually transmitted infections among female sex workers in two cities in India: Implications for STI control strategies. Sexually Transmitted Infections, 87, A333-A334.

83. Das P, Swain T, Mohanty JR, Sinha S, Padhi B, Torondel B, Cumming O, Panda B, Nayak A, Panigrahi P. 2018. Higher vaginal pH in Trichomonas vaginalis infection with intermediate Nugent score in reproductive-age women-a hospital-based cross-sectional study in Odisha, India. Parasitology Research, 117(9), 2735-2742.

84. Datcu R, Gesink D, Mulvad G, Montgomery-Andersen R, Rink E, Koch A, Ahrens P, Jensen JS. 2013. Vaginal microbiome in women from Greenland assessed by microscopy and quantitative PCR. BMC Infectious Diseases, 13(1).

85. Davey DJ, Nyemba D, DiTullio D, Gomba Y, Klausner JD, Myer L. 2019. Prevalence and determinants of sti in HIV+ and HIV pregnant South African women. Topics in Antiviral Medicine, 27(SUPPL 1), 391s-392s.

86. Davey DLJ, Nyemba DC, Gomba Y, Bekker LG, Taleghani S, DiTullio DJ, Shabsovich D, Gorbach PM, Coates TJ, Klausner JD, Myer L. 2019. Prevalence and correlates of sexually transmitted infections in pregnancy in HIV-infected and- uninfected women in Cape Town, South Africa. Plos One, 14(7).

87. Davies SC, Otto B, Partohudoyo S, Chrisnadarmani VAMA, Neilsen GA, Ciaffi L, Patten J, Samson ET, Sutama IN. 2003. Sexually transmitted infections among female sex workers in Kupang, Indonesia: Searching for a screening algorithm to detect cervical gonococcal and chlamydial infections. Sexually Transmitted Diseases, 30(9), 671-679.

88. Davis A, Goddard-Eckrich D, Dasgupta A, El-Bassel N. 2018. Risk factors associated with sexually transmitted infections among women under community supervision in New York City. International Journal of Std & Aids, 29(8), 766-775.

89. De Jongh M, Lekalakala MR, Le Roux M, Hoosen AA. 2010. Risk of having a sexually transmitted infection in women presenting at a termination of pregnancy clinic in Pretoria, South Africa. J Obstet Gynaecol, 30(5), 480-3.

90. de Lima MCL, Albuquerque TV, Neto ACB, Rehn VNC. 2013. Prevalence and independent risk factors for trichomoniasis in women receiving primary health care. Acta Paulista De Enfermagem, 26(4), 331-337.

91. DeHovitz JA, Kelly P, Feldman J, Sierra MF, Clarke L, Bromberg J, Wan JY, Vermund SH, Landesman S. 1994. Sexually transmitted diseases, sexual behavior, and cocaine use in inner- city women. American Journal of Epidemiology, 140(12), 1125-1134.

92. Desai VK, Kosambiya JK, Thakor HG, Umrigar DD, Khandwala BR, Bhuyan KK. 2003. Prevalence of sexually transmitted infections and performance of STI syndromes against aetiological diagnosis, in female sex workers of red light area in Surat, India. Sexually Transmitted Infections, 79(2), 111-115.

93. Dharma Vijaya MN, Umashankar KM, Sudha, Gulab Nagure A, Kavitha G. 2013. Prevalence of the Trichomonas vaginalis infection in a tertiary care hospital in rural Bangalore, Southern India. Journal of Clinical and Diagnostic Research, 7(7), 1401-1403.

94. Di Bartolomeo S, Rodriguez Fermepin M, Sauka DH, de Torres RA. 2002. Prevalence of associated microorganisms in genital discharge, Argentina. Revista de Saude Publica, 36(5), 545-552.

95. Di Bartolomeo S, Rodríguez M, Sauka D, Alberto De Torres R. 2001. Microbiologic profile in symptomatic pregnant women's genital secretions in Gran Buenos Aires, Argentina. Enfermedades infecciosas y microbiología clínica, 19(3), 99-102.

96. Di Bartolomeo S, Rodríguez M, Sauka D, Alberto De Torres R. 2001. [Microbiologic profile in symptomatic pregnant women's genital secretions in Gran Buenos Aires, Argentina]. Enferm Infecc Microbiol Clin, 19(3), 99-102.

97. Diadhiou M, Ba Diallo A, Barry MS, Alavo SC, Mall I, Gassama O, Ndiaye Guèye MD, Ndao Fall A, Gawa E, Gaye Diallo A, Moreau JC. 2019. Prevalence and Risk Factors of Lower Reproductive Tract Infections in Symptomatic Women in Dakar, Senegal. Infect Dis (Auckl), 12, 1178633719851825.

98. Dionne-Odom J, Khan MJ, Jauk VC, Szychowski J, Long DM, Wallace S, Neely C, Fry K, Marrazzo J, Crain M, Tita ATN. 2019. HIV Status and Other Risk Factors for Prevalent and Incident Sexually Transmitted Infection during Pregnancy (2000-2014). Infect Dis Obstet Gynecol, 2019, 6584101.

99. Divekar AA, Gogate AS, Shivkar LK, Gogate S, Badhwar VR. 2000. Disease prevalence in women attending the STD clinic in Mumbai (formerly Bombay), India. International Journal of STD and AIDS, 11(1), 45-48.

100. Djigma F, Ouedraogo C, Sagna T, Ouermi D, Sanogo K, Bisseye C, Kabre A, Pietra V, Simpore J, Nikiema JB, Musumeci S. 2011. HIV-infected women of Burkina Faso: a "reservoir" of mycoplasma infection. Journal of Infection in Developing Countries, 5(3), 176-181.

101. Domeika M, Savicheva A, Shipitsyna E, Chen CY, Skov-Jenssen J, Hjelmevoll O, Ballard R, Unemo M. 2011. International validation of amplisens naat systems manufactured in Russia for diagnosis of Neisseria Gonorrhoeae, chlamydia trachomatis, mycoplasma genitalium and trichomonas vaginalis. Sexually Transmitted Infections, 87, A353-A354.

102. Donders GGG, Ruban K, Depuydt C, Bellen G, Vanden Broeck D, Jonckheere J, Jacquemyn Y. 2019. Treatment Attitudes for Belgian Women With Persistent Trichomonas vaginalis Infection in the VlaResT Study. Clin Infect Dis, 68(9), 1575-1580.

103. Drake AL, Kinuthia J, Matemo D, McClelland RS, Unger J, John-Stewart G. 2013. Prevalence and cofactors for STIs among pregnant adolescents in Western Kenya. Sexually Transmitted Infections, 89.

104. Dunkle KL, Beksinska ME, Rees VH, Ballard RC, Htun Y, Wilson ML. 2005. Risk factors for HIV infection among sex workers in Johannesburg, South Africa. International Journal of STD and AIDS, 16(3), 256-261.

105. Elkins JM, Cantillo-Campos S, Sheele JM. 2020. Frequency of Coinfection on the Vaginal Wet Preparation in the Emergency Department. Cureus, 12(11).

106. Etuketu IM, Mogaji HO, Alabi OM, Adeniran AA, Oluwol AS, Ekpo UF. 2015. Prevalence and risk factors of Trichomonas vaginalis infection among pregnant women receiving antenatal care in Abeokuta, Nigeria. African Journal of Infectious Diseases, 9(2), 51-56.

107. Fallon SA, Pathela P, Mikati T. 2019. Prevalence and Correlates of Trichomonas vaginalis Infection Using the OSOM Rapid Point-of-Care Test Among Women Attending New York City Sexual Health Clinics, May-July 2016. Sex Transm Dis, 46(11), 748-750.

108. Fayemiwo S, Novak-Frazer L, Adewole I, Richardson R. 2021. Asymptomatic trichomoniasis among women of childbearing age in south-western nigeria. Sexually Transmitted Infections, 97(SUPPL 1), A165.

109. Feldblum PJ, Kuyoh M, Omari M, Aryan K, Bwayo JJ, Welsh M. 2000. Baseline STD prevalence in a community intervention trial of the female condom in Kenya. Sexually Transmitted Infections, 76(6), 454-456.

110. Fernando SD, Herath S, Rodrigo C, Rajapakse L. 2012. Clinical features and sociodemographic factors affecting Trichomonas vaginalis infection in women attending a central sexually transmitted diseases clinic in Sri Lanka. Indian J Sex Transm Dis AIDS, 33(1), 25-31.

111. Fernando SD, Herath S, Rodrigo C, Rajapakse S. 2011. Improving diagnosis of trichomonas vaginalis infection in resource limited health care settings in sri lanka. J Glob Infect Dis, 3(4), 324-8.

112. Ferre VM, Ekouevi DK, Gbeasor-Komlanvi FA, Collin G, Le Hingrat Q, Tchounga B, Salou M, Descamps D, Charpentier C, Dagnra AC. 2019. Prevalence of human papillomavirus, human immunodeficiency virus and other sexually transmitted infections among female sex workers in Togo: a national cross-sectional survey. Clinical Microbiology and Infection, 25(12).

113. Fonck K, Kaul R, Kimani J, Keli F, MacDonald KS, Ronald AR, Plummer FA, Kirui P, Bwayo JJ, Ngugi EN, Moses S, Temmerman M. 2000. A randomized, placebo-controlled trial of monthly azithromycin prophylaxis to prevent sexually transmitted infections and HIV-1 in Kenyan sex workers: study design and baseline findings. Int J STD AIDS, 11(12), 804-11.

114. Fotinatos N, Warmington A, Walker T, Pilbeam M. 2008. Trichomonas vaginalis in Vanuatu. Australian Journal of Rural Health, 16(1), 23-27.

115. Francis SC, Ao TT, Vanobberghen FM, Chilongani J, Hashim R, Andreasen A, Watson-Jones D, Changalucha J, Kapiga S, Hayes RJ. 2014. Epidemiology of Curable Sexually Transmitted Infections among Women at Increased Risk for HIV in Northwestern Tanzania: Inadequacy of Syndromic Management. Plos One, 9(7).

116. Frati ER, Fasoli E, Martinelli M, Colzani D, Bianchi S, Carnelli L, Amendola A, Olivani P, Tanzi E. 2017. Sexually Transmitted Infections: A Novel Screening Strategy for Improving Women's Health in Vulnerable Populations. Int J Mol Sci, 18(6).

117. Frohlich JA, Abdool Karim Q, Mashego MM, Sturm AW, Abdool Karim SS. 2007. Opportunities for treating sexually transmitted infections and reducing HIV risk in rural South Africa. J Adv Nurs, 60(4), 377-83.

118. Fuchs E, Dwiggins M, Lokken E, Unger JA, Eckert LO. 2020. Influence of Sexually Transmitted Infections in Pregnant Adolescents on Preterm Birth and Chorioamnionitis. Infect Dis Obstet Gynecol, 2020, 1908392.

119. Fule SR, Fule RP, Tankhiwale NS. 2012. Clinical and laboratory evidence of Trichomonas vaginalis infection among women of reproductive age in rural area. Indian Journal of Medical Microbiology, 30(3), 314-316.

120. Gadoth A, Mvumbi G, Hoff NA, Musene K, Mukadi P, Ashbaugh HR, Doshi RH, Javanbakht M, Gorbach P, Okitolonda-Wemakoy E, Klausner JD, Rimoin AW. 2019. Urogenital schistosomiasis and sexually transmitted coinfections among pregnant women in a schistosome-endemic region of the Democratic Republic of Congo. American Journal of Tropical Medicine and Hygiene, 101(4), 828-836.

121. Gallion HR, Dupree LJ, Scott TA, Arnold DH. 2009. Diagnosis of Trichomonas vaginalis in Female Children and Adolescents Evaluated for Possible Sexual Abuse: A Comparison of the InPouch TV Culture Method and Wet Mount Microscopy. Journal of Pediatric and Adolescent Gynecology, 22(5), 300-305.

122. Gander S, Scholten V, Osswald I, Sutton M, van Wylick R. 2009. Cervical dysplasia and associated risk factors in a juvenile detainee population. J Pediatr Adolesc Gynecol, 22(6), 351-5.

123. Garcia A, Exposto F, Prieto E, Lopes M, Duarte A, Correia Da Silva R. 2004. Association of Trichomonas vaginalis with sociodemographic factors and other STDs among female inmates in Lisbon. International Journal of STD and AIDS, 15(9), 615-618.

124. García PJ, Cárcamo CP, Chiappe M, Holmes KK. 2007. Sexually transmitted and reproductive tract infections in symptomatic clients of pharmacies in Lima, Peru. Sex Transm Infect, 83(2), 142-6.

125. Gare J, Lupiwa T, Suarkia DL, Paniu MM, Wahasoka A, Nivia H, Kono J, Yeka W, Reeder JC, Mgone CS. 2005. High prevalence of sexually transmitted infections among female sex workers in the eastern highlands province of Papua New Guinea: Correlates and recommendations. Sexually Transmitted Diseases, 32(8), 466-473.

126. Garland SM, Tabrizi SN, Chen S, Byambaa C, Davaajav K. 2001. Prevalence of sexually transmitted infections (Neisseria gonorrhoeae, Chlamydia trachomatis, Trichomonas vaginalis and human papillomavirus) in female attendees of a sexually transmitted diseases clinic in Ulaanbaatar, Mongolia. Infectious Diseases in Obstetrics and Gynecology, 9(3), 143-146.

127. Garrett NJ, Osman F, Maharaj B, Naicker N, Gibbs A, Norman E, Samsunder N, Ngobese H, Mitchev N, Singh R, Karim SSA, Kharsany ABM, Mlisana K, Rompalo A, Mindel A. 2018. Beyond syndromic management: Opportunities for diagnosis-based treatment of sexually transmitted infections in low-and middle-income countries. Plos One, 13(4).

128. Garrow SC, Smith DW, Harnett GB. 2002. The diagnosis of chlamydia, gonorrhoea, and trichomonas infections by self obtained low vaginal swabs, in remote northern Australian clinical practice. Sexually Transmitted Infections, 78(4), 278-281.

129. Gatti FAD, Ceolan E, Greco FSR, Santos PC, Klafke GB, de Oliveira GR, Von Groll A, de Martinez AMB, Goncalves CV, Scaini CJ. 2017. The prevalence of trichomoniasis and associated factors among women treated at a university hospital in southern Brazil. Plos One, 12(3).

130. Gaydos CA, Maldeis NE, Hardick A, Hardick J, Quinn TC. 2009. Mycoplasma genitalium compared to chlamydia, gonorrhoea and trichomonas as an aetiological agent of urethritis in men attending STD clinics. Sexually Transmitted Infections, 85(6), 438-440.

131. Ghallab MMI, Alaa D, Morsy SM. 2021. Multiattribute Analysis of Trichomonas vaginalis Diagnostics and Its Correlation with Clinical Complaints and Contraceptive Methods in a Symptomatic Egyptian Cohort. Infect Dis Obstet Gynecol, 2021, 5525095.

132. Ghebremichael M, Paintsil E. 2011. High risk behaviors and sexually transmitted infections among men in Tanzania. AIDS Behav, 15(5), 1026-32.

133. Ghebremichael M, Paintsil E, Larsen U. 2009. Alcohol abuse, sexual risk behaviors, and sexually transmitted infections in women in Moshi urban district, northern Tanzania. Sex Transm Dis, 36(2), 102-7.

134. Ghosh I, Ghosh P, Bharti AC, Mandal R, Biswas J, Basu P. 2012. Prevalence of Human Papillomavirus and Co-Existent Sexually Transmitted Infections among Female Sex Workers, Men having Sex with Men and Injectable Drug abusers from Eastern India. Asian Pacific Journal of Cancer Prevention, 13(3), 799-802.

135. Gill K, Celum C, Breen G, Thomas K, Morton J, Baeten J, Mendel E, Duyver M, Bekker LG. 2019. High prevalence and incidence of curable STIS among young women initiating prep in a township in South Africa. Sexually Transmitted Infections, 95, A205.

136. Ginindza TG, Stefan CD, Tsoka-Gwegweni JM, Dlamini X, Jolly PE, Weiderpass E, Broutet N, Sartorius B. 2017. Prevalence and risk factors associated with sexually transmitted infections (STIs) among women of reproductive age in Swaziland. Infect Agent Cancer, 12, 29.

137. Ginocchio CC, Chapin K, Smith JS, Aslanzadeh J, Snook J, Hill CS, Gaydos CA. 2012. Prevalence of Trichomonas vaginalis and coinfection with Chlamydia trachomatis and Neisseria gonorrhoeae in the United States as determined by the Aptima Trichomonas vaginalis nucleic acid amplification assay. J Clin Microbiol, 50(8), 2601-8.

138. Goins J, Romero LM, De María Hernandez F, Delgado S, Alvarez B, Beteta E, Paz-Bailey G, Morales S. 2011. High prevalence of stis and risk behaviours among persons living with HIV in nicaragua: Missed opportunities for prevention. Sexually Transmitted Infections, 87, A178-A179.

139. Gómez-Rodríguez LDC, Campo-Urbina ML, Ortega-Ariza N, Bettin-Martínez A, Parody-Muñoz A. 2019. PREVALENCE OF POTENTIALLY PATHOGENIC MICROBIOLOGICAL AGENTS IN VAGINAL EXUDATES OF ASYMPTOMATIC PREGNANT WOMEN, BARRANQUILLA, COLOMBIA, 2014-2015. Rev Colomb Obstet Ginecol, 70(1), 49-56.

140. Gondo DC, Duarte MT, da Silva MG, de Lima Parada CM. 2010. Abnormal vaginal flora in low-risk pregnant women cared for by a public health service: prevalence and association with symptoms and findings from gynecological exams. Rev Lat Am Enfermagem, 18(5), 919-27.

141. Goto A, Nguyen QV, Pham NM, Kato K, Cao TP, Le TH, Hoang QK, Le TQ, Nguyen BT, Katsube M, Ishii S, Yasumura S. 2005. Prevalence of and factors associated with reproductive tract infections among pregnant women in ten communes in Nghe An Province, Vietnam. J Epidemiol, 15(5), 163-72.

142. Grama DF, Casarotti LD, Morato M, Silva LS, Mendonca DF, Limongi JE, Viana JD, Cury MC. 2013. Prevalence of Trichomonas vaginalis and risk factors in women treated at public health units in Brazil: a transversal study. Transactions of the Royal Society of Tropical Medicine and Hygiene, 107(9), 584-591.

143. Gratrix J, Plitt S, Turnbull L, Smyczek P, Brandley J, Scarrott R, Naidu P, Bertholet L, Chernesky M, Read R, Singh AE. 2017. Trichomonas vaginalis Prevalence and Correlates in Women and Men Attending STI Clinics in Western Canada. Sexually Transmitted Diseases, 44(10), 627-629.

144. Gratrix J, Plitt S, Turnbull L, Smyczek P, Brandley J, Scarrott R, Naidu P, Parker P, Blore B, Bull A, Shokoples S, Bertholet L, Martin I, Chernesky M, Read R, Singh A. 2017. Prevalence and antibiotic resistance of Mycoplasma genitalium among STI clinic attendees in Western Canada: a cross-sectional analysis. Bmj Open, 7(7).

145. Graves K, Kissinger P, Muzny CA. 2019. Mechanisms of 5-nitroimidazole resistance in trichomonas vaginalis: a systematic review of the literature. American Journal of Obstetrics and Gynecology, 221(6), 680.

146. Gray RH, Kigozi G, Serwadda D, Makumbi F, Nalugoda F, Watya S, Moulton L, Chen MZ, Sewankambo NK, Kiwanuka N, Sempijja V, Lutalo T, Kagayii J, Wabwire-Mangen F, Ridzon R, Bacon M, Wawer MJ. 2009. The effects of male circumcision on female partners' genital tract symptoms and vaginal infections in a randomized trial in Rakai, Uganda. Am J Obstet Gynecol, 200(1), 42.e1-7.

147. Güralp O, Bostancı A, Özerkman Başaran E, Schild-Suhren M, Kaya B. 2019. Evaluation of the prevalence of sexually transmitted bacterial pathogens in Northern Cyprus by nucleic acid amplification tests, and investigation of the relationship between these pathogens and cervicitis. Turk J Obstet Gynecol, 16(4), 242-248.

148. Hagan JE, Dulmaa N. 2007. Risk factors and prevalence of HIV and sexually transmitted infections among low-income female commercial sex workers in Mongolia. Sex Transm Dis, 34(2), 83-7.

149. Harbertson J, Jamerson M, Graf PCF, Kennemur L, House B, Michael NL, Scott P, Hale B. 2018. Population-based Neisseria gonorrhoeae, Chlamydia trachomatis and Trichomonas vaginalis prevalence using discarded, deidentified urine specimens previously collected for drug testing. Sex Transm Infect, 94(2), 123.

150. Hardick A, Hardick J, Wood BJ, Gaydos C. 2006. Comparison between the Gen-Probe transcription-mediated amplification Trichomonas vaginalis research assay and real-time PCR for Trichomonas vaginalis detection using a Roche LightCycler instrument with female self-obtained vaginal swab samples and male urine samples. Journal of Clinical Microbiology, 44(11), 4197-4199.

151. Harijaona V, Ramambason JD, Morisset R, Rasamindrakotroka A, Ravaoarinoro M. 2009. Prevalence of and risk factors for sexually-transmitted infections in hidden female sex workers. Medecine Et Maladies Infectieuses, 39(12), 909-913.

152. Hathorn E, Ng A, Page M, Hodson J, Gaydos C, Ross JDC. 2015. A service evaluation of the Gen-Probe APTIMA nucleic acid amplification test for Trichomonas vaginalis: should it change whom we screen for infection? Sexually Transmitted Infections, 91(2), 81-86.

153. Hawkes S, Collumbien M, Platt L, Lalji N, Rizvi N, Andreasen A, Chow J, Muzaffar R, ur-Rehman H, Siddiqui N, Hasan S, Bokhari A. 2009. HIV and other sexually transmitted infections among men, transgenders and women selling sex in two cities in Pakistan: a cross-sectional prevalence survey. Sex Transm Infect, 85 Suppl 2, ii8-16.

154. Heine RP, Wiesenfeld HC, Sweet RL, Witkin SS. 1997. Polymerase chain reaction analysis of distal vaginal specimens: A less invasive strategy for detection of Trichomonas vaginalis. Clinical Infectious Diseases, 24(5), 985-987.

155. Herath S, Balendran T, Herath A, Iddawela D, Wickramasinghe S. 2021. Comparison of diagnostic methods and analysis of socio-demographic factors associated with Trichomonas vaginalis infection in Sri Lanka. Plos One, 16(10).

156. Hewett PC, Mensch BS, Ribeiro MC, Jones HE, Lippman SA, Montgomery MR, van de Wijgert JH. 2008. Using sexually transmitted infection biomarkers to validate reporting of sexual behavior within a randomized, experimental evaluation of interviewing methods. Am J Epidemiol, 168(2), 202-11.

157. Hezarjaribi HZ, Taghavi M, Saravi KH, Faridnia R, Kalani F, Mardani A, Jorjani O, Hosseinikhah Z, Esboei BR, Gholami M, Fakhar M. 2020. Actin Gene-Based Molecular Typing of Trichomonas vaginalis Clinical Isolates from the North of Iran. Acta Parasitologica, 65(4), 859-864.

158. Hitti J, Nugent R, Boutain D, Gardella C, Hillier SL, Eschenbach DA. 2007. Racial disparity in risk of preterm birth associated with lower genital tract infection. Paediatr Perinat Epidemiol, 21(4), 330-7.

159. Hoffman CM, Mbambazela N, Sithole P, Morré SA, Dubbink JH, Railton J, McIntyre JA, Kock MM, Peters RPH. 2019. Provision of Sexually Transmitted Infection Services in a Mobile Clinic Reveals High Unmet Need in Remote Areas of South Africa: A Cross-sectional Study. Sex Transm Dis, 46(3), 206-212.

160. Hoke TH, Feldblum PJ, Van Damme K, Nasution MD, Grey TW, Wong EL, Ralimamonjy L, Raharimalala L, Rasamindrakotroka A. 2007. Randomised controlled trial of alternative male and female condom promotion strategies targeting sex workers in Madagascar. Sexually Transmitted Infections, 83(6), 448-453.

161. Hoosen AA, Abdul H, Moodley J, Sturm AW. 1996. Sexually transmitted infections in ambulatory patients with pelvic inflammatory disease. Journal of Obstetrics and Gynaecology, 16(6), 544-547.

162. Houso Y, Farraj MA, Ramlawi A, Essawi T. 2011. Detection of Trichomonas vaginalis in Vaginal Swab Clinical Samples from Palestinian Women by Culture. ISRN Microbiol, 2011, 872358.

163. Huppert JS, Batteiger BE, Braslins P, Feldman JA, Hobbs MM, Sankey HZ, Sena AC, Wendel KA. 2005. Use of an immunochromatographic assay for rapid detection of Trichomonas vaginalis in vaginal specimens. Journal of Clinical Microbiology, 43(2), 684-687.

164. Huppert JS, Biro F, Lan D, Mortensen JE, Reed J, Slap GB. 2007. Urinary symptoms in adolescent females: STI or UTI? J Adolesc Health, 40(5), 418-24.

165. Huq M, Chawdhury FA, Mitra DK, Islam MA, Salahuddin G, Das J, Rahman M. 2010. A pilot study on the prevalence of sexually transmitted infections among clients of brothel-based female sex workers in Jessore, Bangladesh. Int J STD AIDS, 21(4), 300-1.

166. Ignacio MAO, Andrade J, Freitas APF, Silva MG, Duarte MTC. 2017. Prevalence of sexually transmitted infections and associated factors in women who have sex with women. Sexually Transmitted Infections, 93, A148.

167. Ijasan O, Okunade KS, Oluwole AA. 2018. The prevalence and risk factors for Trichomonas vaginalis infection amongst human immunodeficiency virus-infected pregnant women attending the antenatal clinics of a university teaching hospital in Lagos, South-Western, Nigeria. Niger Postgrad Med J, 25(1), 21-26.

168. Ingabire R, Parker R, Nyombayire J, Ko JE, Mukamuyango J, Bizimana J, Price MA, Laufer D, Tichacek A, Wall K, Allen S, Karita E. 2019. Female sex workers in Kigali, Rwanda: a key population at risk of HIV, sexually transmitted infections, and unplanned pregnancy. Int J STD AIDS, 30(6), 557-568.

169. Isara A, Baldeh AK. 2021. Prevalence of sexually transmitted infections among pregnant women attending antenatal clinics in West Coast Region of The Gambia. Afr Health Sci, 21(2), 585-592.

170. Jackson EJ, Rakwar JP, Chohan B, Mandaliya K, Bwayo JJ, NdinyaAchola JO, Nagelkerke NJD, Kreiss JK, Moses S. 1997. Urethral infection in a workplace population of East African men: Evaluation of strategies for screening and management. Journal of Infectious Diseases, 175(4), 833-838.

171. Jansen K, Bremer V, Steffen G, Sarma N, Munstermann D, Lucht A, Tiemann C. 2013. PREVALENCE OF GENITAL INFECTIONS WITH CHLAMYDIA TRACHOMATIS (CT), NEISSERIA GONORRHOEA (NG) AND TRICHOMONAS VAGINALIS (TV) IN HARD-TO-REACH FEMALE SEX WORKERS IN NORTH RHINE-WESTPHALIA, GERMANY: THE STI-OUTREACH STUDY. Sexually Transmitted Infections, 89, A202-A203.

172. Jasumback CL, Perry SH, Ness TE, Matsenjwa M, Masangane ZT, Mavimbela M, Mthethwa N, Dlamini L, Mphaya J, Kirchner HL, Mandalakas A, Kay AW. 2020. Point-of-Care testing to guide treatment and estimate risk factors for sexually transmitted infections in adolescents and young people with human immunodeficiency virus in Eswatini. Open Forum Infectious Diseases, 7(3), 1-9.

173. Javanbakht M, Stirland A, Stahlman S, Smith LV, Chien M, Torres R, Guerry S. 2013. Prevalence and factors associated with Trichomonas vaginalis infection among high-risk women in Los Angeles. Sex Transm Dis, 40(10), 804-7.

174. Joesoef MR, Valleroy LA, Kuntjoro TM, Kamboji A, Linnan M, Barakbah Y, Idajadi A, St Louis ME. 1998. Risk profile of female sex workers who participate in a routine penicillin prophylaxis programme in Surabaya, Indonesia. Int J STD AIDS, 9(12), 756-60.

175. Juliana NCA, Deb S, Ouburg S, Chauhan A, Pleijster J, Ali SM, Morré SA, Sazawal S, Ambrosino E. 2020. The Prevalence of Chlamydia trachomatis and Three Other Non-Viral Sexually Transmitted Infections among Pregnant Women in Pemba Island Tanzania. Pathogens, 9(8).

176. Juliana NCA, Omar AM, Pleijster J, Aftab F, Uijldert NB, Ali SM, Ouburg S, Sazawal S, Morre SA, Deb S, Ambrosino E. 2021. The Natural Course of Chlamydia trachomatis, Neisseria gonorrhoeae, Trichomonas vaginalis, and Mycoplasma genitalium in Pregnant and Post-Delivery Women in Pemba Island, Tanzania. Microorganisms, 9(6).

177. Kabakchieva E, Vassileva S, Kelly JA, Amirkhanian YA, Difranceisco WJ, McAuliffe TL, Antonova R, Mihaylova M, Vassilev B, Khoursine R, Petrova E. 2006. HIV risk behavior patterns, predictors, and sexually transmitted disease prevalence in the social networks of young Roma (Gypsy) men in Sofia, Bulgaria. Sexually Transmitted Diseases, 33(8), 485-490.

178. Kaida A, Dietrich JJ, Laher F, Beksinska M, Jaggernath M, Bardsley M, Smith P, Cotton L, Chitneni P, Closson K, Lewis DA, Smit JA, Ndung'u T, Brockman M, Gray G. 2018. A high burden of asymptomatic genital tract infections undermines the syndromic management approach among adolescents and young adults in South Africa: implications for HIV prevention efforts. BMC Infect Dis, 18(1), 499.

179. Kalantari N, Ghaffari S, Bayani M. 2014. Trichomonas, Candida, and Gardnerella in cervical smears of Iranian women for cancer screening. North American Journal of Medical Sciences, 6(1), 25-29.

180. Kalantari N, Ghaffari S, Esmaeilzadeh S. 2012. The frequency study of trichomoniasis in women referred to gynecology clinic of Ayatollah Rohani Hospital, Babol, Iran, in 2010. Annals of Tropical Medicine and Public Health, 5(5), 498-501.

181. Kalsom AU, Suvra B, Zainul RMR, Norlia OS, Zalina I, Anita S. 2020. Prevalence Rates of Chlamydia Trachomatis and Other Sexually Transmitted Organisms in Infertile Couples Attending a Tertiary Medical Centre in Malaysia. Iium Medical Journal Malaysia, 19(1), 61-74.

182. Kamara P, Hylton-Kong T, Brathwaite A, Del Rosario GR, Kristensen S, Patrick N, Weiss H, Figueroa PJ, Vermund SH, Jolly PE. 2000. Vaginal infections in pregnant women in Jamaica: prevalence and risk factors. Int J STD AIDS, 11(8), 516-20.

183. Kapina M, Reid C, Roman K, Cyrus-Cameron E, Kwiecien A, Weiss S, Vermund SH. 2009. HIV incidence rates and risk factors for urban women in Zambia: Preparing for a microbicide clinical trial. Sexually Transmitted Diseases, 36(3), 129-133.

184. Karabulut A, Alan T, Ali Ekiz M, Iritaş A, Kesen Z, Yahşi S. 2010. Evaluation of cervical screening results in a population at normal risk. Int J Gynaecol Obstet, 110(1), 40-2.

185. Kareem HK, Hamad MM, Hasan MA, Abd alsammed MA. 2020. Study on Trichomonas vaginalis infection in women with type-2 diabetes mellitus and vaginal discharge in Thi-Qar Government. European Journal of Molecular and Clinical Medicine, 7(8), 4471-4478.

186. Karou SD, Sanou D, Ouermi D, Pignatelli S, Pietra V, Moret R, Nikiema JB, Simpore J. 2011. Enteric parasites prevalence at Saint Camille Medical Centre in Ouagadougou, Burkina Faso. Asian Pac J Trop Med, 4(5), 401-3.

187. Kaul P, Gupta I, Sehgal R, Malla N. 2004. Trichomonas vaginalis: random amplified polymorphic DNA analysis of isolates from symptomatic and asymptomatic women in India. Parasitology International, 53(3), 255-262.

188. Keizur EM, Bristow CC, Baik Y, Klausner JD. 2020. Knowledge and testing preferences for Chlamydia trachomatis, Neisseria gonorrhoeae, and Trichomonas vaginalis infections among female undergraduate students. J Am Coll Health, 68(7), 754-761.

189. Kenyon CR, Buyze J, Klebanoff M, Brotman RM. 2017. Incident Trichomonas vaginalis Is Associated With Partnership Concurrency: A Longitudinal Cohort Study. Sex Transm Dis, 44(11), 695-699.

190. Kerubo E, Laserson KF, Otecko N, Odhiambo C, Mason L, Nyothach E, Oruko KO, Bauman A, Vulule J, Zeh C, Phillips-Howard PA. 2016. Prevalence of reproductive tract infections and the predictive value of girls' symptom-based reporting: findings from a cross-sectional survey in rural western Kenya. Sex Transm Infect, 92(4), 251-6.

191. Khan MS, Unemo M, Zaman S, Lundborg CS. 2011. HIV, STI prevalence and risk behaviours among women selling sex in Lahore, Pakistan. Bmc Infectious Diseases, 11.

192. Khan Z, Bhargava A, Mittal P, Bharti R, Puri P, Khunger N, Bala M. 2019. Evaluation of reliability of self-collected vaginal swabs over physician-collected samples for diagnosis of bacterial vaginosis, candidiasis and trichomoniasis, in a resource-limited setting: a cross-sectional study in India. BMJ Open, 9(8), e025013.

193. Khatib N, Bradbury C, Chalker V, Koh GC, Smit E, Wilson S, Watson J. 2015. Prevalence of Trichomonas vaginalis, Mycoplasma genitalium and Ureaplasma urealyticum in men with urethritis attending an urban sexual health clinic. Int J STD AIDS, 26(6), 388-92.

194. Kim AA, Sun LP, Chhorvann C, Lindan C, Van Griensven F, Kilmarx PH, Sirivongrangson P, Louie JK, Leng HB, Page-Shafer K. 2005. High prevalence of HIV and sexually transmitted infections among indirect sex workers in Cambodia. Sex Transm Dis, 32(12), 745-51.

195. Kim HJ, Park JK, Park SC, Kim YG, Choi H, Ko JI, Kim MK, Jeong YB, Shin YS. 2017. The prevalence of causative organisms of community-acquired urethritis in an age group at high risk for sexually transmitted infections in Korean Soldiers. Journal of the Royal Army Medical Corps, 163(1), 20-22.

196. Kim SJ, Lee DS, Lee SJ. 2011. The Prevalence and Clinical Significance of Urethritis and Cervicitis in Asymptomatic People by Use of Multiplex Polymerase Chain Reaction. Korean Journal of Urology, 52(10), 703-708.

197. Kim TH, Kim CS, Choe HS, Lee DS, Hong SH, Lee SJ, Han CH, Cho YH, Lee MK. 2013. The prevalence of sexually transmitted infections detected by nucleic acid amplification tests in symptomatic patients and asymptomatic volunteers. International Journal of Antimicrobial Agents, 42, S110.

198. Kim Y, Kim J, Lee KA. 2014. Prevalence of sexually transmitted infections among healthy Korean women: Implications of multiplex PCR pathogen detection on antibiotic therapy. Journal of Infection and Chemotherapy, 20(1), 74-76.

199. Kim Y, Kim J, Lee KA. 2015. Analytical Performance of Multiplex Real-Time PCR for Six Sexually Transmitted Pathogens. Clinical Laboratory, 61(11), 1749-1754.

200. Kissinger PJ, Dumestre J, Clark RA, Wenthold L, Mohammed H, Hagensee ME, Martin DH. 2005. Vaginal swabs versus lavage for detection of Trichomonas vaginalis and bacterial vaginosis among HIV-positive women. Sexually Transmitted Diseases, 32(4), 227-230.

201. Kiweewa FM, Brown E, Mishra A, Nair G, Palanee-Phillips T, Mgodi N, Nakabiito C, Chakhtoura N, Hillier SL, Baeten JM, Soto-Torres L, Schwartz K, Makanani B, Martinson F, Bekker LG, Govender V, Mhlanga F. 2019. Acquisition of Sexually Transmitted Infections among Women Using a Variety of Contraceptive Options: A prospective Study among High-risk African Women. Journal of the International AIDS Society, 22(2).

202. Klavs I, Milavec M, Kustec T, Grgič Vitek M, Lavtar D, Zaletel M, Golle A, Duh D, Čretnik TŽ. 2019. Prevalence of chlamydia, Gonorrhoea, M. Genitalium and T. Vaginalis in the general population of Slovenia, 2016-2017. Sexually Transmitted Infections, 95, A187.

203. Knox J, Tabrizi SN, Miller P, Petoumenos K, Law M, Chen SJ, Garland SM. 2002. Evaluation of self-collected samples in contrast to practitioner collected samples for detection of Chlamydia trachomatis, Neisseria gonorrhoeae, and Trichomonas vaginalis by polymerase chain reaction among women living in remote areas. Sexually Transmitted Diseases, 29(11), 647-654.

204. Konadu DG, Owusu-Ofori A, Yidana Z, Boadu F, Iddrisu LF, Adu-Gyasi D, Dosoo D, Awuley RL, Owusu-Agyei S, Asante KP. 2019. Prevalence of vulvovaginal candidiasis, bacterial vaginosis and trichomoniasis in pregnant women attending antenatal clinic in the middle belt of Ghana. BMC Pregnancy and Childbirth, 19(1).

205. Korycińska J, Dzika E, Waśniewski T, Lepczyńska M, Kubiak K. 2017. The prevalence of Trichomonas vaginalis infections in the population of Warmińsko-Mazurskie voivodeship (North-Eastern Poland). Przegl Epidemiol, 71(4), 547-554.

206. Kosambiya JK, Desai VK, Bhardwaj P, Chakraborty T. 2009. RTI/STI prevalence among urban and rural women of Surat: A community-based study. Indian J Sex Transm Dis AIDS, 30(2), 89-93.

207. Kridin K, Ingram B, Becker D, Shiloah N, Azrad M, Habib S, Peretz A. 2023. Sexually Transmitted Diseases in Northern Israel: Insights From a Large Referral Laboratory. J Low Genit Tract Dis, 27(1), 51-55.

208. Kumarasamy N, Balakrishnan P, Venkatesh KK, Srikrishnan AK, Cecelia AJ, Thamburaj E, Solomon S, Mayer KH. 2008. Prevalence and incidence of sexually transmitted infections among South Indians at increased risk of HIV infection. Aids Patient Care and Stds, 22(8), 677-682.

209. Kurewa NE, Mapingure MP, Munjoma MW, Chirenje MZ, Rusakaniko S, Stray-Pedersen B. 2010. The burden and risk factors of Sexually Transmitted Infections and Reproductive Tract Infections among pregnant women in Zimbabwe. BMC Infect Dis, 10, 127.

210. Kwon I, McNulty A, Read P. 2013. The prevalence of Trichomonas vaginalis detected by wet mount and polymerase chain reaction in Sydney women. Sex Health, 10(4), 385-6.

211. Lafort Y, Sawadogo Y, Delvaux T, Vuylsteke B, Laga M. 2003. Should family planning clinics provide clinical services for sexually transmitted infections? A case study from Cote d'Ivoire. Tropical Medicine & International Health, 8(6), 552-560.

212. Landes M, Thorne C, Barlow P, Fiore S, Malyuta R, Martinelli P, Posokhova S, Savasi V, Semenenko I, Stelmah A, Tibaldi C, Newell ML. 2007. Prevalence of sexually transmitted infections in HIV-1 infected pregnant women in Europe. European Journal of Epidemiology, 22(12), 925-936.

213. Laurent C, Seck K, Coumba N, Kane T, Samb N, Wade A, Liégeois F, Mboup S, Ndoye I, Delaporte E. 2003. Prevalence of HIV and other sexually transmitted infections, and risk behaviours in unregistered sex workers in Dakar, Senegal. Aids, 17(12), 1811-6.

214. Lazenby GB, Taylor PT, Badman BS, McHaki E, Korte JE, Soper DE, Young Pierce J. 2014. An association between Trichomonas vaginalis and high-risk human papillomavirus in rural Tanzanian women undergoing cervical cancer screening. Clin Ther, 36(1), 38-45.

215. Le PT, Hamasuna R, Matsumoto M, Furubayashi K, Hatanaka M, Kawai S, Yamaguchi T, Uehara K, Murakami N, Yoshioka M, Nakayama K, Shiono Y, Muraoka K, Suzuki M, Fujimoto N, Matsumoto T. 2017. The detection of microorganisms related to urethritis from the oral cavity of male patients with urethritis. Journal of Infection and Chemotherapy, 23(10), 668-673.

216. Le Roux MC, Ramoncha MR, Adam A, Hoosen AA. 2010. Aetiological agents of urethritis in symptomatic South African men attending a family practice. Int J STD AIDS, 21(7), 477-81.

217. Ledru S, Meda N, Fofana M, Soula G, Bazié AJ, Chiron JP. 1996. Etiologic study of genitourinary infections in women of childbearing age in Bobo-Dioulasso, Burkina Faso, 1992. Sex Transm Dis, 23(2), 151-6.

218. Lee A, Park J, Kim D, Cho A, Kim Y, Lee K. 2016. The prevalence of six common sexually transmitted pathogens by multiplex real-time PCR in cervico-vaginal specimens collected from sexually active women in Korea. Clinical Chemistry, 62(10), S140.

219. Lee J, Seo YB, Park JJ, Jeong SK. 2016. The evaluation and risk assessment of sexually transmitted disease in Korean adolescents at risk. International Journal of Infectious Diseases, 45, 202.

220. Leon SR, Konda KA, Bernstein KT, Pajuelo JB, Rosasco AM, Caceres CF, Coates TJ, Klausner JD. 2009. Trichomonas vaginalis infection and associated risk factors in a socially-Marginalized female population in coastal peru. Infectious Diseases in Obstetrics and Gynecology, 2009.

221. Leutscher P, Jensen JS, Hoffmann S, Berthelsen L, Ramarakoto CE, Ramaniraka V, Randrianasolo B, Raharisolo C, Böttiger B, Rousset D, Grosjean P, McGrath MM, Christensen N, Migliani R. 2005. Sexually transmitted infections in rural Madagascar at an early stage of the HIV epidemic: a 6-month community-based follow-up study. Sex Transm Dis, 32(3), 150-5.

222. Leutscher PDC, Pedersen M, Raharisolo C, Jensen JS, Hoffmann S, Lisse I, Ostrowski SR, Reimert CM, Mauclere P, Ullum H. 2005. Increased prevalence of leukocytes and elevated cytokine levels in semen from Schistosoma haematobium-infected individuals. Journal of Infectious Diseases, 191(10), 1639-1647.

223. Lewis DA, Chirwa TF, Msimang VMY, Radebe FM, Kamb ML, Firnhaber CS. 2012. Urethritis/cervicitis pathogen prevalence and associated risk factors among asymptomatic HIV-infected patients in South Africa. Sexually Transmitted Diseases.

224. Lin KY, Sun HY, Lee TF, Chuang YC, Wu UI, Liu WC, Chang SY, Chen YJ, Hung CC, Chang SC. 2021. High prevalence of sexually transmitted coinfections among at-risk people living with HIV. Journal of the Formosan Medical Association, 120(10), 1876-1883.

225. Lobo TT, Feijo G, Carvalho SE, Costa PL, Chagas C, Xavier J, Simões-Barbosa A. 2003. A comparative evaluation of the Papanicolaou test for the diagnosis of trichomoniasis. Sexually Transmitted Diseases, 30(9), 694-699.

226. Lockhart A, Psioda M, Ting J, Campbell S, Mugo N, Kwatampora J, Chitwa M, Kimani J, Gakure A, Smith JS. 2018. Prospective Evaluation of Cervicovaginal Self- and Cervical Physician Collection for the Detection of Chlamydia trachomatis, Neisseria gonorrhoeae, Trichomonas vaginalis, and Mycoplasma genitalium Infections. Sexually Transmitted Diseases, 45(7), 488-493.

227. Lockhart A, Senkomago V, Ting J, Chitwa M, Kimani J, Gakure H, Kwatampora J, Patel S, Mugo N, Smith JS. 2019. Prevalence and Risk Factors of Trichomonas vaginalis Among Female Sexual Workers in Nairobi, Kenya. Sexually Transmitted Diseases, 46(7), 458-464.

228. Lowe S, Mudzviti T, Mandiriri A, Shamu T, Mudhokwani P, Chimbetete C, Luethy R, Pascoe M. 2019. Sexually transmitted infections, the silent partner i n HIV-infected women in Zimbabwe. Southern African Journal of Hiv Medicine, 20(1).

229. Luo L, Xu JJ, Wang GX, Ding GW, Wang N, Wang HB. 2016. Vaginal douching and association with sexually transmitted infections among female sex workers in a prefecture of Yunnan Province, China. International Journal of STD and AIDS, 27(7), 560-567.

230. Luppi CG, de Oliveira RLS, Veras MA, Lippman SA, Jones H, de Jesus CH, Pinho AA, Ribeiro MC, Caiaffa-Filho H. 2011. Early diagnosis and correlations of sexually transmitted infections among women in primary care health services. Revista Brasileira de Epidemiologia, 14(3), 467-477.

231. Lusk MJ, Naing Z, Rayner B, Rismanto N, McLver CJ, Cumming RG, McGeechan K, Rawlinson WD, Konecny P. 2010. Trichomonas vaginalis: underdiagnosis in urban Australia could facilitate re-emergence. Sexually Transmitted Infections, 86(3), 227-230.

232. Mabonga E, Taylor C, Gaydos C, Manabe Y, Kisakye J, Parkes-Ratanshi R. 2016. Prevalence of STIs in HIV-infected pregnant women in Uganda. HIV Medicine, 17, 71.

233. Madhivanan P, Bartman MT, Pasutti L, Krupp K, Arun A, Reingold AL, Klausner JD. 2009. Prevalence of Trichomonas vaginalis infection among young reproductive age women in India: Implications for treatment and prevention. Sexual Health, 6(4), 339-344.

234. Madhivanan P, Bartman MT, Pasutti L, Krupp K, Arun A, Reingold AL, Klausner JD. 2009. Prevalence of Trichomonas vaginalis infection among young reproductive age women in India: implications for treatment and prevention. Sex Health, 6(4), 339-44.

235. Madhivanan P, Li T, Trammell S, Desai C, Srinivas V, Arun A, Klausner JD, Krupp K. 2013. Performance of the OSOM Trichomonas Rapid Test for diagnosis of Trichomonas vaginalis infection among women in Mysore, India. Sexual Health, 10(4), 320-324.

236. Madico G, Quinn TC, Rompalo A, McKee KT, Gaydos CA. 1998. Diagnosis of Trichomonas vaginalis infection by PCR using vaginal swab samples. Journal of Clinical Microbiology, 36(11), 3205-3210.

237. Maghsoudi R, Danesh A, Kabiri N, Setorki M, Doudi M. 2014. Prevalence of the genital tract bacterial infections after vaginal reconstructive surgery. Pak J Biol Sci, 17(9), 1058-63.

238. Mahdi NK, Gany ZH, Sharief M. 2001. Risk factors for vaginal trichomoniasis among women in Basra, Iraq. Eastern Mediterranean health journal = La revue de santé de la Méditerranée orientale = al-Majallah al-ihhīyah li-sharq al-mutawassi, 7(6), 918-924.

239. Mahmoud A, Sherif NA, Abdella R, El-Genedy AR, El Kateb AY, Askalani AN. 2015. Prevalence of Trichomonas vaginalis infection among Egyptian women using culture and Latex agglutination: cross-sectional study. BMC Womens Health, 15, 7.

240. Mahto M, Zia S. 2008. Measuring the gap: From Home Office to the National Health Service in the provision of a one-stop shop sexual health service in a female prison in the UK. International Journal of STD and AIDS, 19(9), 586-589.

241. Maina AN, Kimani J, Anzala O. 2016. Prevalence and risk factors of three curable sexually transmitted infections among women in Nairobi, Kenya. BMC Res Notes, 9, 193.

242. Månsson F, Camara C, Biai A, Monteiro M, da Silva ZJ, Dias F, Alves A, Andersson S, Fenyö EM, Norrgren H, Unemo M. 2010. High prevalence of HIV-1, HIV-2 and other sexually transmitted infections among women attending two sexual health clinics in Bissau, Guinea-Bissau, West Africa. Int J STD AIDS, 21(9), 631-5.

243. Mascarenhas RE, Machado MS, Costa e Silva BF, Pimentel RF, Ferreira TT, Leoni FM, Grassi MF. 2012. Prevalence and risk factors for bacterial vaginosis and other vulvovaginitis in a population of sexually active adolescents from Salvador, Bahia, Brazil. Infect Dis Obstet Gynecol, 2012, 378640.

244. Masha SC, Cools P, Crucitti T, Sanders EJ, Vaneechoutte M. 2017. Molecular typing of Trichomonas vaginalis isolates by actin gene sequence analysis and carriage of T. vaginalis viruses. Parasit Vectors, 10(1), 537.

245. Matini M, Rezaei H, Fallah M, Maghsood AH, Saidijam M, Shamsi-Ehsan T. 2017. Genotyping, Drug Susceptibility and Prevalence Survey of Tri-chomonas vaginalis among Women Attending Gynecology Clinics in Hamadan, Western Iran, in 2014-2015. Iranian Journal of Parasitology, 12(1), 29-37.

246. Mawu FO, Davies SC, McKechnie M, Sedyaningsih ER, Widihastuti A, Hillman RJ. 2011. Sexually transmissible infections among female sex workers in Manado, Indonesia, using a multiplex polymerase chain reaction-based reverse line blot assay. Sex Health, 8(1), 52-60.

247. Mayer KH, Bush T, Henry K, Overton ET, Hammer J, Richardson J, Wood K, Conley L, Papp J, Caliendo AM, Patel P, Brooks JT. 2012. Ongoing sexually transmitted disease acquisition and risk-taking behavior among US HIV-infected patients in primary care: implications for prevention interventions. Sex Transm Dis, 39(1), 1-7.

248. McCormick DF, Rahman M, Zadrozny S, Alam A, Ashraf L, Neilsen GA, Kelly R, Menezes P, Miller WC, Hoffman IF. 2013. Prevention and control of sexually transmissible infections among hotel-based female sex workers in Dhaka, Bangladesh. Sex Health, 10(6), 478-86.

249. McIver CJ, Rismanto N, Smith C, Naing ZW, Rayner B, Lusk MJ, Konecny P, White PA, Rawlinson WD. 2009. Multiplex PCR Testing Detection of Higher-than-Expected Rates of Cervical Mycoplasma, Ureaplasma, and Trichomonas and Viral Agent Infections in Sexually Active Australian Women. Journal of Clinical Microbiology, 47(5), 1358-1363.

250. Meda N, Ledru S, Fofana M, Lankoande S, Soula G, Bazie AJ, Chiron JP. 1995. SEXUALLY-TRANSMITTED DISEASES AND HUMAN-IMMUNODEFICIENCY-VIRUS INFECTION AMONG WOMEN WITH GENITAL INFECTIONS IN BURKINA-FASO. International Journal of Std & Aids, 6(4), 273-277.

251. Medina-Marino A, Mudau M, Kojima N, Peters RPH, Feucht UD, Vos LD, Olivier D, Muzny CA, McIntyre JA, Klausner JD. 2020. Persistent Chlamydia trachomatis, Neisseria gonorrhoeae or Trichomonas vaginalis positivity after treatment among human immunodeficiency virus-infected pregnant women, South Africa. International Journal of STD and AIDS, 31(4), 294-302.

252. Mehta S, Gaydos C, Maclean I, Odoyo-June E, Moses S, Murugu R, Agunda L, Nyagaya E, Quinn N, Bailey R. 2011. Medical male circumcision may be protective of urogenital mycoplasma genitalium infection: Results from a randomised trial in Kisumu, Kenya. Sexually Transmitted Infections, 87, S218-S219.

253. Mehta SD, Gaydos C, MacLean I, Odoyo-June E, Moses S, Agunda L, Quinn N, Bailey RC. 2012. The effect of medical male circumcision on urogenital mycoplasma genitalium among men in Kisumu, Kenya. Sexually Transmitted Diseases, 39(4), 276-280.

254. Mehta SD, Moses S, Agot K, Parker C, Ndinya-Achola JO, Maclean I, Bailey RC. 2009. Adult Male Circumcision Does Not Reduce the Risk of Incident Neisseria gonorrhoeae, Chlamydia trachomatis, or Trichomonas vaginalis Infection: Results from a Randomized, Controlled Trial in Kenya. Journal of Infectious Diseases, 200(3), 370-378.

255. Mendoza L, Mongelos P, Paez M, Castro A, Rodriguez-Riveros I, Gimenez G, Araujo P, Echagüe G, Diaz V, Laspina F, Castro W, Jimenez R, Marecos R, Ever S, Deluca G, Picconi MA. 2013. Human papillomavirus and other genital infections in indigenous women from Paraguay: A cross-sectional analytical study. BMC Infectious Diseases, 13(1).

256. Menéndez C, Castellsagué X, Renom M, Sacarlal J, Quintó L, Lloveras B, Klaustermeier J, Kornegay JR, Sigauque B, Bosch FX, Alonso PL. 2010. Prevalence and risk factors of sexually transmitted infections and cervical neoplasia in women from a rural area of southern Mozambique. Infect Dis Obstet Gynecol, 2010.

257. Mgone CS, Lupiwa T, Yeka W. 2002. High prevalence of Neisseria gonorrhoeae and multiple sexually transmitted diseases among rural women in the Eastern Highlands Province of Papua New Guinea, detected by polymerase chain reaction. Sex Transm Dis, 29(12), 775-9.

258. Mhlongo S, Magooa P, Muller EE, Nel N, Radebe F, Wasserman E, Lewis DA. 2010. Etiology and STI/HIV Coinfections Among Patients With Urethral and Vaginal Discharge Syndromes in South Africa. Sexually Transmitted Diseases, 37(9), 566-570.

259. Miller M, Liao Y, Gomez AM, Gaydos CA, D'Mellow D. 2008. Factors associated with the prevalence and incidence of Trichomonas vaginalis infection among African American women in New York city who use drugs. Journal of Infectious Diseases, 197(4), 503-509.

260. Miller WC, Zenilman JM. 2005. Epidemiology of chlamydial infection, gonorrhea, and trichomoniasis in the United States - 2005. Infectious Disease Clinics of North America, 19(2 SPEC. ISS.), 281-296.

261. Miranda A, Silva L, V Braga J, Montes R, Talhari S. 2011. Vaginal discharge in women living with HIV attending an aids clinic in Manaus, Brazil. Sexually Transmitted Infections, 87, A301.

262. Miranda AE, Pinto VM, Gaydos CA. 2014. Trichomonas vaginalis infection among young pregnant women in Brazil. Brazilian Journal of Infectious Diseases, 18(6), 669-671.

263. Mitchell HD, Lewis DA, Marsh K, Hughes G. 2014. Distribution and risk factors of Trichomonas vaginalis infection in England: an epidemiological study using electronic health records from sexually transmitted infection clinics, 2009-2011. Epidemiology and Infection, 142(8), 1678-1687.

264. Mitteregger D, Aberle SW, Makristathis A, Walochnik J, Brozek W, Marberger M, Kramer G. 2012. High detection rate of Trichomonas vaginalis in benign hyperplastic prostatic tissue. Medical Microbiology and Immunology, 201(1), 113-116.

265. Mlisana K, Naicker N, Werner L, Roberts L, Van Loggerenberg F, Baxter C, Passmore JAS, Grobler AC, Sturm AW, Williamson C, Ronacher K, Walzl G, Abdool Karim SS. 2012. Symptomatic vaginal discharge is a poor predictor of sexually transmitted infections and genital tract inflammation in high-risk women in South Africa. Journal of Infectious Diseases, 206(1), 6-14.

266. Msuya SE, Mbizvo EM, Stray-Pedersen B, Uriyo J, Sam NE, Rusakaniko S, Hussain A. 2007. Decline in HIV prevalence among women of childbearing age in Moshi urban, Tanzania. Int J STD AIDS, 18(10), 680-7.

267. Msuya SE, Uriyo J, Hussain A, Mbizvo EM, Jeansson S, Sam NE, Stray-Pedersen B. 2009. Prevalence of sexually transmitted infections among pregnant women with known HIV status in northern Tanzania. Reproductive Health, 6(1).

268. Munoz-Ramirez A, Lopez-Monteon A, Ramos-Ligonio A, Mendez-Bolaina E, Guapillo-Vargas MRB. 2018. Prevalence of Trichomonas vaginalis and Human papillomavirus in female sex workers in Central Veracruz, Mexico. Revista Argentina De Microbiologia, 50(4), 351-358.

269. Munson E, Wenten D, Phipps P, Gremminger R, Schuknecht MK, Napierala M, Hamer D, Olson R, Schell RF, Hryciuk JE. 2013. Retrospective assessment of transcription-mediated amplification-based screening for Trichomonas vaginalis in male sexually transmitted infection clinic patients. J Clin Microbiol, 51(6), 1855-60.

270. Muzny CA, Schwebke JR. 2013. The clinical spectrum of Trichomonas vaginalis infection and challenges to management. Sexually Transmitted Infections, 89(6), 423-425.

271. Muzny CA, Sunesara IR, Martin DH, Mena LA. 2011. Sexually Transmitted Infections and Risk Behaviors Among African American Women Who Have Sex With Women: Does Sex With Men Make a Difference? Sexually Transmitted Diseases, 38(12), 1118-1125.

272. Mwatelah R, Peterson S, Bonner C, Omole T, Nuhu F, Jahan N, King'Ola N, Wambua S, Gichangi P, Cheuk E, McClarty G, Martin I, Becker M, Mishra S, McKinnon L. 2019. Prevalence and mucosal impact of STIS in young women from Mombasa, Kenya with varying exposure to sex work. Sexually Transmitted Infections, 95, A176-A177.

273. Nagot N, Ouedraogo A, Defer MC, Vallo R, Mayaud P, Van de Perre P. 2007. Association between bacterial vaginosis and Herpes simplex virus type-2 infection: implications for HIV acquisition studies. Sex Transm Infect, 83(5), 365-8.

274. Naidoo K, Naidoo M, Gengiah S, Nkupiso N, Singh C, Leask K, Kharsany ABM. 2013. Genital tract abnormalities in HIV-TB Co-infected women initiating antiretroviral therapy (ART). Sexually Transmitted Infections, 89.

275. Nateghi Rostami M, Hossein Rashidi B, Habibi A, Nazari R, Dolati M. 2017. Genital infections and reproductive complications associated with Trichomonas vaginalis, Neisseria gonorrhoeae, and Streptococcus agalactiae in women of Qom, central Iran. Int J Reprod Biomed, 15(6), 357-366.

276. Navaratine L, Clune M, Proom T, Whalen S, Apea V, Twydell E, Hill P, Scott J, Wareham N. 2022. TESTING 7376 SAMPLES FOR TRICHOMONAS VAGINALIS USING NAAT TESTING IN SYMPTOMATIC FEMALES WITH VAGINAL DISCHARGE AND ASYMPTOMATIC FEMALES OF ALL ETHNICITIES THROUGH AN ONLINE STI TESTING SERVICE. Sexually Transmitted Infections, 98, A44-A45.

277. Nelson A, Press N, Bautista CT, Arevalo J, Calderon M, Campos K, Bryant A, Shantz-Dunn J, Dahodwala N, Vera M, Vivar A, Saito M, Gilman RH. 2007. Prevalence of sexually transmitted infections and high-risk sexual Behaviors in heterosexual couples attending sexually transmitted disease clinics in Peru. Sexually Transmitted Diseases, 34(6), 344-361.

278. Nessa K, Waris SA, Alam A, Huq M, Nahar S, Chawdhury FA, Monira S, Badal MU, Sultana J, Mahmud KF, Das J, Mitra DK, Sultan Z, Hossain N, Rahman M. 2005. Sexually transmitted infections among brothel-based sex workers in bangladesh: high prevalence of asymptomatic infection. Sex Transm Dis, 32(1), 13-9.

279. Nessa K, Waris SA, Sultan Z, Monira S, Hossain M, Nahar S, Rahman H, Alam M, Baatsen P, Rahman M. 2004. Epidemiology and etiology of sexually transmitted infection among hotel-based sex workers in Dhaka, Bangladesh. Journal of Clinical Microbiology, 42(2), 618-621.

280. Ng A, Ross J. 2012. Nuclei acid amplification tests (NAAT) for Trichomonas vaginalis: Should they change who we screen for infection. Sexually Transmitted Infections, 88.

281. Ngugi EN, Fonck K, Moses S, Kaul R, Temmerman M, Kimani J, Keli F, MacDonald KS, Ronald AR, Plummer FA, Kirui P, Bwayo JJ. 2000. A randomized, placebo-controlled trial of monthly azithromycin prophylaxis to prevent sexually transmitted infections and hiv-1 in Kenyan sex workers: Study design and baseline findings. International Journal of STD and AIDS, 11(12), 804-811.

282. Nguyen M, Giang LM, Hinh ND, Klausner J. 2017. Prevalence of sexually transmitted infections and acceptability, feasibility of screening in antenatal care, Vietnam, 2016-2017. Sexually Transmitted Infections, 93, A152-A153.

283. Nguyen M, Le GM, Nguyen HTT, Nguyen HD, Klausner JD. 2019. Acceptability and feasibility of sexually transmissible infection screening among pregnant women in Hanoi, Vietnam. Sexual Health, 16(2), 133-138.

284. Nguyen TV, Khuu NV, Truong PH, Nguyen AP, Truong LX, Detels R. 2009. Correlation between HIV and sexual behavior, drug use, trichomoniasis and candidiasis among female sex workers in a Mekong Delta province of Vietnam. AIDS Behav, 13(5), 873-80.

285. Nguyen TV, Van Khuu N, Thi Le TT, Nguyen AP, Cao V, Tham DC, Detels R. 2008. Sexually transmitted infections and risk factors for gonorrhea and chlamydia in female sex workers in Soc Trang, Vietnam. Sex Transm Dis, 35(11), 935-40.

286. Nijhawan AE, Chapin KC, Salloway R, Andrea S, Champion J, Roberts M, Clarke JG. 2012. Prevalence and predictors of trichomonas infection in newly incarcerated women. Sex Transm Dis, 39(12), 973-8.

287. Nijhuis RHT, Duinsbergen RG, Pol A, Godschalk PCR. 2021. Prevalence of Chlamydia trachomatis, Neisseria gonorrhoeae, Mycoplasma genitalium and Trichomonas vaginalis including relevant resistance-associated mutations in a single center in the Netherlands. Eur J Clin Microbiol Infect Dis, 40(3), 591-595.

288. Nikpay S, Otaghi M, Azami M, Karimi M, Abdi J. 2020. Trichomonas vaginalis infection among women attending laboratory centers in Ilam, Iran. Infectious Disorders - Drug Targets, 20(1), 98-101.

289. Nolan MS, Cruz AT, Erickson T. 2020. Retrospective Chart Analysis of Child and Adolescent Trichomonas vaginalis Infection in Houston, Texas. Journal of the Pediatric Infectious Diseases Society, 9(1), 75-81.

290. Nomelini RS, Carrijo APB, Adad SJ, Nunes AA, Murta EFC. 2010. Relationship between infectious agents for vulvovaginitis and skin color. Sao Paulo Medical Journal, 128(6), 348-353.

291. Nourian A, Shabani N, Fazaeli A, Mousavinasab SN. 2013. Prevalence of Trichomonas vaginalis in pregnant women in Zanjan, Northwest of Iran. Jundishapur Journal of Microbiology, 6(8).

292. Novotna L, Wilson TE, Minkoff HL, McNutt LA, DeHovitz JA, Ehrlich I, Des Jarlais DC. 1999. Predictors and risk-taking consequences of drug use among HIV-infected women. Journal of Acquired Immune Deficiency Syndromes, 20(5), 502-507.

293. Nwadioha S, Egesie JO, Emejuo H, Iheanacho E. 2010. Prevalence of pathogens of abnormal vaginal discharges in a Nigerian tertiary hospital. Asian Pacific Journal of Tropical Medicine, 3(6), 483-485.

294. Nyein S, Clarke E, Ahmed S, Foley E, Ossey C, Patel R. 2015. Low rates of Trichomonas vaginalis in a UK sexually transmitted infection clinic population - New tests may only generate a modest improvement over light microscopy in some settings. International Journal of STD and AIDS, 26(11), 87.

295. Nyemba DC, Ngwepe PP, Peters RP, Klausner JD, Myer L, Davey DJ, Medina-Marino A, Johnson LF. 2020. Prevalence and incidence of STIS during pregnancy in South Africa. Topics in Antiviral Medicine, 28(1), 393.

296. Oakeshott P, Ahmed J, Hay PE, Reid F, Kerry SR, Aghaizu A, Tong CYW. 2011. Trichomonas vaginalis among multiethnic female UK students. Sexually Transmitted Infections, 87(5), 369.

297. Occhionero M, Paniccia L, Pedersen D, Vaulet LG, Entrocassi C, Fermepin MR. 2018. Vaginal dysfunction prevalence in women of the city of Bahia Blanca (Argentina). Acta Bioquimica Clinica Latinoamericana, 52(4), 429-439.

298. Ok Atılgan A, Tepeoğlu M, Haberal AN, Durukan E, Kuşcu E, Haberal M. 2015. Papanicolaou smear findings in solid-organ transplant recipients compared with normal subjects according to the Bethesda 2001 system. Exp Clin Transplant, 13 Suppl 1, 219-22.

299. Oliphant J, Azariah S. 2016. Pelvic inflammatory disease associated with Chlamydia trachomatis but not Mycoplasma genitalium in New Zealand. Sexual Health, 13(1), 43-48.

300. Ong VA, Rivera WL. 2010. Prevalence of trichomonas vaginalis in vaginal swabs from sex workers in Angeles City, Pampanga, Philippines as detected by PCR. Tropical Medicine and Health, 38(1), 29-34.

301. Ononge S, Wandabwa J, Kiondo P, Busingye R. 2005. Clinical presentation and management of alleged sexually assaulted females at Mulago hospital, Kampala, Uganda. African Health Sciences, 5(1), 50-54.

302. Op de Coul ELM, Peek D, van Weert YWM, Morré SA, Rours I, Hukkelhoven C, de Jonge A, van Benthem B, Pereboom M. 2021. Chlamydia trachomatis, Neisseria gonorrhoea, and Trichomonas vaginalis infections among pregnant women and male partners in Dutch midwifery practices: prevalence, risk factors, and perinatal outcomes. Reprod Health, 18(1), 132.

303. Ortayli N, Sahip Y, Amca B, Say L, Sahip N, Aydin D. 2001. Curable sexually transmitted infections among the clientele of a family planning clinic in Istanbul, Turkey. Sexually Transmitted Diseases, 28(1), 58-61.

304. Otuonye NM, Odunukwe NN, Idigbe EO, Imosemi OD, Smith SI, Chigbo RC, Bamidele M, Oparaugo CT, Mafe AG, Musa AZ. 2004. Aetiological agents of vaginitis in Nigerian women. British Journal of Biomedical Science, 61(4), 175-178.

305. Ovalle A, Martinez MA, de la Fuente F, Falcon N, Feliu F, Fuentealba F, Gianini R. 2012. Prevalence of sexually transmitted infections in pregnant women attending a public hospital in Chile. Revista Chilena De Infectologia, 29(5), 517-520.

306. Özdemir E, Keleştemur N, Kaplan M. 2011. Trichomonas vaginalis as a rare cause of male factor infertility at a hospital in East Anatolia. Andrologia, 43(4), 283-285.

307. Park JJ, Seo YB, Jeong S, Lee J. 2017. Prevalence of and Risk Factors for Sexually Transmitted Infections among Korean Adolescents under Probation. J Korean Med Sci, 32(11), 1771-1778.

308. Passey M, Mgone CS, Lupiwa S, Suve N, Tiwara S, Lupiwa T, Clegg A, Alpers MP. 1998. Community based study of sexually transmitted diseases in rural women in the highlands of Papua New Guinea: Prevalence and risk factors. Sexually Transmitted Infections, 74(2), 120-127.

309. Paterson BA, Tabrizi SN, Garland SM, Fairley CK, Bowden FJ. 1998. The tampon test for trichomoniasis: A comparison between conventional methods and a polymerase chain reaction for Trichomonas vaginalis in women. Sexually Transmitted Infections, 74(2), 136-139.

310. Patten JH, Susanti I. 2001. Reproductive health and STDs among clients of a women's health mobile clinic in rural Bali, Indonesia. Int J STD AIDS, 12(1), 47-9.

311. Pattullo L, Griffeth S, Ding LL, Mortensen J, Reed J, Kahn J, Huppert J. 2009. Stepwise Diagnosis of Trichomonas vaginalis Infection in Adolescent Women. Journal of Clinical Microbiology, 47(1), 59-63.

312. Paul H, Peter D, Pulimood SA, Abraham OC, Mathai E, Prasad JH, Kannangai R. 2012. Role of polymerase chain reaction in the diagnosis of Trichomonas vaginalis infection in human immunodeficiency virus-infected individuals from India (South). Indian J Dermatol Venereol Leprol, 78(3), 323-7.

313. Paz-Bailey G, Morales-Miranda S, Jacobson JO, Gupta SK, Sabin K, Mendoza S, Paredes M, Alvarez B, Monterroso E. 2009. High Rates of STD and Sexual Risk Behaviors Among Garifunas in Honduras. Jaids-Journal of Acquired Immune Deficiency Syndromes, 51, S26-S34.

314. Paz-Bailey G, Rahman M, Chen C, Ballard R, Moffat HJ, Kenyon T, Kilmarx PH, Totten PA, Astete S, Boily MC, Ryan C. 2005. Changes in the etiology of sexually transmitted diseases in Botswana between 1993 and 2002: Implications for the clinical management of genital ulcer disease. Clinical Infectious Diseases, 41(9), 1304-1312.

315. Pepin J, Labbe AC, Khonde N, Deslandes S, Alary M, Dzokoto A, Asamoah-Adu C, Meda H, Frost E. 2005. Mycoplasma genitalium: an organism commonly associated with cervicitis among west African sex workers. Sexually Transmitted Infections, 81(1), 67-72.

316. Perazzi BE, Menghi CI, Coppolillo EF, Gatta C, Eliseth MC, De Torres RA, Vay CA, Famiglietti AMR. 2010. Prevalence and comparison of diagnostic methods for Trichomonas vaginalis infection in pregnant women in Argentina. Korean Journal of Parasitology, 48(1), 61-65.

317. Pereyre S, Laurier Nadalié C, Bébéar C. 2017. Mycoplasma genitalium and Trichomonas vaginalis in France: a point prevalence study in people screened for sexually transmitted diseases. Clin Microbiol Infect, 23(2), 122.e1-122.e7.

318. Perla ME, Ghee AE, Sánchez S, McClelland RS, Fitzpatrick AL, Suárez-Ognio L, Lama JR, Sánchez J. 2012. Genital tract infections, bacterial vaginosis, HIV, and reproductive health issues among Lima-based clandestine female sex workers. Infect Dis Obstet Gynecol, 2012, 739624.

319. Perry MD, Jones S, Bertram A, de Salazar A, Barrientos-Duran A, Schiettekatte G, Lewinski M, Arcenas R, Hansra A, Njoya M, Garcia F. 2023. The prevalence of Mycoplasma genitalium (MG) and Trichomonas vaginalis (TV) at testing centers in Belgium, Germany, Spain, and the UK using the cobas TV/MG molecular assay. European Journal of Clinical Microbiology & Infectious Diseases, 42(1), 43-52.

320. Pillay A, Radebe F, Fehler G, Htun Y, Ballard RC. 2007. Comparison of a TaqMan-based real-time polymerase chain reaction with conventional tests for the detection of Trichomonas vaginalis. Sex Transm Infect, 83(2), 126-9.

321. Pintye J, Drake AL, Unger JA, Matemo D, Kinuthia J, McClelland RS, John-Stewart G. 2017. Male partner circumcision associated with lower Trichomonas vaginalis incidence among pregnant and postpartum Kenyan women: a prospective cohort study. Sexually Transmitted Infections, 93(2), 137-143.

322. Piperaki ET, Theodora M, Mendris M, Barbitsa L, Pitiriga V, Antsaklis A, Tsakris A. 2010. Prevalence of Trichomonas vaginalis infection in women attending a major gynaecological hospital in Greece: a cross-sectional study. Journal of Clinical Pathology, 63(3), 249-253.

323. Plaas K, Eelmets L, Ratnik K, Jänes J, Rüütel K, Naaber P. 2015. Sexually transmitted infections among men who have sex with men: Prevalence, locations and challenges for molecular assays. International Journal of STD and AIDS, 26(11), 102.

324. Plitt SS, Garfein RS, Gaydos CA, Strathdee SA, Sherman SG, Taha TE. 2005. Prevalence and correlates of Chlamydia trachomatis, Neisseria gonorrhoeae, Trichomonas vaginalis infections, and bacterial vaginosis among a cohort of young injection drug users in Baltimore, Maryland. Sexually Transmitted Diseases, 32(7), 446-453.

325. Pollett S, Calderon M, Heitzinger K, Solari V, Montano SM, Zunt J. 2013. Prevalence and predictors of cervicitis in female sex workers in Peru: An observational study. BMC Infectious Diseases, 13(1).

326. Price MA, Miller WC, Kaydos-Daniels SC, Hoffman IF, Chilongozi D, Martinson FE, Namakhwa D, Malanda J, Cohen M. 2004. Trichomoniasis in men and HIV infection: Data from 2 outpatient clinics at Lilongwe Central Hospital, Malawi. Journal of Infectious Diseases, 190(8), 1448-1455.

327. Rahman M, Alam A, Nessa K, Hossain A, Nahar S, Datta D, Alam Khan S, Amin Mian R, Albert MJ. 2000. Etiology of sexually transmitted infections among street-based female sex workers in Dhaka, Bangladesh. J Clin Microbiol, 38(3), 1244-6.

328. Rahman S, Garland S, Currie M, Tabrizi SN, Rahman M, Nessa K, Bowden FJ. 2008. Prevalence of Mycoplasma genitalium in health clinic attendees complaining of vaginal discharge in Bangladesh. International Journal of Std & Aids, 19(11), 772-774.

329. Rajabpour M, Emamie AD, Pourmand MR, Goodarzi NN, Asbagh FA, Whiley DM. 2020. Chlamydia trachomatis, Neisseria gonorrhoeae, andTrichomonas vaginalisamong women with genitourinary infection and pregnancy-related complications in Tehran: A cross-sectional study. International Journal of Std & Aids, 31(8), 773-780.

330. Ramia S, Kobeissi L, El Kak F, Shamra S, Kreidieh K, Zurayk H. 2012. Reproductive tract infections (RTIs) among married non-pregnant women living in a low-income suburb of Beirut, Lebanon. J Infect Dev Ctries, 6(9), 680-3.

331. Rao K, Madhivanan P, Klausner J, Trammell S, Kotian S, Ravi K, Krupp K, Srinivas V. 2011. Evaluation of a simple point-of-care rapid test for detecting trichomonas vaginalis among women in Mysore, India. Sexually Transmitted Infections, 87, A299.

332. Rao VG, Anvikar A, Savargaonkar D, Bhat J. 2009. Sexually transmitted infections in tribal populations of central India. European Journal of Clinical Microbiology and Infectious Diseases, 28(11), 1391-1393.

333. Rassjo EB, Kambugu F, Tumwesigye MN, Tenywa T, Darj E. 2006. Prevalence of sexually transmitted infections among adolescents in Kampala, Uganda, and theoretical models for improving syndromic management. Journal of Adolescent Health, 38(3), 213-221.

334. Rasti S, Assadi MA, Behrashi M, Moosavi G. 2012. Effects of vaginal candidiasis and trichomoniasis on newborn. International Journal of Gynecology and Obstetrics, 119, S721.

335. Rathod SD, Krupp K, Klausner JD, Arun A, Reingold AL, Madhivanan P. 2011. Bacterial Vaginosis and Risk for Trichomonas vaginalis Infection: A Longitudinal Analysis. Sexually Transmitted Diseases, 38(9), 882-886.

336. Reza-Paul S, Beattie T, Syed HU, Venukumar KT, Venugopal MS, Fathima MP, Raghavendra HR, Akram P, Manjula R, Lakshmi M, Isac S, Ramesh BM, Washington R, Mahagaonkar SB, Glynn JR, Blanchard JF, Moses S. 2008. Declines in risk behaviour and sexually transmitted infection prevalence following a community-led HIV preventive intervention among female sex workers in Mysore, India. Aids, 22 Suppl 5, S91-100.

337. Riley ED, Cohen J, Dilworth SE, Grimes B, Marquez C, Chin-Hong P, Philip SS. 2016. Trichomonas vaginalis infection among homeless and unstably housed adult women living in a resource-rich urban environment. Sex Transm Infect, 92(4), 305-8.

338. Robinson AJ, Watkeys JEM, Ridgway GL. 1998. Sexually transmitted organisms in sexually abused children. Archives of Disease in Childhood, 79(4), 356-358.

339. Rocha DAP, Barbosa RAA, Marino JM, dos Santos CMB. 2014. "Hidden" sexually transmitted infections among women in primary care health services, Amazonas, Brazil. International Journal of Std & Aids, 25(12), 878-886.

340. Rogers S, Turner C, Miller W, Roman A, Hobbs M, Tan S. 2011. Increased risk for trichomonas vaginalis in an urban population of young adults. Sexually Transmitted Infections, 87, A33.

341. Ross DA, Changalucha J, Obasi AIN, Todd J, Plummer ML, Cleophas-Mazige B, Anemona A, Everett D, Weiss HA, Mabey DC, Grosskurth H, Hayes RJ, Balira R, Wight D, Gavyole A, Makokha MJ, Mosha F, Terris-Prestholt F, Parry JV. 2007. Biological and behavioural impact of an adolescent sexual health intervention in Tanzania: a community-randomized trial. Aids, 21(14), 1943-1955.

342. Rukasha I, Ehlers MM, Kock MM. 2013. Trichomonas Vaginalis detection and characterization from women attending an anti-retroviral clinic in Pretoria, South Africa. Sexually Transmitted Infections, 89.

343. Ryder N, Woods H, McKay K, Giddings N, Lenton JA, Little C, Jeoffreys N, McNulty AM. 2012. Trichomonas vaginalis Prevalence Increases With Remoteness in Rural and Remote New South Wales, Australia. Sexually Transmitted Diseases, 39(12), 938-941.

344. Saleh-Onoya D, Reddy PS, Ruiter RAC, Sifunda S, Wingood G, Van Den Borne B. 2009. Condom use promotion among isiXhosa speaking women living with HIV in the Western Cape Province, South Africa: A pilot study. AIDS Care - Psychological and Socio-Medical Aspects of AIDS/HIV, 21(7), 817-825.

345. Samarawickrema NA, Tabrizi SN, Young E, Gunawardena P, Garland SM. 2015. Prevalence of Trichomonas vaginalis, Chlamydia trachomatis, Neisseria gonorrhoeae and human papillomavirus in a sexual health clinic setting in urban Sri Lanka. Int J STD AIDS, 26(10), 733-9.

346. Sangaré I, Guiguemdé KT, Zida A, Sirima C, Sawadogo PM, Cissé M, Assogba SB, Guiguemdé TR, Bamba S. 2021. Prevalence of intestinal parasitic infections among pregnant women in Bobo-Dioulasso (Burkina Faso). Ann Parasitol, 67(3), 489-497.

347. Schnatz PF, Markelova NV, Holmes D, Mandavilli SR, O'Sullivan DM. 2008. The prevalence of cervical HPV and cytological abnormalities in association with reproductive factors of rural Nigerian women. Journal of Women's Health, 17(2), 279-285.

348. Schwebke JR, Gaydos CA, Davis T, Marrazzo J, Furgerson D, Taylor SN, Smith B, Bachmann LH, Ackerman R, Spurrell T, Ferris D, Burnham CA, Reno H, Lebed J, Eisenberg D, Kerndt P, Philip S, Jordan J, Quigley N. 2018. Clinical Evaluation of the Cepheid Xpert TV Assay for Detection of Trichomonas vaginalis with Prospectively Collected Specimens from Men and Women. J Clin Microbiol, 56(2).

349. Seay J, Mandigo M, Hew K, Kobetz E. 2017. Vaginal Infections in Haitian Immigrant Women Living in Miami, Florida. J Health Care Poor Underserved, 28(3), 1141-1150.

350. Sena A, Mallette K. 2018. Reflex nucleic acid amplification testing for trichomonas vaginalis among women with negative wet mount microscopy in an STI clinic. Sexually Transmitted Diseases, 45, S67.

351. Seo MY, Im SJ, Gu NY, Kim JH, Chung YH, Ahn MH, Ryu JS. 2014. Inflammatory response of prostate epithelial cells to stimulation by Trichomonas vaginalis. Prostate, 74(4), 441-449.

352. Seth P, Wingood GM, Diclemente RJ. 2008. Exposure to alcohol problems and its association with sexual behaviour and biologically confirmed Trichomonas vaginalis among women living with HIV. Sex Transm Infect, 84(5), 390-2.

353. Shahmanesh M, Cowan F, Wayal S, Copas A, Patel V, Mabey D. 2009. The burden and determinants of HIV and sexually transmitted infections in a population-based sample of female sex workers in Goa, India. Sexually Transmitted Infections, 85(1), 50-59.

354. Shaw J, Currie S, Hatley A, Shafiq V, Leighton J, Lee V. 2015. Watching the TV: Trichomonas vaginalis NAAT testing in an inner city sexual health clinic. Sexually Transmitted Infections, 91, A66.

355. Shaw MK, Porterfield HS, Favaloro S, Dehon PM, Van Der Pol B, Quayle AJ, McGowin CL. 2019. Prevalence and cervical organism burden among Louisiana women with Trichomonas vaginalis infections. PLoS ONE, 14(6).

356. Sieck CJ, Dembe AE. 2011. Results of a pilot study of pre-release STD testing and inmates' risk behaviors in an Ohio prison. J Urban Health, 88(4), 690-9.

357. Silitonga N, Davies SC, Kaldor J, Wignall S, Okoseray M. 2011. Prevalence over time and risk factors for sexually transmissible infections among newly-arrived female sex workers in Timika, Indonesia. Sex Health, 8(1), 61-4.

358. Silva LCF, Miranda AE, Batalha RS, Monte RL, Talhari S. 2013. Trichomonas vaginalis and associated factors among women living with HIV/AIDS in Amazonas, Brazil. Brazilian Journal of Infectious Diseases, 17(6), 701-703.

359. Singh RH, Zenilman JM, Brown KM, Madden T, Gaydos C, Ghanem KG. 2013. The role of physical examination in diagnosing common causes of vaginitis: a prospective study. Sex Transm Infect, 89(3), 185-90.

360. Smith KS, Tabrizi SN, Fethers KA, Knox JB, Pearce C, Garland SM. 2005. Comparison of conventional testing to polymerase chain reaction in detection of Trichomonas vaginalis in indigenous women living in remote areas. Int J STD AIDS, 16(12), 811-5.

361. Snead M, Wiener J, Phillips C, Hylton-Kong T, Medley-Singh N, Legardy-Williams J, Costenbader B, Papp J, Warner L, Kourtis A. 2016. Prevalence and predictors of stis among women initiating contraceptive implants in kingston, Jamaica. Sexually Transmitted Diseases, 43(10), S191.

362. Soares VD, de Mesquita A, Cavalcante FGT, Silva ZP, Hora V, Diedrich T, Silva PD, de Melo PG, Dacal ARC, de Carvalho EMF, Feldmeier H. 2003. Sexually transmitted infections in a female population in rural north-east Brazil: prevalence, morbidity and risk factors. Tropical Medicine & International Health, 8(7), 595-603.

363. Sobngwi-Tambekou J, Taljaard D, Nieuwoudt M, Lissouba P, Puren A, Auvert B. 2009. Male circumcision and Neisseria gonorrhoeae, Chlamydia trachomatis and Trichomonas vaginalis: observations after a randomised controlled trial for HIV prevention. Sexually Transmitted Infections, 85(2), 116-120.

364. Spinillo A, Bernuzzi AM, Cevini C, Gulminetti R, Luzi S, De Santolo A. 1997. The relationship of bacterial vaginosis, Candida and Trichomonas infection to symptomatic vaginitis in postmenopausal women attending a vaginitis clinic. Maturitas, 27(3), 253-60.

365. Squire DS, Lymbery AJ, Walters J, Ahmed H, Asmah RH, Thompson RCA. 2019. Trichomonas vaginalis infection in southern Ghana: clinical signs associated with the infection. Trans R Soc Trop Med Hyg, 113(7), 359-369.

366. Srugo I, Steinberg J, Madeb R, Gershtein R, Elias I, Tal J, Nativ O. 2003. Agents of non-gonococcal urethritis in males attending an Israeli clinic for sexually transmitted diseases. Israel Medical Association Journal, 5(1), 24-27.

367. Strathdee SA, Lozada R, Martinez G, Vera A, Rusch M, Nguyen L, Pollini RA, Uribe-Salas F, Beletsky L, Patterson TL. 2011. Social and structural factors associated with HIV infection among female sex workers who inject drugs in the Mexico-US border region. PLoS One, 6(4), e19048.

368. Su RY, Ho LJ, Yang HY, Chung CH, Yang SS, Cheng CY, Chien WC, Lin HC. 2020. Association between Trichomonas vaginalis infection and cervical lesions: a population-based, nested case-control study in Taiwan. Parasitology Research, 119(8), 2649-2657.

369. Sullivan EA, Abel M, Tabrizi S, Garland SM, Grice A, Poumerol G, Taleo H, Chen S, Kaun K, O'Leary M, Kaldor J. 2003. Prevalence of sexually transmitted infections among antenatal women in Vanuatu, 1999-2000. Sexually Transmitted Diseases, 30(4), 362-366.

370. Sullivan EA, Koro S, Tabrizi S, Kaldor J, Poumerol G, Chen S, O'Leary M, Garland SM. 2004. Prevalence of sexually transmitted diseases and human immunodeficiency virus among women attending prenatal services in Apia, Samoa. International Journal of Std & Aids, 15(2), 116-119.

371. Sutton MY, Sternberg M, Kouman E, McQuuillan G, Berman S, Markowitz L. 2006. The prevalence of trichomonas vaginalis in the united states, 2001-2002. Obstetrics and Gynecology, 107(4), 8S-8S.

372. Sviben M, Missoni EM, Meštrović T, Vojnović G, Galinović GM. 2015. Epidemiology and laboratory characteristics of Trichomonas vaginalis infection in Croatian men with and without urethritis syndrome: a case-control study. Sex Transm Infect, 91(5), 360-4.

373. Takei H, Ruiz B, Hicks J. 2006. Cervicovaginal flora. Comparison of conventional pap smears and a liquid-based thin-layer preparation. Am J Clin Pathol, 125(6), 855-9.

374. Tann CJ, Mpairwe H, Morison L, Nassimu K, Hughes P, Omara M, Mabey D, Muwanga M, Grosskurth H, Elliott AM. 2006. Lack of effectiveness of syndromic management in targeting vaginal infections in pregnancy in Entebbe, Uganda. Sex Transm Infect, 82(4), 285-9.

375. Tchankoni MK, Bitty-Anderson AM, Sadio AJ, Gbeasor-Komlanvi FA, Ferré VM, Zida-Compaore WIC, Dorkenoo AM, Saka B, Dagnra AC, Charpentier C, Ekouevi DK. 2021. Prevalence and factors associated with trichomonas vaginalis infection among female sex workers in Togo, 2017. BMC Infect Dis, 21(1), 775.

376. Tibaldi C, Cappello N, Latino MA, Masuelli G, Marini S, Benedetto C. 2009. Vaginal and endocervical microorganisms in symptomatic and asymptomatic non-pregnant females: risk factors and rates of occurrence. Clinical Microbiology and Infection, 15(7), 670-679.

377. Timm N, Bouvay K, Scheid B, Defoor WR. 2011. Evaluation and Management of Sexually Transmitted Infections in Adolescent Males Presenting to a Pediatric Emergency Department Is the Chief Complaint Diagnostic? Pediatric Emergency Care, 27(11), 1042-1044.

378. Tine RC, Dia L, Sylla K, Sow D, Lelo S, Ndour CT. 2019. Trichomonas vaginalis and Mycoplasma infections among women with vaginal discharge at Fann teaching hospital in Senegal. Trop Parasitol, 9(1), 45-53.

379. Tjagur S, Mändar R, Punab M. 2018. Prevalence of Mycoplasma genitalium and other sexually transmitted infections causing urethritis among high-risk heterosexual male patients in Estonia. Infectious Diseases, 50(2), 133-139.

380. Toboso Silgo L, Cruz-Melguizo S, de la Cruz Conty ML, Encinas Pardilla MB, Muñoz Algarra M, Nieto Jiménez Y, Arranz Friediger A, Martínez-Pérez Ó. 2021. Screening for Vaginal and Endocervical Infections in the First Trimester of Pregnancy? A Study That Ignites an Old Debate. Pathogens, 10(12).

381. Tolosa J, Rodriguez A, Angel Muller E, Ruiz Parra A, Gaitan Duarte H. 2013. Accuracy of syndromic diagnosis (SD) for vaginal dyscharge and cervicitis in women of reproductive Age in Bogota, Colombia. Sexually Transmitted Infections, 89.

382. Turner AN, Feldblum PJ, Hoke TH. 2010. Baseline infection with a sexually transmitted disease is highly predictive of reinfection during follow-up in Malagasy sex workers. Sex Transm Dis, 37(9), 559-62.

383. Uma S, Balakrishnan P, Murugavel KG, Srikrishnan AK, Kumarasamy N, Anand S, Cecelia JA, Celentano D, Mayer KH, Thyagarajan SP, Solomon S. 2006. Bacterial vaginosis in women of low socioeconomic status living in slum areas in Chennai, India. Sex Health, 3(4), 297-8.

384. Upcroft JA, Dunn LA, Wal T, Tabrizi S, Delgadillo-Correa MG, Johnson PJ, Garland S, Siba P, Upcroft P. 2009. Metronidazole resistance in Trichomonas vaginalis from highland women in Papua New Guinea. Sexual Health, 6(4), 334-338.

385. Upreti P. 2019. Prevalence of stis among Nepalese women population. Sexually Transmitted Infections, 95, A164.

386. Upton A, Bissessor L, Lowe P, Wang X, McAuliffe G. 2018. Diagnosis of Chlamydia trachomatis, Neisseria gonorrhoeae, Trichomonas vaginalis and Mycoplasma genitalium: an observational study of testing patterns, prevalence and co-infection rates in northern New Zealand. Sex Health, 15(3), 232-237.

387. Vaca M, Guadalupe I, Erazo S, Tinizaray K, Chico ME, Cooper PJ, Hay P. 2010. High prevalence of bacterial vaginosis in adolescent girls in a tropical area of Ecuador. Bjog, 117(2), 225-8.

388. Vallely A, Ryan CE, Allen J, Sauk JC, Simbiken CS, Wapling J, Kaima P, Kombati Z, Law G, Fehler G, Murray JM, Siba P, Kaldor JM. 2014. High prevalence and incidence of HIV, sexually transmissible infections and penile foreskin cutting among sexual health clinic attendees in Papua New Guinea. Sex Health, 11(1), 58-66.

389. van der Veer C, Himschoot M, Bruisten SM. 2016. Multilocus sequence typing of Trichomonas vaginalis clinical samples from Amsterdam, the Netherlands. BMJ Open, 6(10), e013997.

390. Vandepitte J, Muller E, Bukenya J, Nakubulwa S, Kyakuwa N, Buve A, Weiss H, Hayes R, Grosskurth H. 2012. Prevalence and Correlates of Mycoplasma genitalium Infection Among Female Sex Workers in Kampala, Uganda. Journal of Infectious Diseases, 205(2), 289-296.

391. Verteramo R, Pierangeli A, Mancini E, Calzolari E, Bucci M, Osborn J, Nicosia R, Chiarini F, Antonelli G, Degener AM. 2009. Human Papillomaviruses and genital co-infections in gynaecological outpatients. BMC Infect Dis, 9, 16.

392. Vijaya Mn D, Umashankar K, Sudha, Nagure AG, Kavitha G. 2013. Prevalence of the trichomonas vaginalis infection in a tertiary care hospital in rural bangalore, southern India. J Clin Diagn Res, 7(7), 1401-3.

393. Vishwanath S, Talwar V, Prasad R, Coyaji K, Elias CJ, De Zoysa I. 2000. Syndromic management of vaginal discharge among women in a reproductive health clinic in India. Sexually Transmitted Infections, 76(4), 303-306.

394. von Glehn MP, Sá LCEF, da Silva HDF, Machado ER. 2017. Prevalence of trichomonas vaginalis in women of reproductive age at a family health clinic. Journal of Infection in Developing Countries, 11(3), 269-276.

395. Vuylsteke B, Semde G, Sika L, Crucitti T, Ettiegne Traore V, Buve A, Laga M. 2012. High prevalence of HIV and sexually transmitted infections among male sex workers in Abidjan, Cote d'Ivoire: need for services tailored to their needs. Sex Transm Infect, 88(4), 288-93.

396. Vuylsteke B, Semde G, Sika L, Crucitti T, Traore VE, Buve A, Laga M. 2012. HIV and STI Prevalence among Female Sex Workers in Cote d'Ivoire: Why Targeted Prevention Programs Should Be Continued and Strengthened. Plos One, 7(3).

397. Vuylsteke BL, Ettiegne-Traore V, Anoma CK, Bandama C, Ghys PD, Maurice CE, Van Dyck E, Wiktor SZ, Laga M. 2003. Assessment of the validity of and adherence to sexually transmitted infection algorithms at a female sex worker clinic in Abidjan, Cote D'Ivoire. Sexually Transmitted Diseases, 30(4), 284-291.

398. Vuylsteke BL, Ettiègne-Traore V, Anoma CK, Bandama C, Ghys PD, Maurice CE, Van Dyck E, Wiktor SZ, Laga M. 2003. Assessment of the validity of and adherence to sexually transmitted infection algorithms at a female sex worker clinic in Abidjan, Côte d'Ivoire. Sex Transm Dis, 30(4), 284-91.

399. Wang H, Reilly KH, Smith MK, Brown K, Jin X, Xu J, Ding G, Zang C, Wang J, Wang N. 2013. Herpes simplex virus type 2 incidence and associated risk factors among female sex workers in a high HIV-prevalence area of China. International Journal of STD and AIDS, 24(6), 441-446.

400. Wang H, Wang N, Bi A, Wang G, Ding G, Jia M, Lu L, Smith K. 2009. Application of cumulative odds logistic model on risk factors analysis for sexually transmitted infections among female sex workers in Kaiyuan city, Yunnan province, China. Sex Transm Infect, 85(4), 290-5.

401. Wang HB, Smith K, Brown KS, Wang GX, Chang DF, Xu JJ, Ding GW, Jin X, Reilly KH, Wang N. 2011. Prevalence, incidence, and persistence of syphilis infection in female sex workers in a Chinese province. Epidemiology and Infection, 139(9), 1401-1409.

402. Wang HB, Wang N, Chen RY, Sharp GB, Ma YL, Wang GX, Ding GW, Wu ZL. 2008. Prevalence and predictors of herpes simplex virus type 2 infection among female sex workers in Yunnan Province, China. International Journal of Std & Aids, 19(9), 635-639.

403. Wang PD, Lin RS. 1995. Epidemiologic differences between candidial and trichomonal infections as detected in cytologic smears in Taiwan. Public Health, 109(6), 443-50.

404. Wangnapi RA, Soso S, Unger HW, Sawera C, Ome M, Umbers AJ, Ndrewei N, Siba P, Suen C, Vallely A, Wapling J, Ryan C, Mueller I, Rogerson SJ. 2015. Prevalence and risk factors for Chlamydia trachomatis, Neisseria gonorrhoeae and Trichomonas vaginalis infection in pregnant women in Papua New Guinea. Sexually Transmitted Infections, 91(3), 194-+.

405. Warr AJ, Pintye J, Kinuthia J, Drake AL, Unger JA, McClelland RS, Matemo D, Osborn L, John-Stewart G. 2019. Sexually transmitted infections during pregnancy and subsequent risk of stillbirth and infant mortality in Kenya: a prospective study. Sexually Transmitted Infections, 95(1), 60-66.

406. Watson-Jones D, Mugeye K, Mayaud P, Ndeki L, Todd J, Mosha F, West B, Cleophas-Frisch B, Grosskurth H, Laga M, Hayes R, Mabey D, Buve A. 2000. High prevalence of trichomoniasis in rural men in Mwanza, Tanzania: Results from a population based study. Sexually Transmitted Infections, 76(5), 355-362.

407. Watts DH, Fazarri M, Minkoff H, Hillier SL, Sha B, Glesby M, Levine AM, Burk R, Palefsky JM, Moxley M, Ahdieh-Grant L, Strickler HD. 2005. Effects of bacterial vaginosis and other genital infections on the natural history of human papillomavirus infection in HIV-1-infected and high-risk HIV-1-uninfected women. Journal of Infectious Diseases, 191(7), 1129-1139.

408. Watts DH, Springer G, Minkoff H, Hillier SL, Jacobson L, Moxley M, Justman J, Cejtin H, O'Connell C, Greenblatt RM. 2006. The occurrence of vaginal infections among HIV-infected and high-risk HIV-uninfected women - Longitudinal findings of the women"s interagency HIV study. Jaids-Journal of Acquired Immune Deficiency Syndromes, 43(2), 161-168.

409. Webb B, Graham M, Korman TM. 2022. Trichomoniasis among men presenting to a sexual health clinic in Melbourne, Australia. Sex Health, 19(5), 484-485.

410. West BS, Becerra Ramirez M, Bristow CC, Abramovitz DA, Vera A, Staines H, Gudelia Rangel M, Patterson TL, Strathdee SA. 2020. Correlates of trichomoniasis among female sex workers who inject drugs in two Mexico-US border cities. Int J STD AIDS, 31(9), 866-875.

411. Wilkinson D, Ndovela N, Harrison A, Lurie M, Connolly C, Sturm AW. 1997. Family planning services in developing countries: an opportunity to treat asymptomatic and unrecognised genital tract infections? Genitourinary Medicine, 73(6), 558-560.

412. Wilkinson D, Ndovela N, Harrison A, Lurie M, Connolly C, Sturm AW. 1997. Family planning services in developing countries: an opportunity to treat asymptomatic and unrecognised genital tract infections? Genitourin Med, 73(6), 558-60.

413. Willers DM, Peipert JF, Allsworth JE, Stein MD, Rose JS, Clarke JG. 2008. Prevalence and predictors of sexually transmitted infection among newly incarcerated females. Sexually Transmitted Diseases, 35(1), 68-72.

414. Wynn A, Ramogola-Masire D, Gaolebale P, Moshashane N, Offorjebe OA, Arena K, Klausner JD, Morroni C. 2016. Acceptability and Feasibility of Sexually Transmitted Infection Testing and Treatment among Pregnant Women in Gaborone, Botswana, 2015. Biomed Research International, 2016.

415. Xu JJ, Wang N, Lu L, Pu Y, Zhang GL, Wong M, Wu ZL, Zheng XW. 2008. HIV and STIs in clients and female sex workers in mining regions of Gejiu city, China. Sexually Transmitted Diseases, 35(6), 558-565.

416. Yaro MB, Qadeer MA. 2017. Comparative study on trichomonas vaginalis among hostel-based students of college of education, hong, adamawa state, nigeria. Nigerian Journal of Parasitology, 38(1), 117-120.

417. Yasin J, Ayalew G, Dagnaw M, Shiferaw G, Mekonnen F. 2021. Vulvovaginitis Prevalence Among Women in Gondar, Northwest Ethiopia: Special Emphasis on Aerobic Vaginitis Causing Bacterial Profile, Antimicrobial Susceptibility Pattern, and Associated Factors. Infection and Drug Resistance, 14, 4567-4580.

418. Yuh T, Micheni M, Selke S, Oluoch L, Kiptinness C, Magaret A, Chohan B, Ngure K, Wald A, Mugo NR, Roxby AC. 2020. Sexually Transmitted Infections Among Kenyan Adolescent Girls and Young Women With Limited Sexual Experience. Frontiers in Public Health, 8.

419. Zaki ME, Raafat D, El Emshaty W, Azab MS, Goda H. 2010. Correlation of Trichomonas vaginalis to bacterial vaginosis: a laboratory-based study. Journal of Infection in Developing Countries, 4(3), 156-163.

420. Zhang XJ, Shen Q, Wang GY, Yu YL, Sun YH, Yu GB, Zhao D, Ye DQ. 2009. Risk factors for reproductive tract infections among married women in rural areas of Anhui Province, China. Eur J Obstet Gynecol Reprod Biol, 147(2), 187-91.

421. Zhang ZF, Graham S, Yu SZ, Marshall J, Zielezny M, Chen YX, Sun M, Tang SL, Liao CS, Xu JL, et al. 1995. Trichomonas vaginalis and cervical cancer. A prospective study in China. Ann Epidemiol, 5(4), 325-32.

422. Zhao FH, Forman MR, Belinson J, Shen YH, Graubard BI, Patel AC, Rong SD, Pretorius RG, Qiao YL. 2006. Risk factors for HPV infection and cervical cancer among unscreened women in a high-risk rural area of China. Int J Cancer, 118(2), 442-8.

423. Zhou H, Jia Y, Shen J, Wang S, Li X, Yang R, Huang K, Hu T, Tang F, Zhou J, Yuan J, Huang L, Tian X, Chen Z, Zhang Q, Wang C, Xi L, Deng D, Wang H, Ma D, Li S. 2014. Gynecologic infections seen in ThinPrep cytological test in Wuhan, China. Front Med, 8(2), 236-40.

424. Zhu X, Liu L, Yixi L, Yang Y, Zhang Y, Yang Z, Chen H, Dong J, Yang S. 2023. The prevalence and risk factors of Trichomonas vaginalis in Wuhan and the Tibetan area, China: a two-center study. Parasitology Research, 122(1), 265-273.

425. Zribi M, Ben Mansour K, Abid F, Masmoudi A, Fendri C. 2008. Syndromic approach to sexually transmitted infections in Tunisian women: Bacteriological validation. International Journal of STD and AIDS, 19(2), 112-114.
