# Supplementary material for: Systematic review and meta-analysis of the global prevalence and infection risk factors of Trichomonas vaginalis
Source: Parasite. 2025 Aug 27;32:56. doi: 10.1051/parasite/2025051 (PMC12386857; doi:10.1051/parasite/2025051)
Supplement: Supplementary file 1 — Supplementary file supplied by the authors. [file parasite-32-56-s1.zip › parasite240166-1-olm/Table S2.docx]

**Table S2.** The countries with one article reporting on the prevalence of *T. vaginalis.*

| **WHO region** | **NO.**  **Studies** | **Pooled prevalence**  **(95% CI)** | **WHO region** | **NO.**  **Studies** | **Pooled prevalence**  **(95% CI)** |
| --- | --- | --- | --- | --- | --- |
| Asia **region** | **8** | 8% (7%-9%) | North America **region** | **3** | 11% (8%-15%) |
| Kuwait | 1 | 12 | Honduras | 1 | 10% |
| Philippines | 1 | 10% | Nicaragua | 1 | 13% |
| Palestine | 1 | 14% | Greenland | 1 | 1% |
| Japan | 1 | 3% | South America **region** | **4** | 5% |
| Yemen | 1 | 1% | Ecuador | 1 | 0% |
| Nepal | 1 | 7% | Chile | 1 | 2% |
| Malaysia | 1 | 1% | Grenada | 1 | 1% |
| Azerbaijan | 1 | 6% | Paraguay | 1 | 10% |
| Africa **region** | **10** | 12% (10%-14%) | Europe **region** | **9** | 3% (3%-4%) |
| Mozambique | 1 | 31% | Greece | 1 | 5% |
| Durban | 1 | 9% | Germany | 1 | 16% |
| Tunisia | 1 | 3% | Poland | 1 | 1% |
| Swaziland | 1 | 8% | Cyprus | 1 | 0.4% |
| Congo | 1 | 14% | Slovenia | 1 | 0.20% |
| Eswatini | 1 | 25% | Portugal | 1 | 31% |
| Malawi | 1 | 13% | Bulgaria | 1 | 9% |
| Ethiopia | 1 | 3% | Croatia | 1 | 8% |
| Gambia | 1 | 4% | France | 1 | 2% |
| Lebanon | 1 | 11% |  |  |  |
