# Supplementary material for: Systematic review and meta-analysis of the global prevalence and infection risk factors of Trichomonas vaginalis
Source: Parasite. 2025 Aug 27;32:56. doi: 10.1051/parasite/2025051 (PMC12386857; doi:10.1051/parasite/2025051)
Supplement: Supplementary file 1 — Supplementary file supplied by the authors. [file parasite-32-56-s1.zip › parasite240166-1-olm/Table S3.docx]

**Table S3.** Subgroup analysis of the prevalence of *T. vaginalis* in various China and USA.

| **Region** | **NO.**  **Studies** | **Pooled prevalence (95% CI)** | **Heterogeneity**  **Q- value *I^2^*  *p*-value** | | |
| --- | --- | --- | --- | --- | --- |
| **China** |  |  |  |  |  |
| Beijing | 2 | 1% (0%-2%) | 3.79 | 73.59 | 0.05 |
| Shanxi | 1 | 10.7% | 0.00 | 0.00 | 1 |
| Shandong | 3 | 3% (3%—4%) | 11.11 | 82.00 | 0 |
| Hubei | 2 | 15% (5%—26%) | 17.13 | 94.16 | 0 |
| Henan | 1 | 1.6% | 0.00 | 0.00 | 1 |
| Anhui | 1 | 4.5% | 0.00 | 0.00 | 1 |
| Chongqing | 1 | 6.7% | 0.00 | 0.00 | 1 |
| Sichuan | 2 | 11% (6%—27%) | 73.79 | 98.64 | 0 |
| Qinghai | 1 | 10.5% | 0.00 | 0.00 | 1 |
| Yunnan | 8 | 11% (6%—15%) | 236.98 | 97.05 | 0 |
| Hebei | 1 | 13% | 0.00 | 0.00 | 1 |
| Guangdong | 2 | 1% (1%—4%) | 14.25 | 92.98 | 0 |
| Fujian | 1 | 3.2% | 0.00 | 0.00 | 1 |
| Taiwan | 3 | 2% (1%—4%)) | 72.9 | 95.88 | 0 |
| Tibet | 1 | 20.4% | 0.00 | 0.00 | 1 |
| Guang xi | 1 | 0.38% | 0.00 | 0.00 | 1 |
| **American** |  |  |  |  |  |
| New York | 5 | 21% (15%—26%) | 111.73 | 96.42 | 0 |
| Ohio | 5 | 15% (10%—20%) | 56.65 | 92.94 | 0 |
| Rhode | 1 | 22.0% | 0 | 0 | 1 |
| Georgia | 2 | 19% (6%—33%) | 9.90 | 89.90 | 0 |
| Tennessee | 1 | 4.4% | 0 | 0 | 1 |
| Maryland | 5 | 17% (9%—25%) | 196.35 | 97.96 | 0 |
| Louisiana | 2 | 17% (3%—38%) | 51.17 | 98.05 | 0 |
| Mississippi | 1 | 18.4% | 0 | 0 | 1 |
| California | 5 | 11% (2%—19%) | 194.01 | 98.26 | 0 |
| Wisconsin | 1 | 6.75% | 0 | 0 | 1 |
| Alabama | 4 | 15% (11%—19%) | 33.77 | 91.12 | 0 |
| Florida | 2 | 6% (1%—12%) | 13.99 | 92.85 | 0 |
| Carolina | 2 | 12% (1%—14%) | 0.46 | 0.11 | 0.5 |
| Washington | 1 | 3.7% | 0 | 0 | 1 |
