# Supplementary material for: Systematic review and meta-analysis of the global prevalence and infection risk factors of Trichomonas vaginalis
Source: Parasite. 2025 Aug 27;32:56. doi: 10.1051/parasite/2025051 (PMC12386857; doi:10.1051/parasite/2025051)
Supplement: Supplementary file 1 — Supplementary file supplied by the authors. [file parasite-32-56-s1.zip › parasite240166-1-olm/Figure S4.doc]

**Figure S4 :** The forest plots map representing the prevalence of *T. vaginalis* in Middle East and North Africa.
